# Workforce characteristics and interventions associated with high-quality care and support to older people with cancer: a systematic review

**DOI:** 10.1136/bmjopen-2017-016127

**Published:** 2017-07-31

**Authors:** Jackie Bridges, Grace Lucas, Theresa Wiseman, Peter Griffiths

**Affiliations:** 1 Faculty of Health Sciences, University of Southampton, Southampton, UK; 2 NIHR CLAHRC Wessex; 3 The Royal Marsden NHS Foundation Trust

**Keywords:** Neoplasms, Health manpower, Older people, Geriatric medicine, Oncology, Health services for the aged, Aged, Health personnel

## Abstract

**Objectives:**

To provide an overview of the evidence base on the effectiveness of workforce interventions for improving the outcomes for older people with cancer, as well as analysing key features of the workforce associated with those improvements.

**Design:**

Systematic review.

**Methods:**

Relevant databases were searched for primary research, published in English, reporting on older people and cancer and the outcomes of interventions to improve workforce knowledge, attitudes or skills; involving a change in workforce composition and/or skill mix; and/or requiring significant workforce reconfiguration or new roles. Studies were also sought on associations between the composition and characteristics of the cancer care workforce and older people's outcomes. A narrative synthesis was conducted and supported by tabulation of key study data.

**Results:**

Studies (n=24) included 4555 patients aged 60+ from targeted cancer screening to end of life care. Interventions were diverse and two-thirds of the studies were assessed as low quality. Only two studies directly targeted workforce knowledge and skills and only two studies addressed the nature of workforce features related to improved outcomes. Interventions focused on discrete groups of older people with specific needs offering guidance or psychological support were more effective than those broadly targeting survival outcomes. Advanced Practice Nursing roles, voluntary support roles and the involvement of geriatric teams provided some evidence of effectiveness.

**Conclusions:**

An array of workforce interventions focus on improving outcomes for older people with cancer but these are diverse and thinly spread across the cancer journey. Higher quality and larger scale research that focuses on workforce features is now needed to guide developments in this field, and review findings indicate that interventions targeted at specific subgroups of older people with complex needs, and that involve input from advanced practice nurses, geriatric teams and trained volunteers appear most promising.

**Strengths and limitations of this study**

- This novel synthesis provides evidence of promising interventions targeted at delivering high-quality care to older people with cancer.
- It is an international systematic review articulating the evidence base on workforce interventions that may support high-quality cancer care to an expanding ageing population.
- The review is limited to those studies where the role of the workforce was explicitly articulated within an intervention; there may be other studies in which changes or adaptations to the workforce were tested but not reported and are not included here.
- The review only included items published in the English language.

## Background

More than 60% of new cancers and more than 70% of cancer deaths occur in people over the age of 65 years in Europe and the USA.^1^ Treatment outcomes for older patients with cancer vary internationally^2^ and this may be linked to the extent to which services and their associated workforce effectively meet the more complex needs associated with an ageing population.^3 4^ Many older people have comorbidities and limitations which affect their cognitive and physical functioning, their risk of complications and their emotional well-being,^5^ all of which may affect cancer treatment tolerance and necessitate a modified treatment plan and relevant supportive care.^6^ More comprehensive assessment and management has been recommended to optimise older patients with cancer for treatment.^6–8^ Furthermore, older people may value a range of outcomes beyond survival at any cost, including maintaining independence and being able to access information, emotional support and practical support both during and after treatment.^9^ Healthcare workers who organise and deliver cancer care thus need knowledge of clinical and other issues which are common in old age, but also need to be adept with the skills and values to enable them to support the patient and family, develop treatment plans, deliver appropriate care and help older people to achieve the quality of life (QOL) that reflects what matters most to them as individuals.^10^

While the specific role of the healthcare workforce in ensuring optimal outcomes and QOL for older cancer survivors and their families has been recognised, evidence suggests that there are variations internationally in the preparedness of the workforce to meet the needs of an ageing population.^9–16^ Issues identified include deficits in the necessary education, knowledge, skills and attitudes; in staffing levels and skill mix; and in the development of roles, teams and services that meet older people's needs.^17^ However, little is known about the features and characteristics of the workforce associated with better outcomes for older people with cancer, or about the relative effectiveness of workforce-focused interventions which are aimed at improving cancer care and outcomes for an ageing population. This systematic review therefore aims to inform developments in policy and practice by providing an overview of the evidence base on the effectiveness of workforce interventions for improving the outcomes for older people with cancer, as well as analysing key features of the workforce associated with those improvements.

## Methods

Systematic methods were used to guide searching, selection and analysis.^18^ Searches for primary research evaluating workforce interventions for older people with cancer were undertaken in August 2016. Studies were identified by searching electronic databases, scanning reference lists of articles and by contacting study authors. A detailed search strategy was tested in MEDLINE (table 1). The search was additionally tailored for database-specific subject headings and applied in: PsycINFO, Cumulative Index to Nursing and Allied Health Literature (CINAHL), Allied and Complementary Medicine Database (AMED), Embase, Web of Science, Cochrane Central Register of Controlled Trials (CENTRAL), AgeInfo and Scopus (see online supplementary file 1). Searches were limited to the English language. No date limit was applied to ensure a comprehensive overview of developments in the field. The PRISMA (Preferred Reporting Items for Systematic Reviews and Meta-Analyses) guidelines have been used to guide reporting (see online supplementary file 2).^19^

10.1136/bmjopen-2017-016127.supp1 Supplementary data 1

10.1136/bmjopen-2017-016127.supp3 Supplementary data 3

**Table 1:** Example of search strategy for MEDLINE (EBSCOHOST)

Concept 1	Concept 2	Concept 3
1. TI Elderly OR AB Elderly	10. TI Cancer OR AB Cancer	14. TI Workforce OR AB Workforce
2. TI Geriatric* OR AB Geriatric*	11. TI Oncolog* OR AB Oncolog*	15. TI ‘Health professionals’ OR AB ‘Health professionals’
3. TI ‘Older people’ OR AB ‘Older people’	12. MM Neoplasms	16. TI ‘Healthcare professionals’ OR AB ‘Healthcare professionals’
4. TI ‘Older patient*’ OR AB ‘Older patient*’	13. 10 or 11 or 12	17. TI ‘Health care professionals’ OR AB ‘Health care professionals’
5. TI ‘Older person’ OR AB ‘Older person’		18. TI ‘Health personnel’ OR AB ‘Health personnel’
6. TI ‘Older adult*’ OR AB ‘Older adult*’		19. TI ‘Healthcare personnel’ OR AB ‘Healthcare personnel’
7. MM Aged		20. TI ‘Health care personnel’ OR AB ‘Health care personnel’
8. MM Frail Elderly		21. TI ‘Medical personnel’ OR AB ‘Medical personnel’
9. 1 or 2 or 3 or 4 or 5 or 6 or 7 or 8		22. TI ‘Advanced Practice nurse’ OR AB ‘Advanced Practice Nurse’
		23. TI ‘Clinical nurse specialist’ OR AB ‘Clinical nurse specialist’
		24. TI Geriatrician* OR AB Geriatrician*
		25. TI Gerontologist* OR AB Gerontologist*
		26. TI ‘Allied health professionals’ OR AB ‘Allied health professionals’
		27. TI Training
		28. TI Educat*
		29. TI ‘Skill mix’ OR AB ‘Skill mix’
		30. TI ‘Grade mix’ OR AB ‘Grade mix’
		31. TI ‘Staff development’ OR AB ‘Staff development’
		32. TI Staff* W1 level* OR AB Staff* W1 level*
		33. TI Teamwork OR AB Teamwork
		34. MM Health manpower
		35. MM Health personnel
		36. MM Attitude of Health personnel
		37. MM Professional Competence
		38. MM Staff development
		39. MM Education, professional
		40. MM Nurse's role
		41. MM Geriatric assessment
		42. MM Health services for the aged
		43. or/14–42
		44. 9 AND 13 AND 43
		45. English language filter

Eligible study types included randomised controlled trials (RCTs), quasiexperimental or observational studies with a clearly defined workforce variable or intervention with a comparison between different exposure levels, and qualitative studies evaluating features of the workforce from the perspective of older people with cancer and where the role of the workforce forms a central part of the research question. We defined workforce-based interventions as any intervention where the main mode of action was through a change in the composition, roles, knowledge, skills or attitudes of individuals or groups in a care delivery role, paid or unpaid, not including family or informal caregivers. Papers included reported on studies conducted with participants identified as older people (age 60+) at any stage in the cancer journey (from targeted screening through to end of life). Papers included reported on either:

- outcomes of interventions to improve the knowledge, attitudes or skills of the workforce delivering cancer care and treatment to older people;
- outcomes of interventions involving a change in the composition and/or skill mix of the workforce delivering cancer care for older people including (but not limited to) role substitution, new roles or adding specialist practitioners to the team;
- outcomes of interventions routinely targeted at older people with cancer, which were reported to require significant workforce reconfiguration or the implementation of new roles;
- associations between the composition and characteristics of the cancer care workforce (including, but not limited to, staffing levels, skill mix, training, knowledge attitudes and skill) and outcomes for older people with cancer.

Studies reporting solely on drug, treatment or other therapeutic interventions (without specific focus on the workforce delivering those interventions) were not included.

**Figure 1:**
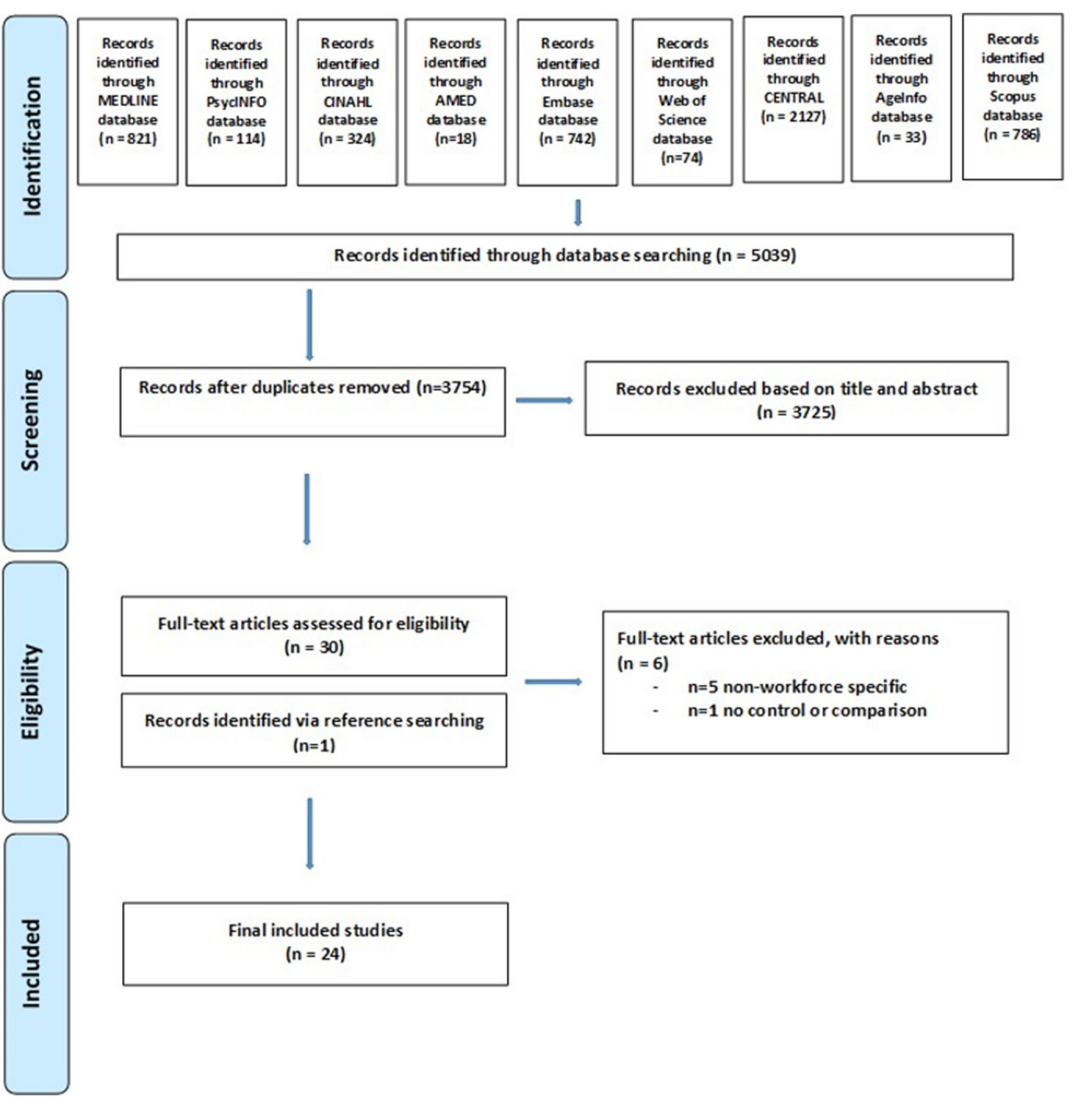
PRISMA (Preferred Reporting Items for Systematic Reviews and Meta-Analyses) study selection flow chart.

Titles and abstracts from the searches were screened against the inclusion criteria by GL to exclude irrelevant papers. Five per cent of titles/abstracts were also independently reviewed by another team member (JB, PG or TW) to confirm exclusion decisions. Full-text papers were retrieved for all papers that screened positively against inclusion criteria or about which a clear decision could not be taken (due to lack of information). Each full-text paper was reviewed independently by two team members followed by a decision to include or exclude. These reviews were followed by further team discussion to finalise inclusion. The search and selection process is summarised in the PRISMA flow chart (figure 1).^19^

Data on aim, design, setting, sample, intervention, outcome and results were extracted systematically from eligible papers using data extraction tables developed by the team (see online supplementary file 3). We adapted the GRADE (Grading of Recommendations Assessment, Development and Evaluation) system as used by Cochrane for rating evidence^18^ to guide a broad assessment of individual study quality and thereby the contribution studies made to the review. Initial quality ratings based on study design were upgraded or downgraded depending on presence of factors considered to strengthen or weaken the evidence. Two members of the team independently reviewed all included papers. Discrepancies were discussed and ratings confirmed through discussions involving both raters and a third team member. No studies were excluded based on this assessment but lower quality studies were given less weight in the analysis.

10.1136/bmjopen-2017-016127.supp2 Supplementary data 2

Due to the heterogeneity of interventions and outcomes, a narrative analysis of study findings was merited.^20^ Studies were grouped around the patient or service problems the interventions were targeting. Results were tabulated and the findings of effectiveness of individual interventions were plotted within these groups and used as the basis for an analysis of the strength of evidence of effectiveness across these groups and the field as a whole. We recorded and tabulated both the direction of differences between groups (where reported) and statistical significance of differences. Due to the number of different outcomes across the 24 studies, we report, within the Results section, for the primary outcomes where there is evidence of significant differences between groups, rather than narrating the full set of results for each individual paper. A review protocol is available from the study team on request.

## Results

We identified 24 eligible published journal papers (23 quantitative and 1 qualitative study) covering 22 interventions and reporting on 4555 patient participants age 60+ from targeted screening, through cancer diagnosis and treatment and beyond. All but one study were conducted in USA or Europe. The studies report on 27 individual primary outcomes and 42 individual secondary outcomes (using a range of measures) across the studies corresponding to 41 different outcomes in total (n=38 of these were patient-related outcomes and the other 3 outcomes were focused directly on the workforce). As detailed below and illustrated in table 2, 17 studies were assessed as low or very low quality, with 4 studies rated as medium and 3 as high quality.

**Table 2:** Summary of studies included in review

Source; study design; final quality rating	Setting and sample	Intervention and workforce	Primary outcomes (secondary outcomes)	Results
Regular and timely access to care and treatment				
Basu *et al*^25^ Observational: care control ++	Women aged 61+ with breast cancer, n=86 One cancer centre, USA	I: Patient navigation: support and coordination of patient care T: From point of diagnosis to survivorship clinic C: No navigation W: Breast cancer nurse	Time from diagnosis to oncology appointment	Time to consultation decreased by 4.9 days (p=0.0002)
Goodwin *et al.*^27^ RCT ++++	Women aged 65+ newly diagnosed with breast cancer, n=335 13 community and 2 public hospitals, USA	I: Case management: nurse as educator, counsellor, advocate and care coordinator T: 12 months service C: Usual care (unclear) W: Nurse case manager	Treatment received in 6 months after breast cancer diagnosis (patient satisfaction; arm function)	More intervention women saw radiation oncologist (36% vs 19.3%) (p=0.006), received more breast-conserving surgery (28.6% vs 18.7%; p=0.031) and radiation therapy (36.0% vs 19.0%; p=0.003), had more breast reconstruction surgery (9.3% vs 2.6%, p=0.054); reported choice in treatment (82.2% vs 69.9%, p=0.020) No differences between groups in percentages who saw an oncologist, discussed breast reconstruction, underwent complete surgical staging, or tissue sent for hormone receptor assay
Mandelblatt *et al*^35^ Controlled before and after study ++	Women aged 65+ screening for breast or cervical cancer, n=673 Two public hospitals, USA	I: Screening intervention during routine visits T: At scheduled appointments C: Physician reminder system W: Nurse practitioner	Annual screening rates for Pap tests and mammographies	Annual intervention site Pap test rate increased (17.8%–56.9%), and mammographies (18.3%–40%) compared with control site increase of Pap test rate from 11.8% to 18.2% and no change (18%) for mammography (p=0.01)
Somana-Ehrminger *et al*^32^ Observational ++	Women aged 75+ with breast cancer, n=206 Breast and gynaecological cancer registry, France	I: Geriatrician referral and treatment plan C: Patients with no GOC W: Geriatrician, dietitian, psychologist, physical therapist or social worker	Independent impact of GOC	GOC patients more likely to receive mastectomy and adjuvant therapy (p<0.0001); and less likely to be treated by breast-conserving surgery and adjuvant therapy (p=0.003)
Complications and specific problems of cancer treatment				
Bourdel-Marchasson *et al*^34^ RCT ++	Chemotherapy patients aged 70+, n=336 12 public and private settings, France	I: Face-to-face dietary counselling T: 6 visits (3–6 months) C: Usual care W: Dietitian	1-year mortality (chemotherapy management; unplanned hospitalisation; 2-year mortality)	Difference of 178 kcal/day dietary intake in intervention group (p<0.01) No difference in other outcomes
Hempenius *et al*^30^ RCT ++	Frail adults aged 65+, elective surgery for solid tumour, n=260 Teaching hospital and community hospital, Netherlands	I: Delirium prevention: assessment, monitoring and individualised treatment plan T: During hospital stay C: Usual care. Additional geriatric care provided on referral W: Geriatric team supervised by a geriatrician	Incidence of postoperative delirium up to 10 days (severity of delirium; length of hospital stay; complications; mortality; care dependency; QOL)	Significant difference in return to preoperative living situation (67.3% vs 79.1%, OR: 1.84, 95% CI 1.01 to 3.37) No significant difference in other outcomes
Kalsi *et al*^44^ Observational ++	Adults aged 70+ with cancer, n=135 One hospital, UK	I: Geriatrician CGA and intervention plan for identified need T: Prechemotherapy and further support as needed C: Standard oncology care W: Geriatrician	CGA impact on chemotherapy tolerance and toxicity; rate of planned completion of cancer (treatment modifications; early treatment discontinuation; death at 6 months)	Intervention more likely to complete planned cancer treatment (33.8% vs 11.4%, OR 4.14 (95% CI 1.50 to 11.42), p=0.006) and fewer required treatment modifications (43.1% vs 68.6%, OR 0.34 (95% CI 0.16 to 0.73), p=0.006) Lower toxicity rate in intervention group (43.8% vs 52.9%, p=0.292) No differences in death rates
McCorkle *et al*^37^ RCT ++++	Adults aged 60+ with postsurgical cancer, n=375 Comprehensive cancer centre, USA	I: Specialised home care APNs assess and monitor physical, emotional and functional status of patients, provide direct care, access services and other resources from the community, and provide teaching, counselling and support during recovery T: 4 weeks with three home visits and five telephone contacts C: Usual follow-up care in an ambulatory setting and routine outpatient follow-up W: APNs	Length of survival (depressive symptoms; symptom distress; functional status)	Late-stage patients, improved 2-year survival in intervention group: 66.7% vs 39.6% (p<0.05). No difference for early-stage patients Risk of death higher for control (adjusted HR 2.04; 95% CI 1.33 to 3.12; p=0.001) compared with intervention group No differences between groups in other outcomes
Comorbidities and complex health needs				
Deliens *et al.*^33^ Uncontrolled before and after study +	Adults aged 70+ with cancer (non-haematological) hospitalised, n=91 Geriatric oncology unit, tertiary hospital, Belgium	I: Medication review: identification of PIMs and drug interactions T: From point of admission and during hospitalisation C: Before to after W: Clinical pharmacist	PIMs using START and STOPP criteria (drug-to-drug interactions)	START criteria: 41 PIMs for 31 patients (34%) at hospital admission compared with 7 PIMs for 6 persons (7%) at discharge STOPP criteria: 50 PIMs for 29 patients (32%) at admission compared with 16 PIMs for 14 persons (16%) at discharge
Fann *et al*^26^ RCT +++	Adults aged 60+ with diagnosis of non-skin cancer and major depression or dysthymia, n=215 18 primary care clinics at 8 diverse healthcare organisations, USA	I: Depression management: education, ‘behavioural activation’, treatment support. T: Up to 12 months. Follow-up usual care 12 months more C: Usual care: received routinely available depression treatment W: Depression care manager (nurse or clinical psychologist) collaborative with primary care	Depression treatment response (health-related QOL; health-related impairments: work, family, social functioning; patient satisfaction)	Intervention twice as likely to experience a depression treatment response at 12 months than control (39% vs 20%; p=0.029) and at 18 months (38% vs 16%; p=0.012) Remission rates higher in intervention group versus control group at 6 months (32% vs 16%, p=0.006) and 12 months (22% vs 9%, p=0.031) Less functional impairment at 12 months (p=0.011) and greater QOL (p=0.039)
Herr *et al*^22^ RCT (cluster) +++	Adults aged 65+ with cancer receiving hospice care, n=738 Staff: nurses (n=383 pre, n=415 post) and physicians (n=16) 16 hospices, USA	I: Workforce: to promote adoption of evidence-based pain practices. Included: training, assessment of data, champion input, senior leadership engagement T: Engagement phase 5 months, 12-month intervention C: Hospices received clinical practice guidelines W: 3 days training. Selection of local pain facilitators, nurse and physician champions, grant expert nurse input, nurse and physician champion	Workforce: adoption of evidence-based cancer pain practices (pain severity)	No significant difference in improvement on cancer pain practice index between intervention and control Decrease in patient pain severity from pre to post in intervention group greater (p=0.1032)
Johansson *et al*^41^ RCT ++	Adults aged 70+ newly diagnosed with prostate, GI or breast cancer, n=161 Primary healthcare services, Sweden (other participants reported: n=255 under 70 years)	I: Intensified primary healthcare. Individual support: nurse support, nutritional support and individual psychological support. T: Starting from diagnosis C: Standard care + group rehabilitation W: Home care nurse, dietitian and psychologist. GPs and nurses trained in pain, nausea and diet in final-stage life	Utilisation of specialist care	Mean days of hospitalisation for older intervention patients than control (3.8 vs 8.9, p<0.01) 4 of 82 older intervention patients admitted compared with 12 of 79 older control patients (p<0.05) 10 out of 82 made acute visits to outpatient clinics compared with 22 of 79 in control group (p<0.05)
Rao *et al*^31^ RCT +++	Adults aged 65+ with cancer, frail and hospitalised, n=99 11 medical centres, USA	I: Assessment and monitoring by geriatric team: (1) geriatric inpatient + usual outpatient; (2) usual inpatient + geriatric outpatient; (3) geriatric inpatient and outpatient T: 1-year study C: Usual care: all hospital services except from geriatric team W: Core team: geriatric medicine attending physician, fellow or intern, a nurse practitioner, social worker	Survival; health-related QOL (functional status; physical performance)	No difference in survival for patients with cancer regardless of treatment group Significant effect of geriatric inpatient care versus usual inpatient care: mean change in score from randomisation to discharge: bodily pain (28.7 vs 10.1), p=0.09; emotional limitation (29.3 vs 2.7), p=0.01. Effect on bodily pain sustained at 1 year (37.6 vs 9.9) No effect of geriatric outpatient care on any of the QOL parameters No effect of either inpatient or outpatient geriatric care on the functional status of patients with cancer
QOL, physical and psychological functioning				
Chock *et al*^40^ (secondary analysis of Clark *et al*^66^) RCT ++	Adults aged 65+ with advanced cancer treated with radiotherapy, n=16 Cancer centre, USA (other participants reported: n=38 under 65 years)	I: QOL intervention with telephone follow-up: physical therapy, education, cognitive behavioural interventions, discussion and support, spiritual reflection and relaxation training T: 6 sessions 90 min, 2–4 weeks and 10 brief structured telephone sessions C: Standard care W: Multidisciplinary (including physical therapist, clinical psychologist, APN, chaplain)	QOL; mood	Significant difference at week 4 only in mean overall QOL older versus younger adults (74.4 vs 62.9, p=0.040) Significantly lower anger-hostility dimension of mood measure at all weeks for older versus younger patients. Week 4: 95.0 vs 86.4, p=0.028; week 27: 92.2 vs 84.2, p=0.027; week 52: 96.3 vs 85.9, p=0.005 No other significant differences
Heidrich *et al*^38^ Two pilot RCTs and one observational study ++	Women aged 65+, 1 year postdiagnosis of non-metastatic breast cancer, n=82 (total) Oncology clinics, cancer centre, USA	I: Pilot 1—symptom management (IRIS): counselling interview and telephone follow-up on symptom management at 4 weeks; pilot 2—addition of four biweekly telephone reinforcement sessions; pilot 3—intervention by phone only C: (1) usual care; (2) delayed IRIS (waitlist) control; (3) no control (IRIS group only) T: 4 weeks (pilot 1) W: APN	Feasibility, acceptability (symptom distress; symptom management; QOL; mood; barriers to symptom management; communication difficulty)	Feasibility: across all studies, 76% of eligible women participated, 95% completed the study, 88% reported the study was helpful and 91% were satisfied with the study Pilot 1: no significant difference in symptom distress. Significant decrease in distress baseline to follow-up in intervention group; significantly more women in intervention reported changing self-care of symptoms (p<0.05); no significance QOL differences Pilot 2: significant less symptom duration compared with control at 8 weeks (p<0.01). At 16 weeks, intervention group more likely to have talked to healthcare provider, begun new symptom treatment and changed self-care symptoms (p<0.05). No significant QOL differences. Negative attitudes from healthcare providers reported by 5%–20% of women and communication difficulties by 5%–45% of women Pilot 3: No significant differences (no control) from baseline to 8 weeks. Symptom interference decreased (and negative mood from symptoms). Symptom duration interference and negative mood from symptoms decreased. No QOL change
Kornblith *et al*^28^ RCT ++	Adults aged 65+ with breast, colon or prostate cancer, n=131 Cancer centres/university settings, USA	I: Telephone monitoring of distress providing support (plus educational materials) T: Over 6 months—monthly monitoring C: Educational materials alone, referred to oncology nurse upon evaluation if distressed significantly W: Trained graduates monitoring telephone calls. Referral onto an oncology nurse where indicated	Psychological distress	Lower anxiety and depression mean HADS total score for intervention 6.01 vs 8.20 control (p<0.0001); HADS depression subscale, intervention 3.20 vs 4.08 control (p=0.0004); HADS anxiety subscale intervention 2.81 vs 3.25 control (p<0.0001), at 6 months controlling for study entry levels No differences on other measures of psychological distress
Lapid *et al*^42^ Secondary age group analysis of Rummans *et al*^67^ RCT ++	Adults aged 65+ newly diagnosed with advanced cancer, n=33 Cancer centre, USA	I: Multidisciplinary psychosocial QOL sessions T: Eight 90 min sessions, 4 weeks after enrolment C: Standard care (regular outpatient visits with oncologist and allied healthcare providers) W: Led by psychiatrist or psychologist and cofacilitated by a nurse, physical therapist, chaplain or social worker. Leaders trained in materials and observed sessions	QOL	Higher overall QOL intervention group scores throughout the study, not significant Higher QOL scores at week 4 intervention versus control (79.3 vs 62.9, p=0.0461) Improvement in QOL scores for intervention at weeks 4 and 8 compared with older control group
Mantovani *et al*^29^ RCT ++	Adults aged 65+ with cancer, n=72 Inpatient setting at medical oncology clinic, Italy	I1. Emotional and practical support from volunteers and I2. with structured psychotherapy T: I2: Weekly sessions of 1 hour for 6 months C: Pharmacological only W: Trained volunteers basic=40 hours/6 months, another 40 hours/6 months practical and further personal training	QOL	Non-significant between group differences in functional status/physical symptom improvements over time: Karnofsky's Performance Status Scale (*F*=9.90, 2 df, p<0.001). No differences on Spitzer's QOL Index or Functional Living Index—within/between groups Significant between group differences: State-Trait Anxiety Inventory control significantly worsened and intervention groups significantly improved (I1 improved more than I2) (*F*=4.50, 2 df, p<0.05) Beck Depression Inventory: control group unchanged, both intervention groups improved (*F*=229.66, 2 df, p<0.01)
Sajid *et al*^43^ RCT (pilot) ++	Men aged 70+ with prostate cancer and hormone therapy, n=19 Two medical oncology clinics, USA	I1. EXCAP (home-based walking and resistance intervention) I2. Technology-mediated walking and resistance intervention using Wii Fit T: One face-to-face session then 6–12 weeks home based C: Usual care W: Trained exercise physiologist	Functional and aerobic (skeletal muscle and muscular mass measure; handgrip strength; chest repetition test; DEXA scan)	EXCAP intervention arm higher rate of change in steps per day at each follow-up (+2720 steps) (p<0.01) compared with control (+97 steps) and Wii Fit arm (+382 non- significant) EXCAP arm had a 2.3 point change in physical battery score after 12 weeks, compared with 0.6 points in the Wii Fit arm and −0.5 points in the usual care arm No other significant differences in outcomes
Suh *et al*^39^ RCT +++	Adults aged 65+ completed active treatment for gastrointestinal cancers, n=63 Cancer centre, South Korea	I: 8 weeks of Qi exercise and 1 hour face-to-face counselling on physical and psychological factors T: 8 weeks C: Usual care W: Two Qi exercise trainers, APNs	Physical activity (BMI; body weight; nutritional status; symptom experience; self-efficacy; self-esteem)	Physical activity increased in both groups, extent of increase greater in intervention group (p=0.005) Difference in amount of exercise over time between groups (p=0.002) No between-group difference in BMI Nutritional status in both groups improved over time. The degree of reduction, however, was significantly larger in intervention group (p=0.048), and same in interaction between group and time Both group and interaction factors have significant positive difference in symptom experience, health promotion and self-esteem for intervention
Yagli and Ulger^36^ Controlled before and after study ++	Women aged 65–70, 6 months after chemotherapy for breast cancer, n=20 Department of physiotherapy and rehabilitation, Turkey	I: 8 sessions of 1-hour yoga classes T: 8 weeks C: Exercise programme for 8 weeks W: Existing physiotherapist (yoga teacher)	QOL; depression levels; levels of pain, fatigue and sleep quality	All patients' QOL scores improved pre to postyoga and exercise interventions Total scores and some subcategories of the Nottingham Health Profile showed significant difference in favour of the yoga group (p<0.05) but not on energy level and pain where there were no differences Significant better fatigue and sleep quality in yoga group postintervention (p<0.05)
Communication between patients and healthcare professionals				
Devik *et al*^23^ Qualitative ++	Adults aged 65+ with advanced cancer, n=9 Patients’ homes in rural Norway	I: Qualitative study of home nursing care to patients with advanced cancer in rural locations C: NA W: District nurses in normal role	Patient experience	Importance of nurses having a person-centred manner Ability to show a genuine and empathic interest in the patients Technical skills or special competences less discussed than personal qualities, such as having a sense of humour or generosity Good listening and communication skills
van Weert *et al*^21^ RCT (cluster) ++++	Adults aged 65+ with cancer receiving chemotherapy, n=210 Staff: oncology-trained nurses, n=77 12 wards of 10 hospitals, Netherlands	I: Workforce: communication skills training in delivery of chemotherapy education to patients T: 3-month implementation C: Nurses continued to provide patient education as usual W: Nursing and specialised oncology nursing roles	Effects on quality of staff communication; effects on content of the consultation (patient recall of information)	Significant improvement in discussing realistic expectations. C: −0.20; I: 0.45 (total between-group difference 0.65) (p<0.01) Significant decrease in rehabilitation information pre to postchange. C: 0.08; I: −0.38 (total between-group difference −0.45) (p<0.01) No significant changes in categories treatment-related information and coping information Non-significant: intervention group showed significant decrease in number of items discussed Less history taking pre to post (C: 1.83; I: −2.33; between-group difference −4.17; p<0.001) and less talking about all different side effects pre to postchange (C: 1.98; I: −5.71; total 7.68; p<0.001) Patients in intervention asked more questions (M=10.76) than control (M=6.69; p<0.05) Marginal significance for intervention group: proportion recall of recommendations post versus pre (C: −3.34; I: 6.39; total: 9.73; p<0.10)
Yeom and Heidrich^24^ Observational ++	190 women at least 1 year postbreast cancer diagnosis, n=190 Community, an oncology clinic and a state tumour registry, USA	I: Symptom management (IRIS): counselling interview and telephone follow-up on symptom management T: 8-week intervention with 16-week follow-up point in the RCT C: Waitlist control subjects offered intervention after 16-week follow-up assessment W: APN	Negative beliefs about symptom management (QOL; purpose in life; positive relations with others)	Significant direct effects on SMBQ (p<0.00) and Communication Attitudes Questionnaire (p=.012) or Communication Difficulties Questionnaire Communication difficulties significant direct, negative effects on all four dimensions of QOL Significant total effects of SMBQ on MCS (mental QOL) (p=0.001) and PIL and PR (p<0.001) but not physical component (PCS). SMBQ predicted lower levels of QOL in three of four dimensions None of the four indirect effects of SMBQ on QOL through CommD was significant, indicating that CommD does not mediate the effects of SMBQ on QOL The total effects of CommA on four QOL measures were not significant. However, the indirect effects for MCS (p=0.05), PIL (p<0.05) and PR (p<0.05) through CommD were significant, indicating that CommD mediates the effects of CommA on MCS, PIL and PR

Quality ratings: high ++++; moderate +++; low ++; very low + intervention/workforce description.

APN, advanced practice nurse; BMI, body mass index; C, control group; CGA, comprehensive geriatric assessment; df, degrees of freedom; DEXA, dual-energy X-ray absorptiometry; EXCAP home-based walking and resistance intervention; GI gastrointestinal; GOC, geriatric oncology consultation; GP, general practitioner; HADS Hospital Anxiety and Depression Scale; I, intervention; IRIS individualized representational intervention to improve symptom management; MCS, mental component summary; NA, not applicable; PCS, physical component summary; PIL, purpose in life; PIM, potentially inappropriate medications; PR, personal relations; QOL, quality of life; RCT, randomised controlled trial; SMBQ, Symptom Management Beliefs Questionnaire; START, screening tool to alert doctors to right treatment; STOPP, screening tool of older person's potentially inappropriate prescriptions; T, time point; W, workforce involved.

The point of the cancer journey each intervention was targeted at varied widely. Interventions ranged from targeted screening stage (n=1) and from diagnosis (n=4); to treatment phase/hospital stay (n=11); to those primarily focused on patients after the completion of their treatment (n=6); hospice care (n=1) or home care for patients with advanced cancer (n=1). The majority of the interventions were limited to specific tumour types: 15 involved participants with a range of cancer types, but some involved more homogeneous populations: 6 were for patients with breast cancer, 1 intervention targeted patients with prostate cancer, another involved those with gastrointestinal cancers and 1 was aimed at breast and cervical screening.

Only two interventions were directly targeted at improving the knowledge, attitudes or skills of the workforce delivering cancer care and treatment to older people through training^21 22^ and only two studies directly addressed the second objective of the review to assess the salient features of the cancer care workforce: one qualitative study considered the features of the nursing workforce which older patients felt were important in their care^23^ and one study looked at the impact of healthcare professionals communication on participants’ views about their symptom management.^24^ The remaining studies reported on improving older people's outcomes via interventions involving a change in the workforce. In five interventions new roles were tested: nurse navigator,^25^ depression care manager,^26^ nurse case manager,^27^ telephone support (trained graduates)^28^ and social support volunteers.^29^ In other studies, support from additional workforce members was provided to patients. Four studies reported on the increased involvement of a geriatrician or a geriatrics team,^27 30–32^ one reported on the input of a clinical pharmacist^33^ and one study reported on the input of an additional dietitian.^34^ In two studies, a current staff member had a different function; in one study a nurse provided targeted cancer screening^35^ and in another study a physiotherapist designed exercise and yoga programmes.^36^ Three interventions used advanced practice nurses (APN)—one in a home care capacity^37^ and two in counselling roles.^38 39^ In three studies, the role of multidisciplinary team members was highlighted.^40–42^ In some papers, although a named member or members of the workforce were reported to have implemented or carried out the intervention, it was unclear as to the exact nature of their position. This was the case with two studies using exercise physiologists where it could not be determined if they were existing or new staff members.^39 43^ Only seven studies referred to an explicit theoretical framework or model in intervention design.^21 22 24–26 38 39^

Because of the heterogeneity of studies retrieved (and the small number of studies that addressed the review's second objective), we reviewed evidence of the effectiveness of interventions by study type established through particular problems (related to older people with cancer) that the respective interventions were addressing and, subsequently, ways in which workforce requirements were being adapted to meet needs and improve outcomes related to these patient problems. The results in table 2 and set out below are displayed using these individual types.

### Regular and timely access to care and treatment

Four studies focused on interventions targeted at the problem of systemic delays or inequitable access to treatment in the cancer journey for older people. They provide some promising evidence that providing additional support to some groups of older patients with cancer can help them navigate the system and access treatment thereby improving the speed and efficacy of care. However, three of these papers provide only low-quality evidence.

A high-quality RCT reported that older women with breast cancer in the care of a nurse case manager acting as an educator, counsellor and coordinator were significantly more likely to see a radiation oncologist as part of initial evaluation, and to receive breast-conserving surgery and radiation therapy.^27^ Further, the difference in receipt of appropriate treatment between women with characteristics associated with lower rates of appropriate treatment (75+, being unmarried, living alone and being a member of an ethnic minority group) and their respective comparison groups were diminished or eliminated in the intervention group. An observational study reported that a breast cancer nurse navigator providing support and coordination of patient care from diagnosis until entry into survivorship clinic significantly shortened time to consultation for patients aged 61+ years.^25^ A nurse practitioner role was used in a quasiexperimental study to improve screening rates for older Black women of low socioeconomic status by offering screening during a routine visit.^35^ Nurse practitioner follow-up screening rates were significantly higher than baseline, compared with control group follow-up rates. A further study assessed the impact of a geriatrician consultation and treatment plan through an analysis of registry data of older patients with breast cancer.^32^ Patients who had a consultation had more comorbidities and more advanced and aggressive tumours, were more likely to receive mastectomy and adjuvant therapy, and were less likely to be treated by breast-conserving surgery and adjuvant therapy.

### Complications and specific problems from cancer treatment

Four studies reported the use of workforce members with specialist skills to address cancer treatment complications and impact on mortality and survival. None of the three low-quality studies found any intervention effect on mortality rates, but the one high-quality RCT found that specialised home care APN (used to enhance surgical recovery) increased 2-year survival for patients with late-stage cancer in the intervention group.^37^

Other lower quality studies in this group included evaluations of face-to-face counselling to address nutritional intake for patients treated with chemotherapy and at risk of malnutrition,^34^ an intervention focused on the prevention of postoperative delirium with input from a geriatric team^30^ and comprehensive geriatric assessment (CGA) targeted at chemotherapy tolerance and toxicity.^44^ The observational study evaluating CGA for older chemotherapy patients found that CGA patients were more likely to complete cancer treatment as planned but no significant differences were found in relation to mortality or other outcome measures in relation to the interventions in any of these three studies.

### Comorbidities and complex health needs

The five studies reported here target the health issues that may accompany a cancer diagnosis, but also broader health problems that may not directly relate to the cancer. They highlight the importance of recognising and addressing these needs, although the range of outcomes and the variable quality of evidence (three studies of medium quality; two were low quality) make it difficult to draw firm conclusions about the best use of workforce support in this sizeable area.

A cluster RCT evaluating a hospice staff training programme on improving pain assessment and management did not find significant practice improvements or decreases in patient pain severity associated with the intervention.^22^ In a different study, a secondary analysis of RCT data on the impact of a depression care manager providing education and support for older patients with depression found that intervention patients with a cancer diagnosis were twice as likely to experience a depression treatment response at 12 months compared with usual care.^26^ Rao *et al* also reviewed the outcomes for patients with cancer from a wider RCT evaluating the impact of involving a geriatric team in the care of inpatients and outpatients diagnosed with frailty.^31^ The inpatient intervention group showed significant improvements in bodily pain and mental health versus the usual inpatient care group but there was no impact on survival rates. There were no intervention effects on outpatients. An uncontrolled before and after study reported that using a clinical pharmacist to identify patients’ potentially inappropriate medications (PIMs) reduced the number of PIMs at discharge versus admission.^33^ A low-quality RCT reported that intensified primary healthcare support significantly reduced the number of days in hospital for an intervention group of patients with advanced cancer compared with patients receiving standard care.^41^

### QOL, physical and psychological functioning

Eight studies focused on addressing QOL across its physical and psychological aspects. This group of interventions used a range of workforce members (often in therapeutic or supportive roles) from physiotherapists to APN to trained voluntary input, to address a range of factors underpinning QOL. They showed mixed evidence of effectiveness. Seven of the studies in this group provided low-quality evidence.

Three studies focused on physical functioning in particular. In an RCT with low recruitment rate and possible selection bias, exercise physicians provided Qi exercise training.^39^ Both usual care and intervention participants increased their activity levels but the extent of the increase was significantly greater in the intervention group. The intervention also used APN delivering face-to-face counselling and significant improvements in symptom experience, self-efficacy and self-esteem were reported. A controlled before and after study compared the effect of yoga classes (with the input of a physiotherapist/yoga teacher) with a standard exercise programme.^36^

QOL scores after the programme were better than before for both groups, but some QOL parameters improved more for those included in the yoga intervention. A pilot RCT with small sample and high dropout compared two exercise forms implemented by a physiologist (compared with usual care) and found significant activity increases for the group using a home-based walking and resistance intervention.^43^

Two similar interventions involved a multidisciplinary team approach for a range of QOL domains; however, both of these secondary analyses reported on very small sample sizes of older adults within wider QOL interventions. Lapid *et al*^42^ found in a secondary analysis of a small sample of patients in a wider RCT, that higher QOL scores were reported for older patients who received multidisciplinary emotional and practical support. However, in the study by Chock *et al*,^40^ the authors did not find any lasting differences on QOL for older intervention participants against their younger counterparts, apart from an improvement in anger-hostility.

APNs were used in a symptom management intervention in the two pilot RCTs and the observational study reported by Heidrich *et al.*^38^ Some evidence of effectiveness was reported for improving self-care and reducing symptom distress and duration, but there was no impact on QOL.

Two studies used trained volunteers to bolster psychological support. A secondary analysis of RCT data was used to evaluate the effect of using trained graduate support workers to provide initial distress monitoring to patients over the telephone.^28^ Intervention patients had significantly lower anxiety and depression at 6 months than patients receiving educational materials alone. However, no other differences in psychological well-being were detected. Mantovani *et al*^29^ also used trained support volunteers to provide emotional and practical support. An RCT with small sample size was used to compare this support with pharmacological treatment alone, and further with the addition of psychotherapy. Significant improvements in anxiety and depression were reported for the groups receiving voluntary support and/or additional psychotherapy. However, there were no significant differences on other QOL measures.

### Communication between healthcare professionals and older people with cancer

Three studies focused on addressing the communication needs of older people with cancer. One high-quality study offered communication skills training to staff with varied success^21^ and the other two low-quality studies highlighted the importance of good communication as a prerequisite for cancer nurses related to improving older patients’ QOL.

A cluster RCT found that training nursing staff to improve chemotherapy patient education led to a significant, positive effect for ‘discussing realistic expectations.’^21^ Significantly less history taking was also observed pre to post in the intervention group, as well as less talking about all the possible side effects; both points of attention during training. No other significant effects were reported. Yeom and Heidrich^24^ used a cross-sectional analysis of RCT data to report that communication difficulties with health professionals had significant direct, negative effects on QOL dimensions. Findings from a qualitative interview study highlighted the value to older patients with cancer of nurses having a person-centred manner, with the ability to show a genuine and empathic interest in the patients and to make a connection with good listening and communication skills.^23^

## Discussion

This systematic review aimed to provide an overview of the evidence base on the effectiveness of workforce interventions for improving the outcomes for older people with cancer, as well as analysing key features of the workforce associated with those improvements. Findings reflect a range of ways in which the workforce has been adapted, expanded or trained to addressing older patients with cancer multiple and divergent needs. The findings present a novel synthesis of the type of interventions being developed globally to address the broad question of how the workforce can support the improvement of older people's cancer outcomes. The approaches and the patient problems they are addressing are varied, including integrating the input of geriatric specialists into cancer services, using APN roles to support patients, creating new roles to guide patients through the healthcare system and ensuring effective treatment, through to novel approaches using voluntary support, or trialling yoga or other exercise to improve older patients’ QOL.

While the included studies begin to provide evidence about how the workforce can be used to make a tangible difference to physical and psychological outcomes of older patients with cancer, the diversity of interventions in the studies reviewed and the range of outcomes evaluated limit generalisations on effectiveness. Further, the quality of evidence is generally low. Experimental designs were not consistently used and, when they were, their implementation was often hampered by poorer than expected recruitment, or conclusions drawn about outcomes for older patients were drawn from a secondary analysis of a wider data set. In addition, as is common in the reporting of complex intervention evaluations, details of the intervention itself were often lacking.^45^ There was inadequate reporting of the specific workforce contribution to the interventions and limited evidence to address the second objective of the review around the features of the cancer care workforce associated with better outcomes. In addition, while staff training was involved in half of the interventions reported, the details of how that training worked or could be improved were not detailed. Furthermore, although some innovative roles were set up, the rationale and detail of those roles were often poorly reported.

Despite these shortcomings, these findings do provide some promising insights into how the workforce may address the varied needs of older patients with cancer, although with a dearth of evidence at the earlier and later stages of the cancer journey. Evidence has suggested that not all older people with cancer need the same input, and indeed age-related changes occur at different rates in different individuals and are not reflected in chronological age.^7^ Therefore, it is more productive to focus attention on those with complex problems.^46^ The studies in this review appear to support the notion of targeted assistance to groups at particular risk of undertreatment. Review findings suggest that broader interventions aiming to improve survival outcomes are less successful, but studies did indicate the kind of support that could be put in place after treatment to deal with the specific complications and problems that older people might face. One intervention which did improve survival used APN in home care support postsurgery.^37^ Indeed, the role of APN in the future of older people's cancer care has been acknowledged elsewhere in the literature,^47–50^ and this review indicates that this is a candidate role for exploration and further consideration.

The input of geriatric specialists who are able to assess and manage older patients and optimise patients for treatment was a significant feature of several studies reviewed and formal links and services are well established in some countries.^51–53^ Findings from this review provide weak evidence of positive benefits from the input of geriatricians but it only included studies where the geriatrician's role was explicit in the intervention and where a comparison or control was featured. There are a number of other reviews reporting on specialist geriatric assessment and management for older patients with cancer, and these have been able to draw firmer conclusions about the benefits of CGA with older patients with cancer, although they all acknowledge the need for more definitive research.^54–56^ Multidisciplinary approaches also emerged as a feature across the studies reviewed and the need to shape teams around the multiple needs of older people with cancer has been highlighted elsewhere, although evidence from this review is weak, again limited by the scale and quality of the research.^6 57–60^

Of further interest is the use of non-professionals in providing direct care services to older people with cancer, and roles such as these are relevant in the contexts of budgetary pressures and recruitment difficulties of key professional groups such as geriatricians and registered nurses.^17^ The two studies reviewed suggested a positive impact on patient outcomes and align with a growing recognition of the non-clinical workforce (including carers and families) playing an essential role in older people's cancer care.^61–63^ However, the low quality of the research again reduces confidence in these positive findings. A final point is that the studies identified for this review did not address the impact of staffing levels or skill mix on outcomes of older patients with cancer. In addition, few mechanisms to develop the current workforce to prepare for and be supported to deliver high-quality care to an ageing population were identified. In addition to the development and more definitive evaluation of new roles and practices, the future research agenda must address these important facets to ensure that, regardless of setting, all healthcare workers that older people with cancer encounter are prepared for and adequately supported in their role.^64 65^

This review alone is insufficient to enable conclusions to be drawn about the workforce factors which prove most beneficial to older people's outcomes; further high-quality RCTs are needed to assess the potential of possible interventions. Future research should build on the studies reviewed here to establish what workforce developments are needed to support this growing population throughout the cancer journey. The most promising interventions for further study target assistance to individuals with complex needs who are at particular risk of undertreatment, and of problems arising from cancer treatment or its impact. Our review indicates that the impact of multiprofessional teams, including geriatric physicians and APN, on patient outcomes from survival to QOL, would be worthwhile to evaluate more definitively, as would the contribution of trained volunteers.

## Supplementary Material

Reviewer comments

Author's manuscript

## References

[1] Yancik R, Ries LA Cancer in older persons: an international issue in an aging world. Semin Oncol 2004;31:128–36. 10.1053/j.seminoncol.2003.12.024 15112144

[2] Coleman MP, Forman D, Bryant H, et al ICBP Module 1 Working Group. Cancer survival in Australia, Canada, Denmark, Norway, Sweden, and the UK, 1995-2007 (the International Cancer Benchmarking Partnership): an analysis of population-based Cancer registry data. Lancet 2011;377:127–38. 10.1016/S0140-6736(10)62231-3 21183212 PMC3018568

[3] International Society of Geriatric Oncology. SIOG 10 priorities Initiative: SIOG, 2011 http://www.siog.org/content/siog-10-priorities-initiative (accessed 24 Mar 2017).

[4] Lynch MP, DeDonato DM, Kutney-Lee A Geriatric Oncology Program Development and Gero-Oncology Nursing. Semin Oncol Nurs 2016;32:44–54. 10.1016/j.soncn.2015.11.006 26830267

[5] Prouse J, Phillips J Care of older people living with Cancer: the role of the specialist nurse and alied health profesionals. Cancer Forum 2013;37:226–9.

[6] Bridges J, Hughes J, Farrington N, et al Cancer treatment decision-making processes for older patients with complex needs: a qualitative study. BMJ Open 2015;5:e009674 10.1136/bmjopen-2015-009674 PMC4679903 26667015

[7] Balducci L, Extermann M Management of Cancer in the older person: a practical approach. Oncologist 2000;5:224–37. 10.1634/theoncologist.5-3-224 10884501

[8] Caillet P, Laurent M, Bastuji-Garin S, et al Optimal management of elderly Cancer patients: usefulness of the Comprehensive Geriatric Assessment. Clin Interv Aging 2014;9:1645–60. 10.2147/CIA.S57849 25302022 PMC4189720

[9] Expert Reference Group for the Older Person with Cancer. Older people living with cancer: designing the future health care workforce: Macmillan Cancer Support, 2016 http://sogweb-prod.neox24.ch/files/public/workforce-report.pdf (accessed 24 Mar 2017).

[10] Bridges J, Wengström Y, Bailey DE Educational Preparation of Nurses Caring for Older people with Cancer: an International Perspective. Semin Oncol Nurs 2016;32:16–23. 10.1016/j.soncn.2015.11.003 26830264

[11] Independent Cancer Taskforce. Achieving world-class Cancer outcomes. A strategy for England 2015-2020. 2015 https://www.cancerresearchuk.org/sites/default/files/achieving_world-class_cancer_outcomes_-_a_strategy_for_england_2015-2020.pdf (accessed 24 Mar 2017).

[12] Lyman GH Caring for the elderly Cancer patient: training the next generation of oncologists. J Oncol Pract 2008;4:193–4. 10.1200/JOP.0841503 20856772 PMC2793945

[13] Rao AV, Hurria A, Kimmick G, et al Geriatric oncology: past, present, future. J Oncol Pract 2008;4:190–2. 10.1200/JOP.0846001 20856771 PMC2793949

[14] Maggiore RJ, Gorawara-Bhat R, Levine SK, et al Perceptions, attitudes, and experiences of hematology/oncology fellows toward incorporating geriatrics in their training. J Geriatr Oncol 2014;5:106–15. 10.1016/j.jgo.2013.10.003 24484724

[15] Foubert J, Faithfull S Education in Europe: are Cancer nurses ready for the future? J Buon 2006;11:281–4. 17309150

[16] McMillan S, Grodzicki BK, Shahrokni A Building a Comprehensive Educational Model for nurse practitioner students in Geriatric Oncology. J Am Geriatrics Soc 2016;64:S180–S80.

[17] Bridges J, Lucas G, Wiseman T, et al Preparedness of UK workforce to deliver Cancer care to older people: summary report from a scoping review. University of Southampton. 2016 http://eprints.soton.ac.uk/401192/ (accessed 24 Mar 2017).

[18] Guyatt GH, Oxman AD, Vist GE, et al GRADE: an emerging consensus on rating quality of evidence and strength of recommendations. BMJ 2008;336:924–6. 10.1136/bmj.39489.470347.AD 18436948 PMC2335261

[19] Moher D, Liberati A, Tetzlaff J, et al Preferred reporting items for systematic reviews and meta-analyses: the PRISMA statement. PLoS Med 2009;6:e1000097 10.1371/journal.pmed.1000097 19621072 PMC2707599

[20] Popay J, Roberts H, Sowden A, et al Guidance on the conduct of narrative synthesis in systematic reviews. A product from the ESRC methods programme version 1: ESRC. 2006.

[21] van Weert JC, Jansen J, Spreeuwenberg PM, et al Effects of communication skills training and a Question Prompt Sheet to improve communication with older Cancer patients: a randomized controlled trial. Crit Rev Oncol Hematol 2011;80:145–59. 10.1016/j.critrevonc.2010.10.010 21075644

[22] Herr K, Titler M, Fine PG, et al The effect of a translating research into practice (TRIP)--Cancer intervention on Cancer pain management in older adults in hospice. Pain Med 2012;13:1004–17. 10.1111/j.1526-4637.2012.01405.x 22758921 PMC3422373

[23] Devik SA, Hellzen O, Enmarker I "Picking up the pieces" - Meanings of receiving home nursing care when being old and living with advanced cancer in a rural area. Int J Qual Stud Health Well-being 2015;10:28382–82. 10.3402/qhw.v10.28382 26362533 PMC4567585

[24] Yeom HE, Heidrich SM Relationships between three beliefs as barriers to symptom management and quality of life in older breast Cancer survivors. Oncol Nurs Forum 2013;40:E108–18. 10.1188/13.ONF.E108-E118 23615144 PMC3870139

[25] Basu M, Linebarger J, Gabram SG, et al The effect of nurse navigation on timeliness of breast Cancer care at an academic comprehensive Cancer center. Cancer 2013;119:2524–31. 10.1002/cncr.28024 23585059

[26] Fann JR, Fan MY, Unützer J Improving primary care for older adults with Cancer and depression. J Gen Intern Med 2009;24(Suppl 2):417–24. 10.1007/s11606-009-0999-4 PMC2763171 19838842

[27] Goodwin JS, Satish S, Anderson ET, et al Effect of nurse case management on the treatment of older women with breast Cancer. J Am Geriatr Soc 2003;51:1252–9. 10.1046/j.1532-5415.2003.51409.x 12919237

[28] Kornblith AB, Dowell JM, Herndon JE, et al Telephone monitoring of distress in patients aged 65 years or older with advanced stage Cancer: a Cancer and leukemia group B study. Cancer 2006;107:2706–14. 10.1002/cncr.22296 17078057

[29] Mantovani G, Astara G, Lampis B, et al Impact of psychosocial intervention on the quality of life of elderly Cancer patients. Psychooncology 1996;5:127–35. 10.1002/(SICI)1099-1611(199606)5:2<127::AID-PON220>3.0.CO;2-L

[30] Hempenius L, Slaets JP, van Asselt D, et al Outcomes of a Geriatric Liaison intervention to prevent the development of postoperative delirium in frail elderly Cancer patients: report on a Multicentre, Randomized, Controlled Trial. PLoS One 2013;8:e64834 10.1371/journal.pone.0064834 23840308 PMC3686791

[31] Rao AV, Hsieh F, Feussner JR, et al Geriatric evaluation and management units in the care of the frail elderly Cancer patient. J Gerontol A Biol Sci Med Sci 2005;60:798–803. 10.1093/gerona/60.6.798 15983186

[32] Somana-Ehrminger S, Dabakuyo TS, Manckoundia P, et al Influence of geriatric oncology consultation on the management of breast Cancer in older women: a French population-based study. Geriatr Gerontol Int 2015;15:111–9. 10.1111/ggi.12240 24456152

[33] Deliens C, Deliens G, Filleul O, et al Drugs prescribed for patients hospitalized in a geriatric oncology unit: potentially inappropriate medications and impact of a clinical pharmacist. J Geriatr Oncol 2016;7:463–70. 10.1016/j.jgo.2016.05.001 27238734

[34] Bourdel-Marchasson I, Blanc-Bisson C, Doussau A, et al Nutritional advice in older patients at risk of malnutrition during treatment for chemotherapy: a two-year randomized controlled trial. PLoS One 2014;9:e108687 10.1371/journal.pone.0108687 25265392 PMC4181649

[35] Mandelblatt J, Traxler M, Lakin P, et al A nurse practitioner intervention to increase breast and cervical Cancer screening for poor, elderly black women. the Harlem Study Team. J Gen Intern Med 1993;8:173–8. 10.1007/BF02599260 8515326

[36] Yagli NV, Ulger O The effects of yoga on the quality of life and depression in elderly breast Cancer patients. Complement Ther Clin Pract 2015;21:7–10. 10.1016/j.ctcp.2015.01.002 25697379

[37] McCorkle R, Strumpf NE, Nuamah IF, et al A specialized home care intervention improves survival among older post-surgical Cancer patients. J Am Geriatr Soc 2000;48:1707–13. 11129765 10.1111/j.1532-5415.2000.tb03886.x

[38] Heidrich SM, Brown RL, Egan JJ, et al An individualized representational intervention to improve symptom management (IRIS) in older breast Cancer survivors: three pilot studies. Oncol Nurs Forum 2009;36:E133–E143. 10.1188/09.ONF.E133-E143 19403441 PMC2754203

[39] Suh EE, Kim H, Kang J, et al Outcomes of a culturally responsive health promotion program for elderly korean survivors of gastrointestinal cancers: a randomized controlled trial. Geriatr Nurs 2013;34:445–52. 10.1016/j.gerinurse.2013.09.007 24156925

[40] Chock MM, Lapid MI, Atherton PJ, et al Impact of a structured multidisciplinary intervention on quality of life of older adults with advanced Cancer. Int Psychogeriatr 2013;25:2077–86. 10.1017/S1041610213001452 24001635 PMC4364551

[41] Johansson B, Holmberg L, Berglund G, et al Reduced utilisation of specialist care among elderly Cancer patients: a randomised study of a primary healthcare intervention. Eur J Cancer 2001;37:2161–8. 11677102 10.1016/s0959-8049(01)00278-7

[42] Lapid MI, Rummans TA, Brown PD, et al Improving the quality of life of geriatric Cancer patients with a structured multidisciplinary intervention: a randomized controlled trial. Palliat Support Care 2007;5:107–14. 10.1017/S1478951507070174 17578061

[43] Sajid S, Dale W, Mustian K, et al Novel physical activity interventions for older patients with prostate Cancer on hormone therapy: a pilot randomized study. J Geriatr Oncol 2016;7:71–80. 10.1016/j.jgo.2016.02.002 26916611 PMC4818675

[44] Kalsi T, Babic-Illman G, Ross PJ, et al The impact of comprehensive geriatric assessment interventions on tolerance to chemotherapy in older people. Br J Cancer 2015;112:1435–44. 10.1038/bjc.2015.120 25871332 PMC4453673

[45] Craig P, Dieppe P, Macintyre S, et al Developing and evaluating complex interventions: the New Medical Research Council guidance. BMJ 2008;337:a1655–83. 10.1136/bmj.a1655 18824488 PMC2769032

[46] Bridges J, Simcock R Meeting the workforce challenges for older people living with Cancer. Int J Nurs Stud 2017;65:A1–A2. 10.1016/j.ijnurstu.2016.11.013 27884390

[47] Kagan SH The Future of Gero-Oncology Nursing. Semin Oncol Nurs 2016;32:65–76. 10.1016/j.soncn.2015.11.008 26830269

[48] Levenson D Advanced practice nurses lengthen survival of elderly Cancer patients. Rep Med Guidel Outcomes Res 2001;12:5–7. 12053920

[49] Morgan B, Tarbi E The role of the Advanced Practice nurse in Geriatric Oncology Care. Semin Oncol Nurs 2016;32:33–43. 10.1016/j.soncn.2015.11.005 26830266

[50] Prouse J Geriatric assessment. the role of the specialist nurse practitioner. Asia-Pacific Journal of Clinical Oncology 2009;5:A155.

[51] Extermann M, Aapro M, Audisio R, et al Main priorities for the development of geriatric oncology: a worldwide expert perspective. J Geriatr Oncol 2011;2:270–3. 10.1016/j.jgo.2011.07.001

[52] Gosney M Contribution of the geriatrician to the management of Cancer in older patients. Eur J Cancer 2007;43:2153–60. 10.1016/j.ejca.2007.07.032 17855073

[53] Magnuson A, Dale W, Mohile S Models of Care in Geriatric Oncology. Curr Geriatr Rep 2014;3:182–9. 10.1007/s13670-014-0095-4 25587518 PMC4289627

[54] Puts MT, Santos B, Hardt J, et al An update on a systematic review of the use of geriatric assessment for older adults in oncology. Ann Oncol 2014;25:307–15. 10.1093/annonc/mdt386 24256847

[55] Hamaker ME, Schiphorst AH, ten Bokkel Huinink D, et al The effect of a geriatric evaluation on treatment decisions for older Cancer patients--a systematic review. Acta Oncol 2014;53:289–96. 10.3109/0284186X.2013.840741 24134505

[56] Extermann M, Hurria A Comprehensive geriatric assessment for older patients with Cancer. J Clin Oncol 2007;25:1824–31. 10.1200/JCO.2007.10.6559 17488980

[57] Lycke M, Pottel L, Boterberg T, et al Integration of geriatric oncology in daily multidisciplinary Cancer care: the time is now. Eur J Cancer Care 2015;24:143–6. 10.1111/ecc.12301 25711541

[58] Chapman AE, Swartz K, Schoppe J, et al Development of a comprehensive multidisciplinary geriatric oncology center, the Thomas Jefferson University experience. J Geriatr Oncol 2014;5:164–70. 10.1016/j.jgo.2014.01.003 24495585

[59] Bouzereau V, Le Caer F, Guardiola E, et al Experience of multidisciplinary assessment of elderly patients with Cancer in a French general hospital during 1 year: a new model care study. J Geriatr Oncol 2013;4:394–401. 10.1016/j.jgo.2013.04.006 24472485

[60] Terret C, Zulian GB, Naiem A, et al Multidisciplinary approach to the geriatric oncology patient. J Clin Oncol 2007;25:1876–81. 10.1200/JCO.2006.10.3291 17488986

[61] Mbah O, Ford JG, Qiu M, et al Mobilizing social support networks to improve Cancer screening: the COACH randomized controlled trial study design. BMC Cancer 2015;15:907 10.1186/s12885-015-1920-7 26573809 PMC4647280

[62] Fraser M Thinking differently about the Cancer workforce. 2016. http://www.nuffieldtrust.org.uk/blog/thinking-differently-about-cancer-workforce (accessed 24 Mar 2017).

[63] Glajchen M Education, Training, and Support Programs for Caregivers of Individuals with Cancer : Talley RC, McCorkle R, Baile WF, Cancer caregiving in the United States: research, Practice, Policy: rosalynn Carter Institute for Caregiving Caregiving: research-practice-policy series. springer: New York and Heidelberg, 2012:79–102.

[64] Muss HB Cancer in the Elderly: a Societal Perspective from the United States. Clin Oncol 2009;21:92–8. 10.1016/j.clon.2008.11.008 19059768

[65] Brodie H Getting the skill mix right. Nurs Older People 2013;25:10 10.7748/nop2013.07.25.6.10.s17 23901455

[66] Clark MM, Rummans TA, Atherton PJ, et al Randomized controlled trial of maintaining quality of life during radiotherapy for advanced Cancer. Cancer 2013;119:880–7. 10.1002/cncr.27776 22930253 PMC4405146

[67] Rummans TA, Clark MM, Sloan JA, et al Impacting quality of life for patients with advanced Cancer with a structured multidisciplinary intervention: a randomized controlled trial. J Clin Oncol 2006;24:635–42. 10.1200/JCO.2006.06.209 16446335

