# Supplementary material for: Workforce characteristics and interventions associated with high-quality care and support to older people with cancer: a systematic review
Source: BMJ Open. 2017 Jul 31;7(7):e016127. doi: 10.1136/bmjopen-2017-016127 (PMC5642668; doi:10.1136/bmjopen-2017-016127)
Supplement: Supplementary data 1 [file bmjopen-2017-016127supp001.pdf]

SUPPLEMENTARY FILE 1

Full search strategy

EBSCO HOST
Medline, CINHAL, PsycINFO, AMED: text terms and MeSH headings

1. TI Elderly OR AB Elderly
2. TI Geriatric* OR AB Geriatric*
3. TI “Older people” OR AB “Older people”
4. TI “Older patient*” OR AB “Older patient*”
5. TI “Older person” OR AB “Older person”
6. TI “Older adult*” OR AB “Older adult*”
7. MM Aged
8. MM Frail Elderly
9. **1 or 2 or 3 or 4 or 5 or 6 or 7 or 8**

10. TI Cancer OR AB Cancer
11. TI Oncolog* OR AB Oncolog*
12. MM Neoplasms
13. **10 or 11 or 12**

14. TI Workforce OR AB Workforce
15. TI “Health professionals” OR AB “Health professionals”
16. TI “Healthcare professionals” OR AB “Healthcare professionals”
17. TI “Health care professionals” OR AB “Health care professionals”
18. TI “Health personnel” OR AB “Health personnel”
19. TI “Healthcare personnel” OR AB “Healthcare personnel”
20. TI “Health care personnel” OR AB “Health care personnel”
21. TI “Medical personnel” OR AB “Medical personnel”
22. TI “Advanced Practice nurse” OR AB “Advanced Practice Nurse”
23. TI “Clinical nurse specialist” OR AB “clinical nurse specialist”
24. TI Geriatrician* OR AB Geriatrician*
25. TI Gerontologist* OR AB Gerontologist*
26. TI “Allied health professionals” OR AB “Allied health professionals”
27. TI Training
28. TI Educat*
29. TI “Skill mix” OR AB “Skill mix”
30. TI “Grade mix” OR AB “Grade mix”
31. TI “Staff development” OR AB “Staff development”
32. TI Staff* W1 level* OR AB Staff* W1 level*
33. TI Teamwork OR AB Teamwork
34. MM Health manpower
35. MM Health personnel
36. MM Attitude of Health personnel
37. MM Professional Competence
38. MM Staff development
39. MM Education, professional
40. MM Nurse’s role
41. MM Geriatric assessment

42. MM Health services for the aged
43. or/ 14-42
44. 9 AND 13 AND 43
45. English language filter

Embase (OVID)

1. elderly.ti,ab

2. geriatric.ti,ab

3. “older people”.ti,ab.

4. “older patient*”.ti,ab.

5. “older adult*”.ti,ab.

6. “older person”.ti,ab.

7. 1 or 2 or 3 or 4 or 5 or 6

8. cancer.ti,ab.

9. oncolog*.ti,ab.

1o. Neoplasm/

11. 8 or 9 or 10

12. workforce.ti,ab.

13. “health professionals”.ti,ab.

14. “healthcare professional*”.ti,ab.

15. “health care professional*”.ti,ab.

16. “health personnel”.ti,ab.

17. “healthcare personnel”.ti,ab.

18. “health care personnel”.ti,ab.

19. “medical personnel”.ti,ab.

20. “Advanced Practice nurse”.ti,ab.

21. “Clinical nurse specialist”.ti,ab.

22. Geriatrician*.ti,ab.

23. Gerontologist*.ti,ab.

24. “Allied health professionals”.ti,ab.

25. training.ti

26. educat*.ti

27. Skill Adj1 mix.ti,ab.

28. Grade Adj1 mix.ti,ab.

29. “staff development”.ti,ab.

30. “Workforce development”.ti,ab.

31. Staff adj1 level.ti,ab.

32. health care manpower/

33 professional competence/

34 vocational education/

35. health personnel attitude/

36. or/12-35

37. English.lg.

38. 7 AND 11 AND 36 AND 37

Scopus (Elsevier)

TITLE-ABS-KEY (cancer OR oncology OR neoplasm*) AND TITLE-ABS-KEY (elderly OR geriatric OR "older people" OR "older adult*" OR "older patient*" OR "older person") AND TITLE-ABS-KEY (workforce OR "health professional*" OR "health care professional*" OR “healthcare professional” OR "health personnel" OR "medical personnel" OR "advanced practice nurse" OR "clinical nurse specialist" OR geriatrician OR gerontologist OR "skill mix" OR "staff development" OR manpower)) AND (LIMIT-TO (LANGUAGE , "English"))

Web of Science (Thompson Reuters)

TITLE: (cancer OR oncolog* OR neoplasm*) AND TITLE: (elderly OR geriatric* OR "older people" OR "older person" OR "older adult*" OR "older patient*") AND TITLE: ("workforce" OR "health professional*" OR "healthcare professional*" OR “health care professional” OR "health personnel" OR "medical personnel" OR "advanced nurse practitioner" OR "clinical nurse specialist" OR geriatrician OR gerontologist OR "allied health professional*" OR training OR "educat*" OR "skill mix" OR "grade mix" OR "staff development")

Refined by: LANGUAGES: (ENGLISH)

Timespan=All years

CENTRAL

Elderly OR geriatric OR “older people” OR “Older adult*” OR “older patient*” OR “older person”

AND cancer OR oncolog* OR neoplasm*

In Title OR Abstract OR Key Words

Search for Trials

AgeInfo Cancer or Oncology in Title
