# Supplementary material for: Workforce characteristics and interventions associated with high-quality care and support to older people with cancer: a systematic review
Source: BMJ Open. 2017 Jul 31;7(7):e016127. doi: 10.1136/bmjopen-2017-016127 (PMC5642668; doi:10.1136/bmjopen-2017-016127)
Supplement: Supplementary data 2 [file bmjopen-2017-016127supp002.pdf]

SUPPLEMENTARY FILE 2 DATA EXTRACTION TABLE WITH EXAMPLE

STUDY AND AIM	STUDY DESIGN DATA COLLECTION MEASURES ANALYSIS	SETTING AND SAMPLE<br><br>COUNTRY CLINICAL SETTING PATIENT GROUPS STAFF GROUP	INTERVENTION	OUTCOMES	STAFF ROLE OR WORKFORCE DEVELOPMENT	RESULTS
Basu *et al.* (2013)<br><br>To evaluate timeliness in the period from breast cancer diagnosis to initial oncology consultation for a 9-month period prior to and following implementation of the nurse navigator.	Before and after study<br><br>Retrospective chart review of demographic data, tumour grade and stage, cancer treatment, appointment information, navigation status, time interval from diagnosis date to initial oncology appointment date.<br><br>Chi-square tests of independence used to compare by navigation group, and t tests used to compare continuous variables. Multiple linear	USA<br><br>One cancer centre<br><br>All women diagnosed within the institution with stage 0 to III breast cancer at initial presentation<br><br>176 F: 100 (57%) in prenavigation group) and 76 (43%) in post navigation group<br><br>Stratified by age <61 and 61+<br><br>In the 61+ age group there were 86 patients 52 in the pre navigation group<br>34 in the post	A dedicated breast cancer nurse navigator to assist patients with their care. The navigator's involvement begins when the patient is diagnosed and ends when the patient is appointed into the survivorship clinic.	The time interval from date of diagnosis to date of initial oncology appointment was measured in business days. Date of diagnosis was defined as the patient's first positive biopsy date. The date of initial oncology appointment was defined as the first date the patient had a consultation with a surgical, medical, or radiation oncologist. In a few	Collaborating with physicians and interdisciplinary team members to triage patients, coordinating patient care, educating patients and providing links to research and other relevant resources, and serving as the primary point of contact for patients and their families. The nurse navigator calls recently diagnosed patients within 24 hours of biopsy, meets patients at the initial visit, and assists patients in scheduling oncology	Overall, the median wait time was 10 days for all patients in the study and ranged from 1 to 32 days. The median wait time for patients in the prenavigation group was 11 days compared with 9 days for patients in the postnavigation group.<br><br>Because of interaction between navigation and age identified during the modeling process, stratified models presented for each of the 2 categories of age<br><br>For patients aged 61

SUPPLEMENTARY FILE 2 DATA EXTRACTION TABLE WITH EXAMPLE

STUDY AND AIM	STUDY DESIGN DATA COLLECTION MEASURES ANALYSIS	SETTING AND SAMPLE<br><br>COUNTRY CLINICAL SETTING PATIENT GROUPS STAFF GROUP	INTERVENTION	OUTCOMES	STAFF ROLE OR WORKFORCE DEVELOPMENT	RESULTS
	regression used to determine influence on time to consultation.	navigation group<br><br>Breast cancer nurse navigator		cases a phone call from the oncologist to discuss pathology results and treatment options was recorded as the consultation date if there was no other consultation before treatment.	appointments or any necessary diagnostic tests. Patients are then tracked during the course of care to ensure timeliness of appointments and therapies and to provide referrals to treatment and supportive services as needed.	years and older, the median wait time was 4 days shorter for the navigation group (8 days versus 12 days).<br><br>In the 61 or older age group, navigation was a significant predictor of time to consultation, adjusting for demographic and clinical factors (P<.0002). In this adjusted model, the unstandardized beta coefficient for navigation was 24.9, indicating that with navigation, time to consultation decreased by an average of almost 5 days
