# Supplementary material for: Workforce characteristics and interventions associated with high-quality care and support to older people with cancer: a systematic review
Source: BMJ Open. 2017 Jul 31;7(7):e016127. doi: 10.1136/bmjopen-2017-016127 (PMC5642668; doi:10.1136/bmjopen-2017-016127)
Supplement: Author's manuscript [file bmjopen-2017-016127.draft_revisions.pdf]

BMJ Open

Workforce characteristics and interventions associated with high quality care and support to older people with cancer: a systematic review

Journal:	*BMJ Open*
Manuscript ID	bmjopen-2017-016127
Article Type:	Research
Date Submitted by the Author:	25-Jan-2017
Complete List of Authors:	Bridges, Jackie; University of Southampton; NIHR CLAHRC Wessex<br>Lucas, Grace; University of Southampton, Faculty of Health Sciences<br>Wiseman, Theresa; Royal Marsden Hospital; University of Southampton, Faculty of Health Sciences<br>Griffiths, Peter; University of Southampton, Faculty of Health Sciences; NIHR CLAHRC Wessex
**Primary Subject Heading**:	Oncology
Secondary Subject Heading:	Geriatric medicine
Keywords:	Human resource management < HEALTH SERVICES ADMINISTRATION & MANAGEMENT, Adult oncology < ONCOLOGY, GERIATRIC MEDICINE

SCHOLARONE™
Manuscripts

For peer review only

TITLE PAGE

Workforce characteristics and interventions associated with high quality care and support to older people with cancer: a systematic review

Corresponding Author:
Professor Jackie Bridges
Professor of Older People's Care
Faculty of Health Sciences
University of Southampton
NIHR CLAHRC Wessex

Nightingale Building
University Road
Highfield Campus
Southampton SO17 1BJ
Tel.+44(0)23 8059 8282
Email jackie.bridges@soton.ac.uk

Grace Lucas
Faculty of Health Sciences
University of Southampton
Southampton
UK

Professor Theresa Wiseman
Faculty of Health Sciences
University of Southampton
Southampton
UK

Professor Peter Griffiths
Faculty of Health Sciences
University of Southampton
Southampton
UK

Word count (excluding title page, abstract, references, figures and tables): 4166

For peer review only

Workforce characteristics and interventions associated with high quality care and support to older people with cancer: a systematic review

ABSTRACT

Objectives: To systematically identify, describe and assess evidence on the effectiveness of workforce-based interventions in improving cancer care and treatment outcomes for older people and to analyse the features of the cancer care workforce associated with better outcomes for older people affected by cancer.

Design: Systematic review

Methods: Relevant databases were searched for primary research, published in English, reporting on older people and cancer and the outcomes of interventions to improve workforce knowledge, attitudes or skills; involving a change in workforce composition and/or skill mix; and/or requiring significant workforce reconfiguration or new roles. Studies were also sought on associations between the composition and characteristics of the cancer care workforce and older people's outcomes. A narrative synthesis was conducted and supported by tabulation of key study data.

Results: Studies (n=24) included 4,555 participants age 60+ from targeted cancer screening to end of life care. Interventions were diverse and two thirds of the studies were assessed as low quality. Only two studies directly targeted workforce knowledge and skills and only two studies addressed the nature of workforce features related to improved outcomes. Interventions focused on discrete groups of older people with specific needs offering guidance or psychological support were more effective than those broadly targeting survival outcomes. Advanced Practice Nursing roles, voluntary support roles and the involvement of geriatric teams provided some evidence of effectiveness.

Conclusions: An array of workforce interventions focus on improving outcomes for older people with cancer but these are diverse and thinly spread across the cancer journey. Higher quality and larger scale research that focuses on workforce features is now needed to guide developments in this field, and review findings indicate that interventions targeted at specific sub-groups of older people, and that involve input from advanced practice nurses, geriatric teams and voluntary workers appear most promising.

Keywords: Neoplasms, health manpower, older people, Geriatric medicine, Oncology, Health services for the aged, Nurse's role, Aged, health personnel

For peer review only

STRENGTHS AND LIMITATIONS OF THIS STUDY

- This novel synthesis provides evidence of promising interventions targeted at delivering high quality care to older people with cancer.
- It is an international review articulating the evidence base on workforce interventions that may support high quality cancer care to an expanding ageing population.
- However, within the reported studies, the definition of 'older people' is diverse and interventions for a range of cancer types are evaluated, with survivors at different stages of the cancer journey, limiting broader conclusions about effective workforce development.
- There is a need for high quality evidence about how the workforce can be used to best deliver and enhance older people's cancer care and what the particular features or characteristics of the workforce are most effective in improving older people's outcomes.
- The review is limited to those studies where the role of the workforce was explicitly articulated within an intervention; there may be other studies in which changes or adaptations to the workforce were tested but not reported and are not included here.

For peer review only

Workforce characteristics and interventions associated with high quality care and support to older people with cancer: a systematic review

BACKGROUND

More than 60% of new cancers and more than 70% of cancer deaths occur in people over the age of 65 years in Europe and the United States.[1] Treatment outcomes for older cancer patients vary internationally[2] and this may be linked to the extent to which services and their associated workforce effectively meet the more complex needs associated with an ageing population.[3 4] Many older people have comorbidities and limitations which affects their cognitive and physical functioning, their risk of complications and their emotional wellbeing[5] all of which may affect cancer treatment tolerance and necessitate a modified treatment plan and relevant supportive care.[6] More comprehensive assessment and management has been recommended to optimise older cancer patients for treatment.[6-8] Furthermore, older people may value a range of outcomes beyond survival at any cost, including maintaining independence and being able to access information, emotional support and practical support both during and after treatment.[9] Health care workers who organize and deliver cancer care thus need knowledge of clinical and other issues which are common in old age, but also need to be adept with the skills and values to enable them to support the patient and family, develop treatment plans, deliver appropriate care, and help older people to achieve the quality of life that reflects what matters most to them as individuals.[10]

While the specific role of the health care workforce in ensuring optimal outcomes and quality of life for older cancer survivors and their families has been recognised, evidence suggests that there are variations internationally in the preparedness of the workforce to meet the needs of an ageing population.[9-16] Issues identified include deficits in the necessary education, knowledge, skills and attitudes; in staffing levels and skillmix; and in the development of roles, teams and services that meet older people's needs.[17] However, little is known about the features and characteristics of the workforce associated with better outcomes for older people with cancer, or about the relative effectiveness of workforce-focused interventions which are aimed at improving cancer care and outcomes for an ageing population. This systematic review therefore aims to inform developments in policy and practice by systematically identifying, describing and assessing the effectiveness of workforce-based interventions (including randomised and non-randomised studies) in improving cancer care and treatment outcomes for older people. Secondarily, it aims to identify and analyse the features of the cancer care workforce associated with better outcomes in older people affected by cancer.

For peer review only

METHODS

Systematic methods were used to guide searching, selection and analysis.[18] Searches for primary research evaluating workforce interventions for older people with cancer were undertaken in August 2016. Studies were identified by searching electronic databases, scanning reference lists of articles and by contacting study authors. A detailed search strategy was tested in MEDLINE (Table 1). The search was additionally tailored for database-specific subject headings and applied in: PsycINFO, Cumulative Index to Nursing and Allied Health Literature (CINAHL), Allied and Complementary Medicine Database (AMED), Embase, Web of Science, Cochrane Central Register of Controlled Trials (CENTRAL), AgeInfo and Scopus (Supplementary File 1). Searches were limited to the English language. No date limit was applied.

We defined workforce based interventions as any intervention where the main mode of action was through a change in the composition, roles, knowledge, skills or attitudes of individuals or groups in a care delivery role, paid or unpaid, not including family or informal caregivers. Papers included in this review reported on studies conducted with participants identified as older people (age 60+) at any stage in the cancer journey (from targeted screening through to end of life). Papers included reported on either:

- Outcomes of interventions to improve the knowledge, attitudes or skills of the workforce delivering cancer care and treatment to older people;
- Outcomes of interventions involving a change in the composition and/or skill mix of the workforce delivering cancer care for older people including (but not limited to) role substitution, new roles or adding specialist practitioners to the team;
- Outcomes of interventions routinely targeted at older people with cancer, which require significant workforce reconfiguration or the implementation of new roles; and/or
- Associations between the composition and characteristics of the cancer care workforce (including, but not limited to, staffing levels, skill mix, training, knowledge attitudes and skill) and outcomes for older people with cancer.

Eligible study types included randomised controlled trials (RCTs), quasi-experimental or observational studies with a clearly defined workforce variable or intervention with a comparison between different exposure levels, and qualitative studies evaluating features of the workforce from the perspective of older people with cancer and where the role of the workforce forms a central part of the research question. Studies reporting solely on drug, treatment or other therapeutic

For peer review only - http://bmjopen.bmj.com/site/about/guidelines.xhtml

Table 1 Example of Search Strategy MEDLINE (EBSCOHOST)

Concept 1	Concept 2	Concept 3
1. TI Elderly OR AB Elderly	10. TI Cancer OR AB Cancer	14. TI Workforce OR AB Workforce
2. TI Geriatric* OR AB Geriatric*	11. TI Oncolog* OR AB Oncolog*	**15.** TI "Health professionals" OR AB "Health professionals"
3. TI "Older people" OR AB "Older people"	12. MM Neoplasms	**16.** TI "Healthcare professionals" OR AB "Healthcare professionals"
4. TI "Older patient*" OR AB "Older patient*"	13. 10 or 11 or 12	**17.** TI "Health care professionals" OR AB "Health care professionals"
5. TI "Older person" OR AB "Older person"		18. TI "Health personnel" OR AB "Health personnel"
6. TI "Older adult*" OR AB "Older adult*"		**19.** TI "Healthcare personnel" OR AB "Healthcare personnel"
7. MM Aged		**20.** TI "Health care personnel" OR AB "Health care personnel"
8. MM Frail Elderly		21. TI "Medical personnel" OR AB "Medical personnel"
9. 1 or 2 or 3 or 4 or 5 or 6 or 7 or 8		22. TI "Advanced Practice nurse" OR AB "Advanced Practice Nurse"
		23. TI "Clinical nurse specialist" OR AB "Clinical nurse specialist"
		24. TI Geriatrician* OR AB Geriatrician*
		25. TI Gerontologist* OR AB Gerontologist*
		26. TI "Allied health professionals" OR AB "Allied health professionals"
		27. TI Training
		28. TI Educat*
		29. TI "Skill mix" OR AB "Skill mix"
		30. TI "Grade mix" OR AB "Grade mix"
		31. TI "Staff development" OR AB "Staff development"
		32. TI Staff* W1 level* OR AB Staff* W1 level*
		33. TI Teamwork OR AB Teamwork
		34. MM Health manpower
		35. MM Health personnel
		36. MM Attitude of Health personnel
		37. MM Professional Competence
		38. MM Staff development
		39. MM Education, professional
		40. MM Nurse's role
		41. MM Geriatric assessment
		42. MM Health services for the aged
		43. or/ 14-42

For peer review only

		44. 9 AND 13 AND 43
		45. English language filter

interventions (without specific focus on workforce delivering those interventions) were not included.

Titles and abstracts from the searches were screened against the inclusion criteria by GL to exclude irrelevant papers. Five percent of titles/abstracts were also independently reviewed by another team member (JB, PG or TW) to confirm exclusion decisions. Full-text papers were retrieved for all papers that screened positively against inclusion criteria or about which a clear decision could not be taken (due to lack of information). Each full text paper was reviewed independently by two team members followed by a decision to include or exclude. These reviews were followed by further team discussion to finalise inclusion. The search and selection process is summarised in the PRISMA flow chart (Figure 1).[19]

Data on aim, design, setting, sample, intervention, outcome and results were extracted systematically from eligible papers using tables developed by the team (supplementary file 2). We adapted the GRADE system for rating evidence[18] to guide a broad assessment of individual study quality and thereby the contribution studies made to the review. Following GRADE guidance, initial quality ratings based on study design were upgraded or downgraded depending on presence of factors considered to strengthen or weaken the evidence. Two members of the team independently reviewed all included papers.

Discrepancies were discussed and ratings confirmed through discussions involving both raters and a third team member. No studies were excluded based on this assessment but lower quality studies were given less weight in the analysis.

Due to the heterogeneity of interventions and outcomes, a narrative analysis of study findings was merited. Studies were grouped around the patient or service problems the interventions were targeting. Results were tabulated and the findings of effectiveness of individual interventions were plotted within these groups and used as the basis for an analysis of the strength of evidence of effectiveness across these groups and the field as a whole. We recorded and tabulated both the direction of differences between groups (where reported) and statistical significance of differences. Due to the number of different outcomes across the 24 studies, we report, within the results section, for the primary outcomes where there is evidence of significant differences between groups, rather than narrating the full set of results for each individual paper. A review protocol is available from the study team on request.

For peer review only

Figure 1. PRISMA study selection flowchart

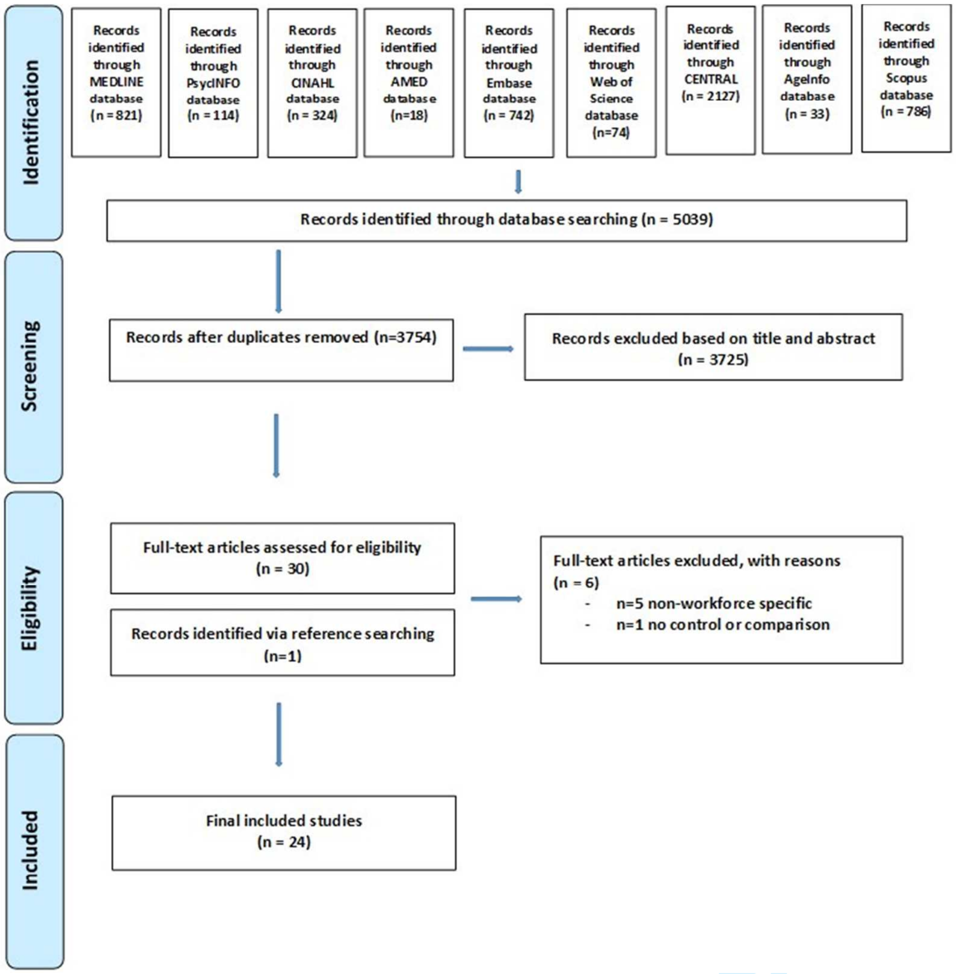

RESULTS

We identified 24 eligible journal papers (23 quantitative and 1 qualitative study) covering 22 interventions and reporting on 4,555 participants age 60+ from targeted screening, through cancer

For peer review only - http://bmjopen.bmj.com/site/about/guidelines.xhtml

diagnosis and treatment and beyond (Table 2). All but one study were conducted in USA or Europe. The studies report on 27 individual primary outcomes and 42 individual secondary outcomes (using a range of measures) across the studies corresponding to 41 different outcomes in total (n=38 of these were patient related outcomes and the other three outcomes were focused directly on the workforce). As detailed below, 17 studies were assessed as low or very low quality, with four studies rated as medium and three as high quality.

The point of the cancer journey each intervention was targeted at varied widely. Interventions ranged from targeted screening stage (n=1) and from diagnosis (n=4); to treatment phase/hospital stay (n=11); to those primarily focused on patients after the completion of their treatment (n=6); hospice care (n=1) or home care for advanced cancer patients (n=1). The majority of the interventions were limited to specific tumour types: 15 involved participants with a range of cancer types, but some involved more homogeneous populations: six were for breast cancer patients, one intervention targeted prostate patients, another involved those with gastrointestinal cancers, and one was aimed at breast and cervical screening.

Table 2 Included papers and quality appraisal
Key: High ++++; Moderate +++; Low ++; Very low +

	Study and design	Initial score based on study design	Factors leading to downgraded rating (where applicable)	Final rating
20	Basu et al 2013 Observational : case control	++	• No upgrading or downgrading	++
21	Bourdel-Marchasson et al 2013 RCT	++++	• Study stopped before completion of planned inclusions due to low recruitment<br>• Not blinded<br>• Power of analysis limited by recruitment issue	++
22	Chock et al 2013 RCT (secondary analysis of older adults)	++++	• Limited sample size<br>• Statistical analyses are exploratory and hypothesis generating only<br>• Single centre with homogeneous population	++
23	Deliens et al 2016 Observational: Uncontrolled before and after study	++	• Limited sample size, single site<br>• Prospective study, with no control group	+
24	Devik, Hellzen and Enmarker 2015, Qualitative	++	• No upgrading or downgrading (qualitative study)	++
25	Fann, Fan and Unutzer 2009 RCT (secondary analysis of cancer patients)	++++	• Secondary analysis so not prospectively randomized to the study groups<br>• Some cancer specific detail missing	+++
26	Goodwin et al 2003 RCT	++++	• No upgrading or downgrading	++++
27	Heidrich et al 2009 2 pilot RCTs and one observational study	+++	• Pilot: Single site and small samples<br>• Unclear randomization procedure<br>• Homogeneous sample in race and ethnicity	++
28	Hempenius et 2013 RCT	++++	• Nurses not blinded could have led to additional interventions in usual care group<br>• Study underpowered due to overall low rates of delirium	++

For peer review only

			• Inclusion rate lower than needed: 12% lost to follow up	
29	Herr et al 2012<br>RCT (cluster)	++++	• Not clear how randomization took place, or how to stop possible contamination<br>• Variance site to site engagement implementation intervention fidelity problematic<br>• Not generalizable beyond hospice setting	+++
30	Johansson et al 2001<br>RCT	++++	• 14% failed to completed trial<br>• Randomisation poorly reported<br>• Unclear reporting of age of patients under 70 and control	++
31	Kalsi et al 2015<br>Observational: Prospective cohort comparison	++	• No upgrading or downgrading	++
32	Kornblith et al 2006<br>RCT (secondary analysis of older adults)	++++	• Unclear details on how patients were randomized and if blinded<br>• Attrition of patients<br>• May not be generalizable – differences in baseline levels of depression for completers	++
33	Lapid et al 2007<br>RCT (secondary analysis of older adults)	++++	• Single centre and small sample size<br>• Different forms of cancer, different treatment regimes<br>• Homogeneity (race and religion and location) results may not be generalisable	++
34	Mandelblatt et al 1993<br>Quasi-experimental before and after study	+++	• Women in intervention sig. more hospital visits at both baseline and post periods<br>• Limited generalizability beyond this specific population<br>• Difficult to account for confounding factors or specific effect	++
35	Mantovani et al 1996<br>RCT	++++	• Details of randomization process not included<br>• Unclear who gave structured psychotherapy<br>• Small sample size	++
36	McCorkle et al 2000<br>RCT	++++	• No upgrading or downgrading	++++
37	Rao et al 2005<br>RCT (secondary analysis of cancer patients)	++++	• The clinicians who provided geriatric evaluation and management or usual care knew the patients were enrolled in the study.<br>• Under 100 participants and small sub-groups of analysis within this	+++
38	Sajid et al 2016<br>RCT (pilot)	++++	• Pilot study<br>• Process of randomization to groups is not detailed<br>• Very small sample and dropouts high: statistical power limitations	++
39	Somana-Ehrminger et al 2015<br>Observational (population study)	++	• Only 40 patients had consultation compared with 166 who did not: small sample<br>• Comorbidities might have been underestimated due to assessment bias<br>• Not possible to compare CGA Data between groups	+
40	Suh et al 2013<br>RCT	++++	• Low recruitment rate<br>• Selection bias in sampling<br>• One site and homogeneous sample: generalizability questionable	+++
41	van Weert et al 2011<br>RCT (cluster)	++++	• No upgrading or downgrading	++++
42	Yagli and Ulger 2015<br>Quasi experimental Controlled before and after study	+++	• Small sample and single site<br>• Lack of detail in paper on statistical analysis<br>• Lack of generalizability	++
43	Yeom and Heidrich 2013<br>Observational (Cross sectional from an RCT)	++	• No upgrading or downgrading	++

Only two interventions were directly targeted at improving the knowledge, attitudes or skills of the workforce delivering cancer care and treatment to older people through training[29 41] and only two studies directly addressed the second objective of the review to assess the salient features of the cancer care workforce: one qualitative study considered the features of the nursing workforce which older patients felt were important in their care[24] and one study looked at the impact of healthcare

For peer review only

professionals communication on participants' views about their symptom management.[43] The remaining studies reported on improving older people's outcomes via interventions involving a change in the workforce. In five interventions new roles were tested: nurse navigator,[20] depression care manager,[25] nurse case manager,[26] telephone support (trained graduates),[32] and social support volunteers.[35] In other studies, support from additional workforce members was provided to patients. Four studies reported on the increased involvement of a geriatrician or a geriatrics team,[26 28 37 39] one reported on the input of a clinical pharmacist,[23] and one study reported on the input of an additional dietician.[21] In two studies, a current staff member had a different function; in one study a nurse provided targeted cancer screening [34] and in another study a physiotherapist designed exercise and yoga programmes.[42] Three interventions used Advanced Practice Nurses – one in a homecare capacity[36] and two in counselling roles.[27 40] In three studies the role of multidisciplinary team members was highlighted.[22 30 33] In some papers, although a named member or members of the workforce were reported to have implemented or carried out the intervention, it was unclear as to the exact nature of their position. This was the case with two studies using exercise physiologists where it could not be determined if they were existing or new staff members.[38],[40] Only seven studies referred to an explicit theoretical framework or model in intervention design.[20 25 27 29 40 41 43]

The remainder of the analysis reviews the effectiveness of interventions within the framework constructed around the particular problems (related to older people with cancer) that the respective interventions were addressing and, subsequently, ways in which workforce requirements were being adapted to meet needs and improve outcomes (Tables 3-7).

Regular and timely access to care and treatment

Four studies focused on interventions targeted at the problem of systemic delays or inequitable access to treatment in the cancer journey for older people (Table 3). They provide some promising evidence that providing additional support to some groups of older cancer patients can help them navigate the system and access treatment thereby improving the speed and efficacy of care. However, three of these papers provide only low quality evidence.

A high quality RCT reported that older women with breast cancer in the care of a nurse case manager acting as an educator, counsellor and coordinator were significantly more likely to see a radiation oncologist as part of initial evaluation, and to receive breast-conserving surgery and radiation therapy.[26] Further, the difference in receipt of appropriate treatment between women with characteristics associated with lower rates of appropriate treatment (75+, being unmarried, living alone, and being a member of an ethnic minority group) and their respective comparison groups were diminished or eliminated in the intervention group. An observational study reported

For peer review only

that a breast cancer nurse navigator providing support and coordination of patient care from diagnosis until entry into survivorship clinic significantly shortened time to consultation for patients aged 61+ years.[20] A nurse practitioner role was used in a quasi-experimental study to improve screening rates for older Black women of low socioeconomic status by offering screening during a routine visit.[34] Nurse practitioner follow-up screening rates were significantly higher than baseline, compared with control group follow-up rates. A further study assessed the impact of a geriatrician consultation and treatment plan through an analysis of registry data of older breast cancer patients.[39] Patients who had a consultation had more co-morbidities and more advanced and aggressive tumours, were more likely to receive mastectomy and adjuvant therapy, and were less likely to be treated by breast-conserving surgery and adjuvant therapy.

Complications and specific problems from cancer treatment

Four studies reported the use of workforce members with specialist skills to address cancer treatment complications and impact on mortality and survival (Table 4). None of the three low quality studies found any intervention effect on mortality rates, but the one high quality RCT found that specialised home care advanced practice nurses (used to enhance surgical recovery) increased two year survival for late stage cancer patients in the intervention group.[36]

Other lower quality studies in this group included evaluations of face-to-face counselling to address nutritional intake for patients treated with chemotherapy and at risk of malnutrition,[21] an intervention focused on the prevention of post-operative delirium with input from a geriatric team,[28] and comprehensive geriatric assessment (CGA) targeted at chemotherapy tolerance and toxicity.[31] The observational study evaluating CGA for older chemotherapy patients found that CGA patients were more likely to complete cancer treatment as planned but no significant differences were found in relation to mortality or other outcomes measures in relation to the interventions in any of these three studies.

Table 3 Regular and timely access to care and treatment

Key: I= Intervention T= Timepoint C= Control group W: Workforce involved

Source	Setting and older adults sample	Intervention and workforce	Primary Outcomes	Secondary outcomes	Results
Basu et al 2013 [20]	Women age 61+ with breast cancer stage 0-III n=86	I: Patient Navigation: Support and coordination of patient care T: From point	Time diagnosis to oncology appointment		• Significant: intervention as predictor of time to consultation adjusting for clinical and demographic factors (P=.0002) • In this adjusted model, un-

For peer review only

	One cancer centre, USA<br><br>(Other participants reported in same study: n=90 younger patients)	of diagnosis to survivorship clinic<br>C: 'pre' (i.e. no) navigation<br>W: Breast Cancer Nurse (new role)			standardised beta co-efficient was -4.9 indicating time to consultation decreased by almost 5 days
Goodwin *et al.* 2003[26]	Women age 65+ newly diagnosed with breast cancer, n=335<br><br>13 community and two public hospitals, USA	I: Case management: nurse as educator, counsellor, advocate, and coordinator of care for the patient<br>T: 12 months service<br>C: usual care [unclear]<br>W: Nurse case manager (registered nurse trained for this study)	Treatment received in 6 months after breast cancer diagnosis	Patient satisfaction<br><br>Arm function	• Significant: more intervention women saw radiation oncologist in initial evaluation (36% vs 19.3%) (p=.006), received more breast-conserving surgery (28.6% vs 18.7%; P=.031) and radiation therapy (36.0% vs 19.0%; P=.003)<br>• Non-significant: intervention groups more breast reconstruction surgery (9.3% vs 2.6%, P=.054); and said had a real choice in their treatment (82.2% vs 69.9%, P=.020)<br>• No differences in the percentage of women who saw an oncologist, discussed breast reconstruction, underwent complete surgical staging, or had tissue sent for hormone receptor assay
Mandelblatt *et al.* 1993[34]	Women age 65+ screening for breast or cervical cancer n=673<br><br>Two public hospitals (primary care sites), USA	I: Breast and cervical screening intervention during patients' routine visits<br>T: At scheduled appointments<br>C: Physician reminder system used<br>W: Nurse practitioner (extended part of role)	Annual screening rates (Pap tests and mammographies)		• Significant difference between intervention site where annual rate of Pap tests increased to 56.9% from the baseline of 17.8%, and mammographies increased to 40% from 18.3% compared to control site, 18.2% of women receiving Pap tests from a baseline of 11.8% and remained at 18% for mammography (p = 0.01)<br>• Non-significant: At baseline, screening in both groups decreased with increasing patient age. At post-intervention this was no longer case at intervention site.
Somana-Ehrminger *et al.* 2015[39]	Women age 75+ with breast cancer n=206<br><br>Breast and Gynaecological Cancer Registry, France	I: Geriatrician referral and treatment plan<br>C: Patients who did not have a geriatric oncology consultation<br>W:Geriatrician, detician, psychologist, physical therapist or social worker	Independent impact of GOC		• Significant: GOC patients more likely to receive mastectomy and adjuvant therapy (P < 0.0001); and less likely to be treated by breast-conserving surgery and adjuvant therapy (P = 0.003).<br>• 36 of the 40 patients consulted a geriatrician about oncological treatment, and 27 of these 36 patients received treatment suggested by the geriatrician.

Table 4 Complications and specific problems from cancer treatment

Source	Setting and older adults sample	Intervention and workforce	Primary Outcomes	Secondary outcomes	Results

For peer review only

Bourdel-Marchasson *et al.* 2013[21]	Adults age 70+ treated with chemotherapy n=336<br><br>12 public and private settings, France	I: Face to face dietary counselling (Usual care + Nutritional Intervention)<br>T: 6 visits (3-6 months)<br>C: Usual care (no restrictions for dietary advice, oral supplements or prescription of artificial nutrition)<br>W: Dietician. Additional staff member. Trained.	One year mortality	Chemo-therapy management<br><br>Hospitalisation for reasons other than chemotherapy<br><br>Two year mortality	• Significant difference in dietary intake in intervention group (difference of 178kcal/day, p<0.01)<br>• No difference: one year mortality similar in both groups. Usual care group, one year mortality (41.3%, n=69) and Intervention group (43.8% n=74).<br>• No sig difference: on any other outcomes
Hempenius et 2013[28]	Adults age 65+ frail, elective surgery for solid tumour n=260<br><br>University Medical Centre in a large teaching hospital and a community hospital, Netherlands	I: Delirium prevention through assessment and monitoring with resultant individual treatment plan<br>T: During hospital stay<br>C: Usual care. Additional geriatric care provided at the request of the treating physician<br>W: Geriatric team supervised by a geriatrician. Daily assessment by a geriatric nurse.	Incidence of delirium up to 10 days postoperatively	Severity of delirium<br><br>Length of hospital stay<br><br>Complications<br><br>Mortality<br><br>Care dependency<br><br>Quality of life	• Significant difference: return to preoperative living situation between intervention and usual-care groups (67.3% vs.79.1%, OR: 1.84, 95% CI: 1.01-3.37)<br>• No significant difference: between incidence of delirium (9.4% vs. 14.3%, OR: 0.63, 95% CI: 0.29–1.35)<br>• No sig difference: on any other outcomes
Kalsi *et al.* 2015[31]	Adults age 70+ with cancer at recruited start of chemotherapy n=135<br><br>One hospital providing cancer care, London	I: Geriatrician CGA for identified need. Intervention plans made as a result of assessments for modifiable conditions<br>T: pre chemotherapy and further support as needed<br>C: Standard oncology care (CGA responses not shared with oncology team)<br>W: Geriatrician: existing role but additional involvement for high risk patients	CGA impact on chemotherapy tolerance and toxicity<br><br>Rate of planned completion of cancer	Treatment modifications<br><br>Early treatment discontinuation<br><br>Death at six months	• Significant: Intervention more likely to complete cancer treatment as planned (33.8% vs 11.4% (odds ratio (OR) 4.14 (95% CI: 1.50–11.42), P = 0.006) and fewer intervention participants required treatment modifications 43.1% vs 68.6%, (OR 0.34 (95% CI: 0.16–0.73), P=0.006)<br>• Non-significant: lower grade 3 + toxicity rate in the intervention (43.8% vs 52.9%, P = 0.292)<br>• No differences: all-cause death rates at 6 months (20.0% control, 15.4% intervention, P = 0.483)
McCorkle *et al.* 2000[36]	Adults age 60+ post-surgical cancer patients n=375<br><br>Comprehensive cancer centre, USA	I: Specialized home care APNs assess and monitor physical, emotional, and functional status of patients, provide direct care when needed, assist in obtaining services and other resources from the community, and provide teaching, counseling and support during a period of recovery.	Length of survival	Depressive symptoms<br><br>Symptom distress<br><br>Functional status	• Significant: Late stage patients, improved 2 yr survival in the intervention group: 66.7% v 39.6% in control (P < .05) but no difference: in early stage patients<br>• Risk of death approx. doubled in usual care group (adjusted hazard ratio 2.04; CI, 1.33-3.12; P= .001) compared with those in intervention group after adjusting for stage of disease and LOS.<br>• No differences between groups on depressive symptoms, symptom distress or social

For peer review only

		T: 4 weeks with three home visits and five telephone contacts<br>C: Usual follow up care in an ambulatory setting and routine follow up in outpatient clinics upon discharge<br>W: Advanced Practice Nurses (APNs)			dependency over time.

For peer review only

Co-morbidities and complex health needs

The five studies reported here target the health issues that may accompany a cancer diagnosis, but also broader health problems that may not directly relate to the cancer (Table 5). They highlight the importance of recognising and addressing these needs, although the range of outcomes and the variable quality of evidence (three studies of medium quality; two were low quality) make it difficult to draw firm conclusions about the best use of workforce support in this sizeable area.

A cluster RCT evaluating a hospice staff training programme on improving pain assessment and management did not find significant practice improvements or decreases in patient pain severity associated with the intervention.[29] In a different study, a secondary analysis of RCT data on the impact of a depression care manager providing education and support for older patients with depression found that intervention patients with a cancer diagnoses were twice as likely to experience a depression treatment response at 12 months compared to usual care.[25] Rao *et al.* also reviewed the outcomes for cancer patients from a wider RCT evaluating the impact of involving a geriatric team in the care of inpatient and outpatients diagnosed with frailty.[37] The inpatient intervention group showed significant improvements in bodily pain and mental health versus the usual inpatient care group but there was no impact on survival rates. There were no intervention effects on outpatients. An uncontrolled before and after study, reported that using a clinical pharmacist to identify patients' potentially inappropriate medications (PIMs) reduced the number of PIMs at discharge versus admission.[23] A low quality RCT reported that intensified primary healthcare support significantly reduced the number of days in hospital for an intervention group of advanced cancer patients compared to patients receiving standard care.[30]

Quality of life, physical and psychological functioning

Eight studies focused on addressing quality of life (QOL) across its physical and psychological aspects (Table 6). This group of interventions used a range of workforce members (often in therapeutic or

supportive roles) from physiotherapists to advanced practice nurses to trained voluntary input, to address a range of factors underpinning quality of life. They showed mixed evidence of effectiveness. Seven of the studies in this group provided low quality evidence.

Three studies focused on physical functioning in particular. In an RCT with low recruitment rate and possible selection bias, exercise physicians provided Qi exercise training.[40] Both usual care and intervention participants increased their activity levels but the extent of the increase was significantly greater in the intervention group. The intervention also used advanced practice nurses delivering face-to-face counselling and significant improvements in symptom experience, self-efficacy and self-esteem were reported. A controlled before and after study compared the effect of yoga classes (with the input of a physiotherapist/yoga teacher) with a standard exercise programme.[42]

Table 5 Co-morbidities and complex health needs

Source	Setting and older adults sample	Intervention and workforce	Primary Outcomes	Secondary outcomes	Results
Deliens *et al.* 2016[23]	Adults age 70+ with cancer (non-haematological) hospitalised n=91<br><br>Geriatric oncology unit, tertiary hospital, Belgium	I: Medication review: Identification of PIMS and drug interactions<br>T: From point of admission and during hospitalisation<br>C: Before to after<br>W: Clinical Pharmacist (already team member)	Potentially Inappropriate Medications (PIMS)	Drug to drug interactions	• Non–significant: START criteria: 41 PIMS for 31 patients (34%) on hospital admission compared to 7 PIMS for 6 persons (7%) at discharge.<br>• Non-significant: STOPP criteria: 50 PIMs for 29 patients (32%) at admission compared to 16 PIMS for 14 persons (16%) at discharge.
Fann, Fan and Unutzer 2009[25]	Adults age 60+ ICD-9 diagnosis of non-skin cancer and current major depression or dysthymia n=215<br><br>18 primary care clinics at 8 diverse health care organisations, USA	I: Depression management: education, 'behavioural activation', treatment support.<br>T: Up to 12 months. Followed up usual care 12 months more.<br>C: Usual care: received routinely available depression treatment<br>W: Depression care manager (nurse or clinical psychologist) collaborative with primary care. Trained and supervised by psychiatrist.	Depression treatment response	Health related quality of life<br><br>Health-related impairments: work, family, social functioning<br><br>Patient satisfaction	• Significant: Intervention twice as likely to experience a depression treatment response at 12 months than control (39% vs. 20%; P = 0.029) and at 18 months (38% vs. 16%; P = 0.012)<br>• Significant: Remission rates higher in intervention group v control at 6 months (32% v 16% P = 0.006) and 12 months (22% v 9%, P = 0.031).<br>• Significant: Less functional impairment at 12 months (P=0.011) and greater QOL (P=0.039)
Herr *et al.* 2012[29]	Adults age 65+ with cancer receiving hospice care n=738<br><br>Staff: Nurses (n=383 pre,	I: Workforce: to promote adoption of evidence-based pain practices. Included: training, assessment of data, champion input, senior leadership	Workforce: adoption of evidence-based (EB) cancer pain practices	Pain severity	• No significant difference in improvement on Cancer Pain Practice Index between intervention and control sites<br>• Non-significant: Decrease in patient pain severity from pre to post in intervention group greater (p=0.1032)

	n=415 post) and physicians (n=16)<br><br>16 hospices, USA	engagement.<br>T: Engagement phase 5 months, 12 month intervention.<br>C: Hospices received clinical practice guidelines<br>W: 3 days training. Selection of local pain facilitators, nurse and physician champions, Grant Expert Nurse input, Nurse and Physician Champion			
Johansson *et al.* 2001[30]	Adults age 70+ newly diagnosed with prostate, GI or breast cancer n=161<br><br>Primary healthcare services, Sweden<br><br>(Other participants reported: n=255 under 70yrs)	I: Intensified primary healthcare. Individual support: nurse support, nutritional support, and individual psychological support.<br>T: Starting from diagnosis<br>C: Standard care + group rehabilitation<br>W: Homecare nurse, dietician and psychologist. GPs and nurses trained in pain, nausea and diet, in final stage life.	Utilisation of specialist care		• Significant: Mean days of hospitalization for older intervention patients lower than older control group patients (3.8 v 8.9, P<0.01)<br>• Only 4 of 82 older intervention patients had utilized acute admissions compared with 12 of 79 among the older control patients (P<0.05)<br>• 10 out of 82 made acute visits to outpatient clinics compared to 22 of 79 in control group (P<0.05)
Rao *et al.* 2005[37]	Adults age 65+ with cancer, frail and hospitalized n=99<br><br>11 Department of Veterans Affairs Medical Centers, USA	I: Assessment and monitoring by geriatric team. 1. geriatric inpatient +usual outpatient; 2. Usual inpatient +geriatric outpatient; 3. Geriatric inpatient and outpatient<br>T: 1 year study<br>C: Usual care: all hospital services except from geriatric team<br>W: Core team: geriatric medicine attending physician, fellow or intern, a nurse practitioner, social worker	Survival<br><br>Health related quality of life	Functional status<br><br>Physical performance	• No difference: in survival for the cancer patients regardless of treatment group<br>• Significant effect: of geriatric inpatient care v usual inpatient care mean change in score from randomization to discharge: bodily pain (28.7 v 10.1) P=0.09; emotional limitation (29.3 v 2.7) P=.01. Effect on bodily pain sustained at one year (37.6 v 9.9)<br>• No effect of geriatric outpatient care on any of the QOL parameters<br>• No effect of either inpatient or outpatient geriatric care on the functional status of cancer patients

Table 6 Quality of Life, physical and psychological functioning

Source	Setting and older adults sample	Intervention and workforce	Primary Outcomes	Secondary outcomes	Results
Chock *et al.* 2013[22] (Secondary analysis of Clark et al. 2013)[44]	Adults age 65+ with advanced cancer treated with radiotherapy n=16<br><br>Cancer centre, USA<br><br>(Other	I: QOL intervention with telephone follow up: Physical therapy, education, cognitive behavioural interventions, discussion and	Quality of Life		• Significant difference at week 4 only in mean overall QOL older v younger adults (LASA scale 74.4 vs 62.9, p=0.040)<br>• No sig. difference at weeks 27 and 52<br>• No sig. difference in overall Profile of Mood States (POMS) scores between older and younger groups

For peer review only

	participants reported: n=38 younger adults)	support, spiritual reflection and relaxation training<br>T: 6 sessions 90 mins, 2-4 weeks and 10 brief structured telephone sessions<br>C: Standard care<br>W: Multidisciplinary (including physical therapist, clinical psychologist, APN, Chaplain)			• Significantly lower POMS Anger-Hostility dimension at all weeks except baseline. Week 4: 95.0 vs 86.4, p=0.028; wk 27: 92.2 v 84.2, p=0.027) and wk 52 (96.3 v 85.9, p=0.005)
Heidrich *et al.* 2009[27]	Women age 65+, 1 year post diagnosis of non-metastatic breast cancer n=82 (total)<br><br>Oncology clinics of a large comprehensive cancer centre, USA	I: Pilot 1 - Symptom management (IRIS): counselling interview and telephone follow up on symptom management at 4 weeks; Pilot 2- Addition of four biweekly telephone reinforcement sessions; Pilot 3 - Intervention by phone only<br>C: 1 -usual care; 2-delayed IRIS (waitlist) control; 3 - no control: (IRIS Group only)<br>T: 4 weeks (pilot 1)<br>W: Advanced practice Nurses	Feasibility, acceptability	Symptom distress<br><br>Symptom management<br><br>Quality of Life<br><br>Mood<br><br>Barriers to symptom management<br><br>**+Pilot 3:** Communication difficulty	• Feasibility: Across all studies, 76% of eligible women participated, 95% completed the study, 88% reported the study was helpful, and 91% were satisfied with the study.<br>**Pilot 1**<br>• Non-sig. difference between Intervention and control in symptom distress. Significant decrease in distress baseline to follow up in intervention group; Significantly more women in intervention reported changing self-care of symptoms (p<0.05); No significant differences on QOL<br>**Pilot 2**<br>• Significant less symptom duration compared to the control at 8 weeks (p <0.01). At 16 weeks, Int. group sig. more likely to have talked to healthcare provider, begun new symptom treatment, and changed self-care symptoms (p<0.05). No sig differences in QOL. Negative attitudes from healthcare providers reported by 5%–20% of women and communication difficulties by 5%–45% of women.<br>**Pilot 3**<br>• Non-significant differences (no control) baseline to eight weeks, symptom interference decreased (and negative mood from symptoms). Symptom duration interference, and negative mood from symptoms decreased. No change in QOL.
Kornblith *et al.* 2006[32]	Adults age 65+ with breast cancer stage III or IV; Duke stage C or D colon cancer or stage C or D prostate cancer n=131<br><br>Range of cancer centres/university settings, USA	I: Telephone monitoring of distress providing support (plus educational materials)<br>T: Over 6 months – monthly monitoring<br>C: Educational materials alone, referred to oncology nurse	Psychological distress		• Significant: lower anxiety and depression mean HADS total score for intervention 6.01 v 8.20 control (p<.0001); HADS Depression subscale, Intervention 3.20 v 4.08 control (p=.0004); HADS Anxiety subscale Intervention 2.81 v 3.25 control (p<.0001), at 6 months controlling for study entry levels.<br>• No differences on other measures of psychological distress

For peer review only

		upon evaluation if distressed significantly<br>W: Trained graduates monitoring telephone calls. Referral on to an oncology nurse where indicated			
Lapid *et al.* 2007[33]<br><br>Secondary age group analysis of Rummans et al. 2006[45]	Adults age 65+ newly diagnosed with advanced cancer<br>n=33<br><br>Cancer Centre, USA	I: Multidisciplinary psychosocial QOL sessions<br>T: Eight 90 min sessions, four weeks after enrollment<br>C: Standard care (regular outpatient visits with oncologist and allied health care providers)<br>W: Led by psychiatrist or psychologist and co-facilitated by a nurse, physical therapist, chaplain or social worker. Leaders trained in materials and observed sessions.	Quality of Life		• Non-significant: Higher overall QOL intervention group scores throughout the study<br>• Significant: higher QOL scores at week 4 intervention v control (79.3 vs 62.9, p=.0461)<br>• Improvement in QOL scores for intervention at week 4 and 8 compared to older control group
Mantovani *et al.* 1996[35]	Adults age 65+ with cancer<br>n=72<br>Inpatient setting at University medical oncology clinic, Italy	I: 1) Emotional and practical support from volunteers and 2) with structured psychotherapy<br>T: 2)Weekly sessions of 1 hour for 6 months<br>C: Pharmacological only<br>W: Trained volunteers basic = 40hrs/6 months, another 40 hrs/6 months practical and further personal training.	Quality of Life		• Non-significant between group differences on quality of life measures : Karnofsky's Performance Status Scale, Scott's Huskisson's visual analogue for pain (p<0.001)<br>• No differences on Spitzer's Quality of Life Index or functional Living Index -Cancer questionnaire within/between groups<br>• Significant between group differences: Stait-Trait Anxiety Inventory X1-form control significantly worsened and intervention groups sig. improved<br>• Beck Depression Inventory: control group unchanged, both intervention groups improved
Sajid *et al.* 2016[38]	Men age 70+ with prostate cancer and hormone therapy<br>n=19<br><br>Two University Medical Oncology clinics, USA	I1. EXCAP (home based walking and resistance intervention)<br>I2. Technology mediated walking and resistance intervention using Wi-Fit<br>T: One face to face session then	Functional and aerobic	Skeletal muscle and Muscular Mass Measure<br><br>Handgrip strength<br><br>Chest repetition test<br><br>DEXA scan	• Significant: EXCAP intervention arm higher rate of change in steps per day at each follow up (+2720 steps) (p < 0.01) compared to control (+97 steps) and Wii-fit arm (+382 non- significant.)<br>• EXCAP arm had a 2.3 point change in physical battery score after 12 weeks, compared to 0.6 points in the Wii-Fit arm and −0.5 points in the usual-care arm.<br>• Non-significant differences on

For peer review only

		6-12 weeks home based<br>C: Usual care<br>W: American College of Sports Medicine (ACSM)-trained exercise physiologist designated at each site			other outcomes between groups.
Suh *et al.* 2013[40]	Adults age 65+ completed active treatment for GI cancers<br>n=63<br><br>Cancer Centre, South Korea	I: 8 weeks of Qi exercise and 1 hour face-to-face counseling on physical and psychological factors<br>T: 8 weeks<br>C: Usual care<br>W: Two Qi exercise trainers, Nurse clinicians (APN)	Physical activity	BMI and body weight<br><br>Nutritional status<br><br>Symptom experience<br><br>Self-efficacy<br><br>Self-esteem	• Physical activity increased in both groups, extent of increase significantly greater in the I than in the C ($p = 0.005$) and difference in amount of in amount of exercise sig. over time between groups ($p = 0.002$)<br>• No between group difference in BMI category change<br>• Nutritional status in both groups improved over time. The degree of reduction, however, was significantly larger in the I than the C ($p = 0.048$), and same in interaction between group and time<br>• Both group and interaction factors significant positive difference in symptom experience, health promotion and Self-esteem for intervention
Yagli and Ulger 2015[42]	Women age 65-70 6 months after chemotherapy for breast cancer finished<br>n=20<br><br>Department of Physiotherapy and Rehabilitation, University, Turkey	I: 8 sessions of 1 hr yoga classes<br>T: 8 weeks<br>C: Exercise programme for 8 weeks<br>W: Existing physiotherapist (yoga teacher)	Quality of life<br><br>Depression levels<br><br>Level of pain, fatigue and sleep quality		• All patients' quality of life scores improved pre to post yoga and exercise interventions<br>• Total scores and some sub categories of the Nottingham Health Profile showed significant difference in favor of the yoga group ($p < 0.05$) but not on energy level and pain where no differences<br>• Significant better fatigue and sleep quality in yoga group post intervention ($p < 0.05$)

QOL scores after the program were better than before for both groups, but some QOL parameters improved more for those included in the yoga intervention. A pilot RCT with small sample and high dropout compared two exercise forms implemented by a physiologist (compared to usual care) and found significant activity increases for the group using a home-based walking and resistance intervention.[38]

Two similar interventions involved a multidisciplinary team approach for a range of quality of life domains, however both of these secondary analyses reported on very small samples sizes of older adults within wider QOL interventions. Lapid *et al.*[33] found in a secondary analysis of a small sample of patients in a wider RCT, that higher QOL scores were reported for older patients who received multidisciplinary emotional and practical support. However, in the study by Chock et al,[22] the

For peer review only

authors did not find any lasting differences on QOL for older intervention participants against their younger counterparts, apart from an improvement in anger-hostility.

Advanced practice nurses were used in a symptom management intervention in the two pilot RCTs and the observational study reported by Heidrich *et al.*[27] Some evidence of effectiveness was reported for improving self-care and reducing symptom distress and duration, but there was no impact on QOL.

Two studies used trained volunteers to bolster psychological support. A secondary analysis of RCT data was used to evaluate the effect of using trained graduate support workers to provide initial distress monitoring to patients over the telephone. [32] Intervention patients had significantly lower anxiety and depression at six months than patients receiving educational materials alone. However, no other differences in psychological wellbeing were detected. Mantovani *et al.*[35] also used trained support volunteers to provide emotional and practical support. An RCT with small sample size was used to compare this support with pharmacological treatment alone, and further with the addition of psychotherapy. Significant improvements in anxiety and depression were reported for the groups receiving voluntary support and/or additional psychotherapy. However, there were no significant differences on other QOL measures.

Communication between health care professionals (HCPs) and older people with cancer

Three studies focused on addressing the communication needs of older people with cancer. One high quality study offered communication skills training to staff with varied success[41] and the other two low quality studies highlighted the importance of good communication as a prerequisite for cancer nurses related to improving older patients' quality of life (Table 7).

A cluster RCT found that training nursing staff to improve chemotherapy patient education led to a significant, positive effect for 'discussing realistic expectations'.[41] Significantly less history taking was also observed pre to post in the intervention group, as well as less talking about all the possible side effects; both points of attention during training. No other significant effects were reported. Yeom and Heidrich[43] used a cross-sectional analysis of RCT data to report that communication difficulties with health professionals had significant direct, negative effects on QOL dimensions. Findings from a qualitative interview study highlighted the value to older cancer patients of nurses having a person-centred manner, with the ability to show a genuine and empathic interest in the patients and to make a connection with good listening and communication skills.[24]

For peer review only

Table 7: Communication between health care professionals and older people with cancer

Source	Setting and older adults sample	Intervention and workforce	Primary Outcomes	Secondary outcomes	Results
Devik, Hellzen and Enmarker 2015[24]	Adults age 65+ with advanced cancer n=9<br><br>Patients homes rural locations, Norway	I: Qualitative study of home nursing care to advanced cancer patients in rural locations<br>C: n/a<br>W: District nurses in normal role	(qual) Patient experience		• Importance of nurses having a person-centred manner<br>• Ability to show a genuine and empathic interest in the patients<br>• Technical skills or special competences less discussed than personal qualities, such as having a sense of humour or generosity<br>• Good listening and communication skills
van Weert *et al.* 2011[41]	Adults 65+with cancer receiving chemotherapy<br><br>n=210<br><br>Staff: oncology trained nurses n=77<br><br>12 wards of 10 hospitals, Netherlands	I: Workforce: communication skills training in delivery of chemotherapy education to patients<br>T: 3 month implementation<br>C: Nurses continued to provide patient education as usual<br>W: Nursing and specialised oncology nursing roles. Training provided.	Workforce based outcome:<br><br>Effects on quality of communication<br><br>Effects on content of the consultation	Patient based outcome: recall of information	• Significant improvement in category discussing realistic expectations pre to post. C: -0.20; I: 0.45 (total between group difference 0.65) p<.01<br>• Significant decrease in rehabilitation information pre to post change C:0.08, I: -0.38 (total between group difference -0.45) p<.01<br>• No significant changes in categories treatment-related information and coping information.<br>• Non sig: intervention group showed significant decrease in number of items discussed<br>• Less history taking pre to post (C: 1.83; I: -2.33; between group difference -4.17; p<.001) and less talking about all different side effects pre to post change (C: 1.98; I: -5.71; total 7.68; p<.001).<br>• Patients in intervention asked more questions (M= 10.76) than control (M= 6.69; p <.05).<br>• Marginal significant for intervention group: Proportion recall of recommendations post v pre. (C: -3.34; I: 6.39; Total: 9.73; p <.10)
Yeom and Heidrich 2013[43]	190 women at least one year post breast cancer diagnosis n=190<br><br>Community, an oncology clinic, and a state tumor registry, USA	I: Symptom management (IRIS): counselling interview and telephone follow up on symptom management<br>T: 8 week intervention with 16 week follow up point in the RCT.<br>C: Wait-list control subjects offered intervention after 16-week follow-up assessment	Negative beliefs about symptom management	Quality of lIfe<br><br>Purpose in life<br><br>Positive relations with others	• Significant direct effects of Symptom Management Beliefs Questionnaire (SMBQ) (p<0.00) and Communication Attitudes Questionnaire (p = 0.012) on Communication Difficulties Questionnaire<br>• Communication Difficulties significant direct, negative effects on all four dimensions of quality of life<br>• Significant total effects of SMBQ on MCS (mental quality of life) (p=0.001) and Purpose on Life (PL) and Personal Relations (PR) (p<0.001) but not Physical component (PCS) .SMBQ predicted lower levels of quality of life in three of four dimensions.

		W: Advanced practice Nurses			• None of the four indirect effects of SMBQ on quality of life through CommD was significant, indicating that CommD does not mediate the effects of SMBQ on quality of life<br>• The total effects of CommA on four quality of life measures were not significant. However, the indirect effects for MCS (p=0.05), PIL (p <0.05) and PR (p < 0.05) through CommD were significant, indicating that CommD mediates the effects of CommA on MCS, PIL and PR.

For peer review only

DISCUSSION

This systematic review aimed to provide an overview of the evidence base on the effectiveness of workforce interventions for improving the outcomes for older people with cancer, as well as analysing key features of the workforce associated with those improvements. Findings reflect a range of ways in which the workforce has been adapted, expanded or trained to addressing older cancer patients multiple and divergent needs. The findings present a novel synthesis of the type of interventions being developed globally to address the broad question of how the workforce can support the improvement of older people's cancer outcomes. The approaches in themselves are varied, including integrating the input of geriatric specialists into cancer services, using advanced practice nurse roles to support patients, creating new roles to guide patients through the healthcare system and ensuring effective treatment, through to novel approaches using voluntary support, or trialling yoga or other exercise to improve older patients' quality of life.

While the included studies begin to provide evidence about how the workforce can be used to make a tangible difference to older cancer patients' physical and psychological outcomes, the diversity of interventions in the studies reviewed and the range of outcomes evaluated, limits generalisations on effectiveness. Further, the quality of evidence is generally low. Experimental designs were not consistently used and, when they were, their implementation was often hampered by poorer than expected recruitment, or conclusions drawn about outcomes for older patients were drawn from a secondary analysis of a wider dataset. In addition, as is common in the reporting of complex intervention evaluations, details of the intervention itself were often lacking.[46] There was inadequate reporting of the specific workforce contribution to the interventions and limited evidence to address the second objective of the review around the features of the cancer care workforce associated with better outcomes. In addition, while staff training was involved in half of the interventions reported, the details of how that training worked or could be improved was not

detailed. Furthermore, although some innovative roles were set up, the rationale and detail of those roles was often poorly reported.

Despite these shortcomings, these findings do provide some promising insights into how the workforce may address the varied needs of older cancer patients, albeit with a dearth of evidence at the earlier and later stages of the cancer journey. Evidence has suggested that not all older people with cancer need the same input, and indeed age-related changes occur at different rates in different individuals and are not reflected in chronologic age.[7] Therefore, it is more productive to focus attention on those with complex problems.[47] The studies in this review appear to support the notion of targeted assistance to groups at particular risk of under-treatment. Review findings suggest that broader interventions aiming to improve survival outcomes are less successful, but studies did indicate the kind of support that could be put in place after treatment to deal with the specific complications and problems that older people might face. One intervention which did improve survival used advanced practice nurses in home-care support post-surgery.[36] Indeed, the role of advanced practice nurses in the future of older people's cancer care has been acknowledged elsewhere in the literature,[48-51] and this review indicates that this is a candidate role for exploration and further consideration.

The input of geriatric specialists who are able to assess and manage older patients and optimise patients for treatment was a significant feature of several studies reviewed and formal links and services are well established in some countries.[52-54] Findings from this review provide weak evidence of positive benefits from the input of geriatricians but it only included studies where the geriatrician's role was explicit in the intervention and where a comparison or control was featured. There are a number of other reviews reporting on specialist geriatric assessment and management for older cancer patients, and these have been able to draw firmer conclusions about the benefits of CGA with older cancer patients, although they all acknowledge the need for more definitive research.[55-57] Multidisciplinary approaches also emerged as a feature across the studies reviewed and the need to shape teams around the multiple needs of older people with cancer has been highlighted elsewhere, although evidence from this review is weak, again limited by the scale and quality of the research.[6 58-61]

Of further interest is the use of non-professionals in providing direct care services to older people with cancer, and roles such as these are relevant in the contexts of budgetary pressures and recruitment difficulties of key professional groups such as geriatricians and registered nurses.[17] The two studies reviewed suggested a positive impact on patient outcomes and align with a growing recognition of the non-clinical workforce (including carers and families) playing an essential role in

For peer review only - http://bmjopen.bmj.com/site/about/guidelines.xhtml

older people's cancer care.[62-64] However, the low quality of the research again reduces confidence in these positive findings. A final point is that the studies identified for this review did not address the impact of staffing levels or skillmix on older cancer patients' outcomes. In addition, few mechanisms to develop the current workforce to prepare for and be supported to deliver high quality care to an ageing population were identified. In addition to the development and more definitive evaluation of new roles and practices, the future research agenda must address these important facets to ensure that, regardless of setting, all health care workers that older people with cancer encounter, are prepared for and adequately supported in their role.[65 66]

This review alone is insufficient to enable conclusions to be drawn about the workforce factors which prove most beneficial to older people's outcomes; further high quality RCTs are needed to assess the potential of possible interventions. Future research should build on the studies reviewed here to establish what workforce developments are needed to support this growing population throughout the cancer journey.

For peer review only

Funding statement

This work was funded by Macmillan Cancer Support to support the work of the Expert Reference Group for the Older Person with Cancer. The views expressed are those of the authors and do not necessarily reflect those of Macmillan Cancer Support.

Contributors: JB, GL, PG and TW were responsible for the systematic review design. GL was responsible for data collection. JB, GL and PG were responsible for data extraction and appraising studies. JB, GL, PG and TW were responsible for data analysis and interpretation. All authors contributed in drafting the manuscript. JB is responsible for the overall content as the corresponding author.

None of the named authors has a conflict of interest in relation to this work or its publication.

Data sharing statement No additional unpublished data are available.

Reporting Guidelines: Prisma checklist (supplementary file 3)

For peer review only

REFERENCES

1. Yancik R, Ries LAG. Cancer in older persons: an international issue in an aging world. *Semin Oncol*;**31**(2):128-36. doi: 10.1053/j.seminoncol.2003.12.024

2. Coleman MP, Forman D, Bryant H, et al. Cancer survival in Australia, Canada, Denmark, Norway, Sweden, and the UK, 1995–2007 (the International Cancer Benchmarking Partnership): an analysis of population-based cancer registry data. *Lancet*;**377**(9760):127-38. doi: 10.1016/S0140-6736(10)62231-3. [published online first 21 December 2010]

3. International Society of Geriatric Oncology. SIOG 10 Priorities Initiative. 2011. http://www.siog.org/content/siog-10-priorities-initiative (accessed January 2017)

4. Lynch MP, DeDonato DM, Kutney-Lee A. Geriatric Oncology Program Development and Gero-Oncology Nursing. *Semin Oncol Nurs* 2016;**32**(1):44-54. doi: 10.1016/j.soncn.2015.11.006 [published online first: 17 November 2015]

5. Prouse J, Phillips J. Care of older people living with cancer: The role of the specialist nurse and alied health profesionals. *Cancer Forum* 2013;**37**(3):226-29. https://opus.lib.uts.edu.au/handle/10453/29024 (accessed 24 Jan 2017)

6. Bridges J, Hughes J, Farrington N, et al. Cancer treatment decision-making processes for older patients with complex needs: a qualitative study. *BMJ Open* 2015;**5**(12):e009674. doi: 10.1136/bmjopen-2015-009674

7. Balducci L, Extermann M. Management of cancer in the older person: a practical approach. *Oncologist* 2000;**5**(3):224-37. doi: 10.1634/theoncologist.5-3-224

8. Caillet P, Laurent M, Bastuji-Garin S, et al. Optimal management of elderly cancer patients: usefulness of the Comprehensive Geriatric Assessment. *Clin Interv Aging*2014;**9**:1645-60. doi: 10.2147/CIA.S57849

9. Expert Reference Group for the Older Person with Cancer. Older people living with cancer: designing the future health care workforce, Macmillan Cancer Support 2016. http://www.macmillan.org.uk/assets/workforce-report.pdf (accessed January 2017)

10. Bridges J, Wengström Y, Bailey DE. Educational preparation of nurses caring for older people with cancer: an international perspective. *Semin Oncol Nurs* 2015;**32**(1):16-23. doi: 10.1016/j.soncn.2015.11.003 [published online first: 17 November 2015]

11. Independent Cancer Taskforce. Achieving world-class cancer outcomes. A strategy for England 2015-2020. https://www.cancerresearchuk.org/sites/default/files/achieving_world-class_cancer_outcomes_-_a_strategy_for_england_2015-2020.pdf (Accessed January 2017).

12. Lyman GH. Caring for the elderly cancer patient: training the next generation of oncologists. *J Oncol Pract*2008;**4**(4):193-94. doi: 10.1200/JOP.0841503

13. Rao AV, Hurria A, Kimmick G, et al. Geriatric Oncology: Past, Present, Future. *J Oncol Pract* 2008;**4**(4):190-92. doi: 10.1200/JOP.0846001

14. Maggiore RJ, Gorawara-Bhat R, Levine SK, et al. Perceptions, attitudes, and experiences of hematology/oncology fellows toward incorporating geriatrics in their training. *J Geriatr Oncol* 2014;**5**(1):106-15. doi: 10.1016/j.jgo.2013.10.003 [published online first: 7 Nov 2013].

15. Foubert J, Faithfull S. Education in Europe: Are cancer nurses ready for the future? *JBUON* 2006;**11**(3):281-84.

16. McMillan S, Grodzicki BK, Shahrokni A. Building a Comprehensive Educational Model for Nurse Practitioner Students in Geriatric Oncology[abstract]. *J Am Geriatr Soc* 2016;**64**:(Suppl)180-S80.

17. Bridges J, Lucas G, Wiseman T, et al. Preparedness of UK workforce to deliver cancer care to older people: summary report from a scoping review. 2016. http://eprints.soton.ac.uk/401192/ (Accessed January 2017).

18. Higgins JG, Green S (editors). *Cochrane Handbook for Systematic Reviews of Interventions Version 5.1.0*. The Cochrane Collection 2011. http://handbook.cochrane.org

19. Moher D, Liberati A, Tetzlaff J, et al. Preferred Reporting Items for Systematic Reviews and Meta-Analyses: The PRISMA Statement. *PLoS Med* 2009;**6**(7):e1000097. doi:10.1371/journal.pmed.1000097

For peer review only

20. Basu M, Linebarger J, Gabram SGA, et al. The effect of nurse navigation on timeliness of breast cancer care at an academic comprehensive cancer center. *Cancer* 2013;**119**(14):2524-31. doi: 10.1002/cncr.28024 (published online first: 12 April 2013).

21. Bourdel-Marchasson I, Blanc-Bisson C, Doussau A, et al. Nutritional Advice in Older Patients at Risk of Malnutrition during Treatment for Chemotherapy: A Two-Year Randomized Controlled Trial *PLoS One* 2014; **9**(9):e108687. doi: 10.1371/journal.pone.0108687.

22. Chock MM, Lapid MI, Atherton PJ, et al. Impact of a structured multidisciplinary intervention on quality of life of older adults with advanced cancer. *Int Psychogeriatr* 2013; **25**(12):2077-86. doi: 10.1017/S1041610213001452 [published online first 4 September 2013]

23. Deliens C, Deliens G, Filleul O, et al. Drugs prescribed for patients hospitalized in a geriatric oncology unit: Potentially inappropriate medications and impact of a clinical pharmacist. *J Geriatr Oncol* 2016; **7**(6):463-470. doi: 10.1016/j.jgo.2016.05.001 (published online first: 26 May 2016)

24. Devik SA, Hellzen O, Enmarker I. "Picking up the pieces" - Meanings of receiving home nursing care when being old and living with advanced cancer in a rural area. *Int J Qual Stud Health Well-being* 2015;**10**:28382. doi: 10.3402/qhw.v10.28382

25. Fann JR, Fan MY, Unützer J. Improving primary care for older adults with cancer and depression. *J Gen Intern Med*;**24**(Suppl 2). doi: 10.1007/s11606-009-0999-4

26. Goodwin JS, Satish S, Anderson ET, et al. Effect of nurse case management on the treatment of older women with breast cancer. *J Am Geriatr Soc* 2003; **51(**9):1252-9.

27. Heidrich SM, Brown RL, Egan JJ, et al. An individualized representational intervention to improve symptom management (IRIS) in older breast cancer survivors: three pilot studies. *Oncol Nurs Forum* 2009;**36**(3):E133-43. doi: 10.1188/09.ONF.E133-E143

28. Hempenius L, Slaets JPJ, Asselt D, et al. Outcomes of a Geriatric Liaison Intervention to Prevent the Development of Postoperative Delirium in Frail Elderly Cancer Patients: Report on a Multicentre, Randomized, Controlled Trial. *PloS One* 2013;**8**(6):e64834. doi: 10.1371/journal.pone.0064834

29. Herr K, Titler M, Fine PG, et al. The effect of a translating research into practice (TRIP)--cancer intervention on cancer pain management in older adults in hospice. *Pain Med* 2012; **13**(8):1004-17. doi: 10.1111/j.1526-4637.2012.01405.x [published online first: 3 July 2012].

30. Johansson B, Holmberg L, Berglund G, et al. Reduced utilisation of specialist care among elderly cancer patients: A randomised study of a primary care intervention. *Eur J Cancer* 2001; **37**(17):2161-8. doi: 10.1016/S0959-8049(01)00278-7

31. Kalsi T, Babic-Illman G, Ross PJ, et al. The impact of comprehensive geriatric assessment interventions on tolerance to chemotherapy in older people. *Br J Cancer* 2015;**112**(9):1435-44. doi: 10.1038/bjc.2015.120 [published online first 14 April 2015].

32. Kornblith AB, Dowell JM, Herndon JE, et al. Telephone monitoring of distress in patients aged 65 years or older with advanced stage cancer: a cancer and leukemia group B study. *Cancer* 2006; **107**(11): 2706-14. DOI: 10.1002/cncr.22296

33. Lapid MI, Rummans TA, Brown PD, et al. Improving the quality of life of geriatric cancer patients with a structured multidisciplinary intervention: a randomized controlled trial. *Palliat Support Care* 2007; **5**(2):107-14. doi: 10.1017/S1478951507070174

34. Mandelblatt J, Traxler M, Lakin P, et al. A nurse practitioner intervention to increase breast and cervical cancer screening for poor, elderly black women. The Harlem Study Team. *J Gen Intern Med* 1993;**8**(4):173-8.

35. Mantovani G, Astara G, Lampis B, et al. Impact of psychosocial intervention on the quality of life of elderly cancer patients. *Psychooncology* 1996; **5**(2):127-35. doi: 10.1002/(SICI)1099-1611(199606)5:2<127::AID-PON220>3.0.CO;2-L

36. McCorkle R, Strumpf NE, Nuamah IF, et al. A specialized home care intervention improves survival among older post-surgical cancer patients. *J Am Geriatr Soc* 2000;**48**(12):1707-13. doi: 10.1111/j.1532-5415.2000.tb03886.x

For peer review only

37. Rao AV, Hsieh F, Feussner JR. Geriatric evaluation and management units in the care of the frail elderly cancer patient. *J Gerontol A Biol Sci Med Sci* 2005; **60**(6):798-803.

38. Sajid S, Dale W, Mustian K, et al. Novel physical activity interventions for older patients with prostate cancer on hormone therapy: A pilot randomized study. *J Geriatr Oncol* 2016; **7**(2):71-80. doi: 10.1016/j.jgo.2016.02.002 [published online first: 23 February 2016].

39. Somana-Ehrminger S, Dabakuyo TS, Manckoundia P, et al. Influence of geriatric oncology consultation on the management of breast cancer in older women: a French population-based study. *Geriatr Gerontol Int.* 2015;**15**(1):111-9. doi: 10.1111/ggi.12240 (published online first: 24 January 2014).

40. Suh EE, Kim H, Kang J, et al. Outcomes of a culturally responsive health promotion program for elderly Korean survivors of gastrointestinal cancers: a randomized controlled trial. *Geriatr Nurs.* 2013; **34**(6):445-52. doi: 10.1016/j.gerinurse.2013.09.007 [published online first: 1 October 2013].

41. van Weert JC, Jansen J, Spreeuwenberg PM, et al. Effects of communication skills training and a Question Prompt Sheet to improve communication with older cancer patients: a randomized controlled trial. *Crit Rev Oncol Hematol* 2011; **80**(1):145-59. doi: 10.1016/j.critrevonc.2010.10.010 (published online first 13 November 2010).

42. Yagli NV, Ulger O. The effects of yoga on the quality of life and depression in elderly breast cancer patients. *Complement Ther Clin Pract* 2015; **21**(1):7-10. doi: 10.1016/j.ctcp.2015.01.002 [published online first: 29 January 2015].

43. Yeom HE, Heidrich SM. Relationships between three beliefs as barriers to symptom management and quality of life in older breast cancer survivors. *Oncol Nurs Forum* 2013; **40**(3):E108-18. doi: 10.1188/13.ONF.E108-E118

44. Clark MM, Rummans TA, Atherton PJ, et al. Randomized Controlled Trial of Maintaining Quality of Life During Radiation Therapy for Advanced Cancer. *Cancer* 2013;**119**(4):880-87. doi: 10.1002/cncr.27776 (published online first: 28 Aug 2012).

45. Rummans TA, Clark MM, Sloan JA, et al. Impacting quality of life for patients with advanced cancer with a structured multidisciplinary intervention: a randomized controlled trial. *J Clin Oncol* : 2006;**24**(4):635-42. doi: 10.1200/JCO.2006.06.209

46. Craig P, Dieppe P, Macintyre S, et al. Developing and evaluating complex interventions: the new Medical Research Council guidance. *BMJ* 2008;**337**:979-83. doi: 10.1136/bmj.a1655

47. Bridges J, Simcock R. Meeting the workforce challenges for older people living with cancer. *Int J Nurs Stud*;**65**:A1-A2. doi: 10.1016/j.ijnurstu.2016.11.013

48. Kagan SH. The Future of Gero-Oncology Nursing. *Semin Oncol Nurs* 2016;**32**(1):65-76. doi: 10.1016/j.soncn.2015.11.008

49. Levenson D. Advanced practice nurses lengthen survival of elderly cancer patients. *Rep Med Guidel Outcomes Res* 2001;**12**(5):5-7.

50. Morgan B, Tarbi E. The Role of the Advanced Practice Nurse in Geriatric Oncology Care. *Semin Oncol Nurs* 2016;**32**(1):33-43. doi: 10.1016/j.soncn.2015.11.005

51. Prouse J. Geriatric assessment. The role of the specialist nurse practitioner [abstract]. *Asia Pac J Clin Oncol* 2009;**5** (suppl. 2):A155.

52. Extermann M, Aapro M, Audisio R, et al. Main priorities for the development of geriatric oncology: a worldwide expert perspective. *J Geriatr Oncol* 2011;**2**(4):270-73. doi: 10.1016/j.jgo.2011.07.001

53. Gosney M. Contribution of the geriatrician to the management of cancer in older patients. *Eur J Cancer* 2007;**43**(15):2153-60. doi: 10.1016/j.ejca.2007.07.032

54. Magnuson A, Dale W, Mohile S. Models of Care in Geriatric Oncology. *Curr Geriatr Rep* 2014;**3**(3):182-89. doi: 10.1007/s13670-014-0095-4 [published online first: 8 July 2014].

For peer review only

55. Puts M, Santos B, Hardt J, et al. An update on a systematic review of the use of geriatric assessment for older adults in oncology. *Ann Oncol* 2014;25(2):307-15. doi: 10.1093/annonc/mdt386 [published online first: 19 November 2013].

56. Hamaker ME, Schiphorst AH, ten Bokkel Huinink D, et al. The effect of a geriatric evaluation on treatment decisions for older cancer patients–a systematic review. *Acta Oncol* 2014;**53**(3):289-96. doi: 10.3109/0284186X.2013.840741

57. Extermann M, Hurria A. Comprehensive geriatric assessment for older patients with cancer. *J Clin Oncol* 2007;**25**(14):1824-31. doi: 10.1200/JCO.2007.10.6559

58. Lycke M, Pottel L, Boterberg T, et al. Integration of geriatric oncology in daily multidisciplinary cancer care: The time is now. *Eur J Cancer Care (Engl)* 2015;**24**(2):143-46. doi: 10.1111/ecc.12301

59. Chapman AE, Swartz K, Schoppe J, et al. Development of a comprehensive multidisciplinary geriatric oncology center, the Thomas Jefferson University Experience. *J Geriatr Oncol* 2014;**5**(2):164-70. doi: 10.1016/j.jgo.2014.01.003 (published online first: 1 February 2014).

60. Bouzereau V, Le Caer F, Guardiola E, et al. Experience of multidisciplinary assessment of elderly patients with cancer in a French general hospital during 1 year: a new model care study. *J Geriatr Oncol* 2013;**4**(4):394-401. doi: 10.1016/j.jgo.2013.04.006 (published online first: 21 May 2013).

61. Terret C, Zulian GB, Naiem A, et al. Multidisciplinary approach to the geriatric oncology patient. *J Clin Oncol* 2007;**25**(14):1876-81. DOI: 10.1200/JCO.2006.10.3291

62. Mbah O, Ford JG, Qiu M, et al. Mobilizing social support networks to improve cancer screening: The COACH randomized controlled trial study design. *BMC Cancer* 2015; 15(1):907. doi: 10.1186/s12885-015-1920-7

63. Fraser M. Thinking differently about the cancer workforce.Nuffield Trust 2016. http://www.nuffieldtrust.org.uk/blog/thinking-differently-about-cancer-workforce (accessed January 2017).

64. Glajchen M. Education, Training, and Support Programs for Caregivers of Individuals with Cancer. In: Talley RC, McCorkle R, Baile WF, eds. Cancer Caregiving in the United States: Research, Practice, Policy: Rosalynn Carter Institute for Caregiving Caregiving: Research-Practice-Policy series. New York and Heidelberg: Springer, 2012:79-102.

65. Muss HB. Cancer in the Elderly: a Societal Perspective from the United States. *Clin Oncol* 2009;**21**(2):92-98. doi: 10.1016/j.clon.2008.11.008 [published online first: 6 December 2008].

66. Brodie H. Getting the skill mix right. *Nurs Older People* 2013;**25**(6):10-10.

For peer review only

SUPPLEMENTARY FILE 1

Full search strategy

EBSCO HOST
Medline, CINHAL, PsycINFO, AMED: text terms and MeSH headings

1. TI Elderly OR AB Elderly
2. TI Geriatric* OR AB Geriatric*
3. TI “Older people” OR AB “Older people”
4. TI “Older patient*” OR AB “Older patient*”
5. TI “Older person” OR AB “Older person”
6. TI “Older adult*” OR AB “Older adult*”
7. MM Aged
8. MM Frail Elderly
9. **1 or 2 or 3 or 4 or 5 or 6 or 7 or 8**

10. TI Cancer OR AB Cancer
11. TI Oncolog* OR AB Oncolog*
12. MM Neoplasms
13. **10 or 11 or 12**

14. TI Workforce OR AB Workforce
15. TI “Health professionals” OR AB “Health professionals”
16. TI “Healthcare professionals” OR AB “Healthcare professionals”
17. TI “Health care professionals” OR AB “Health care professionals”
18. TI “Health personnel” OR AB “Health personnel”
19. TI “Healthcare personnel” OR AB “Healthcare personnel”
20. TI “Health care personnel” OR AB “Health care personnel”
21. TI “Medical personnel” OR AB “Medical personnel”
22. TI “Advanced Practice nurse” OR AB “Advanced Practice Nurse”
23. TI “Clinical nurse specialist” OR AB “clinical nurse specialist”
24. TI Geriatrician* OR AB Geriatrician*
25. TI Gerontologist* OR AB Gerontologist*
26. TI “Allied health professionals” OR AB “Allied health professionals”
27. TI Training
28. TI Educat*
29. TI “Skill mix” OR AB “Skill mix”
30. TI “Grade mix” OR AB “Grade mix”
31. TI “Staff development” OR AB “Staff development”
32. TI Staff* W1 level* OR AB Staff* W1 level*
33. TI Teamwork OR AB Teamwork
34. MM Health manpower
35. MM Health personnel
36. MM Attitude of Health personnel
37. MM Professional Competence
38. MM Staff development
39. MM Education, professional
40. MM Nurse’s role
41. MM Geriatric assessment

For peer review only

42. MM Health services for the aged
43. or/ 14-42
44. 9 AND 13 AND 43
45. English language filter

Embase (OVID)

1. elderly.ti,ab

2. geriatric.ti,ab

3. "older people".ti,ab.

4. "older patient*".ti,ab.

5. "older adult*".ti,ab.

6. "older person".ti,ab.

7. 1 or 2 or 3 or 4 or 5 or 6

8. cancer.ti,ab.

9. oncolog*.ti,ab.

1o. Neoplasm/

11. 8 or 9 or 10

12. workforce.ti,ab.

13. "health professionals".ti,ab.

14. "healthcare professional*".ti,ab.

15. "health care professional*".ti,ab.

16. "health personnel".ti,ab.

17. "healthcare personnel".ti,ab.

18. "health care personnel".ti,ab.

19. "medical personnel".ti,ab.

20. "Advanced Practice nurse".ti,ab.

21. "Clinical nurse specialist".ti,ab.

22. Geriatrician*.ti,ab.

23. Gerontologist*.ti,ab.

24. "Allied health professionals".ti,ab.

25. training.ti

For peer review only

26. educat*.ti

27. Skill Adj1 mix.ti,ab.

28. Grade Adj1 mix.ti,ab.

29. “staff development”.ti,ab.

30. “Workforce development”.ti,ab.

31. Staff adj1 level.ti,ab.

32. health care manpower/

33 professional competence/

34 vocational education/

35. health personnel attitude/

36. or/12-35

37. English.lg.

38. 7 AND 11 AND 36 AND 37

Scopus (Elsevier)

TITLE-ABS-KEY (cancer OR oncology OR neoplasm*) AND TITLE-ABS-KEY (elderly OR geriatric OR "older people" OR "older adult*" OR "older patient*" OR "older person") AND TITLE-ABS-KEY (workforce OR "health professional*" OR "health care professional*" OR “healthcare professional” OR "health personnel" OR "medical personnel" OR "advanced practice nurse" OR "clinical nurse specialist" OR geriatrician OR gerontologist OR "skill mix" OR "staff development" OR manpower)) AND (LIMIT-TO (LANGUAGE , "English"))

Web of Science (Thompson Reuters)

TITLE: (cancer OR oncolog* OR neoplasm*) AND TITLE: (elderly OR geriatric* OR "older people" OR "older person" OR "older adult*" OR "older patient*") AND TITLE: ("workforce" OR "health professional*" OR "healthcare professional*" OR “health care professional” OR "health personnel" OR "medical personnel" OR "advanced nurse practitioner" OR "clinical nurse specialist" OR geriatrician OR gerontologist OR "allied health professional*" OR training OR "educat*" OR "skill mix" OR "grade mix" OR "staff development")

Refined by: LANGUAGES: (ENGLISH)

Timespan=All years

CENTRAL

Elderly OR geriatric OR “older people” OR “Older adult*” OR “older patient*” OR “older person”

AND cancer OR oncolog* OR neoplasm*

In Title OR Abstract OR Key Words

Search for Trials

AgeInfo Cancer or Oncology in Title

For peer review only

SUPPLEMENTARY FILE 2 DATA EXTRACTION TABLE WITH EXAMPLE

#	STUDY AND AIM	STUDY DESIGN DATA COLLECTION MEASURES ANALYSIS	SETTING AND SAMPLE<br><br>COUNTRY CLINICAL SETTING PATIENT GROUPS STAFF GROUP	INTERVENTION	OUTCOMES	STAFF ROLE OR WORKFORCE DEVELOPMENT	RESULTS
1	Basu *et al.* (2013)<br><br>To evaluate timeliness in the period from breast cancer diagnosis to initial oncology consultation for a 9-month period prior to and following implementation of the nurse navigator.	Before and after study	USA<br><br>One cancer centre<br><br>All women diagnosed within the institution with stage 0 to III breast cancer at initial presentation<br><br>176 F: 100 (57%) in prenavigation group) and 76 (43%) in post navigation group<br><br>Stratified by age <61 and 61+<br><br>In the 61+ age group there were 86 patients 52 in the pre navigation group<br>34 in the post	A dedicated breast cancer nurse navigator to assist patients with their care. The navigator's involvement begins when the patient is diagnosed and ends when the patient is appointed into the survivorship clinic.	The time interval from date of diagnosis to date of initial oncology appointment was measured in business days. Date of diagnosis was defined as the patient's first positive biopsy date. The date of initial oncology appointment was defined as the first date the patient had a consultation with a surgical, medical, or radiation oncologist. In a few	Collaborating with physicians and interdisciplinary team members to triage patients, coordinating patient care, educating patients and providing links to research and other relevant resources, and serving as the primary point of contact for patients and their families. The nurse navigator calls recently diagnosed patients within 24 hours of biopsy, meets patients at the initial visit, and assists patients in scheduling oncology	Overall, the median wait time was 10 days for all patients in the study and ranged from 1 to 32 days. The median wait time for patients in the prenavigation group was 11 days compared with 9 days for patients in the postnavigation group.<br><br>Because of interaction between navigation and age identified during the modeling process, stratified models presented for each of the 2 categories of age<br><br>For patients aged 61

SUPPLEMENTARY FILE 2 DATA EXTRACTION TABLE WITH EXAMPLE

#	STUDY AND AIM	STUDY DESIGN DATA COLLECTION MEASURES ANALYSIS	SETTING AND SAMPLE<br><br>COUNTRY CLINICAL SETTING PATIENT GROUPS STAFF GROUP	INTERVENTION	OUTCOMES	STAFF ROLE OR WORKFORCE DEVELOPMENT	RESULTS
			navigation group<br><br>Breast cancer nurse navigator		cases a phone call from the oncologist to discuss pathology results and treatment options was recorded as the consultation date if there was no other consultation before treatment.	appointments or any necessary diagnostic tests. Patients are then tracked during the course of care to ensure timeliness of appointments and therapies and to provide referrals to treatment and supportive services as needed.	years and older, the median wait time was 4 days shorter for the navigation group (8 days versus 12 days).<br><br>In the 61 or older age group, navigation was a significant predictor of time to consultation, adjusting for demographic and clinical factors (P<.0002). In this adjusted model, the unstandardized beta coefficient for navigation was 24.9, indicating that with navigation, time to consultation decreased by an average of almost 5 days

For peer review only

For peer review only

PRISMA CHECKLIST (SUPPLEMENTARY FILE 3)[19]

Section/topic	#	Checklist item	Reported on page #
**TITLE**			
Title	1	Identify the report as a systematic review, meta-analysis, or both.	1
**ABSTRACT**			
Structured summary	2	Provide a structured summary including, as applicable: background; objectives; data sources; study eligibility criteria, participants, and interventions; study appraisal and synthesis methods; results; limitations; conclusions and implications of key findings; systematic review registration number.	2
**INTRODUCTION**			
Rationale	3	Describe the rationale for the review in the context of what is already known.	4
Objectives	4	Provide an explicit statement of questions being addressed with reference to participants, interventions, comparisons, outcomes, and study design (PICOS).	4
**METHODS**			
Protocol and registration	5	Indicate if a review protocol exists, if and where it can be accessed (e.g., Web address), and, if available, provide registration information including registration number.	7
Eligibility criteria	6	Specify study characteristics (e.g., PICOS, length of follow-up) and report characteristics (e.g., years considered, language, publication status) used as criteria for eligibility, giving rationale.	5-7
Information sources	7	Describe all information sources (e.g., databases with dates of coverage, contact with study authors to identify additional studies) in the search and date last searched.	5
Search	8	Present full electronic search strategy for at least one database, including any limits used, such that it could be repeated.	6-7
Study selection	9	State the process for selecting studies (i.e., screening, eligibility, included in systematic review, and, if applicable, included in the meta-analysis).	7
Data collection process	10	Describe method of data extraction from reports (e.g., piloted forms, independently, in duplicate) and any processes for obtaining and confirming data from investigators.	7
Data items	11	List and define all variables for which data were sought (e.g., PICOS, funding sources) and any assumptions and simplifications made.	7

For peer review only

Section/topic	#	Checklist item	Reported on page #
Risk of bias in individual studies	12	Describe methods used for assessing risk of bias of individual studies (including specification of whether this was done at the study or outcome level), and how this information is to be used in any data synthesis.	7
Summary measures	13	State the principal summary measures (e.g., risk ratio, difference in means).	7
Synthesis of results	14	Describe the methods of handling data and combining results of studies, if done, including measures of consistency (e.g., I^2) for each meta-analysis.	7
Risk of bias across studies	15	Specify any assessment of risk of bias that may affect the cumulative evidence (e.g., publication bias, selective reporting within studies).	24-26
Additional analyses	16	Describe methods of additional analyses (e.g., sensitivity or subgroup analyses, meta-regression), if done, indicating which were pre-specified.	N/A
**RESULTS**			
Study selection	17	Give numbers of studies screened, assessed for eligibility, and included in the review, with reasons for exclusions at each stage, ideally with a flow diagram.	8
Study characteristics	18	For each study, present characteristics for which data were extracted (e.g., study size, PICOS, follow-up period) and provide the citations.	Tables 2-7
Risk of bias within studies	19	Present data on risk of bias of each study and, if available, any outcome level assessment (see item 12).	9-10
Results of individual studies	20	For all outcomes considered (benefits or harms), present, for each study: (a) simple summary data for each intervention group (b) effect estimates and confidence intervals, ideally with a forest plot.	11-24
Synthesis of results	21	Present results of each meta-analysis done, including confidence intervals and measures of consistency.	N/A
Risk of bias across studies	22	Present results of any assessment of risk of bias across studies (see Item 15).	24
Additional analysis	23	Give results of additional analyses, if done (e.g., sensitivity or subgroup analyses, meta-regression [see Item 16]).	N/A
**DISCUSSION**			
Summary of evidence	24	Summarize the main findings including the strength of evidence for each main outcome; consider their relevance to key groups (e.g., healthcare providers, users, and policy makers).	24-26
Limitations	25	Discuss limitations at study and outcome level (e.g., risk of bias), and at review-level (e.g., incomplete retrieval of identified research, reporting bias).	24-26

For peer review only

Conclusions	26	Provide a general interpretation of the results in the context of other evidence, and implications for future research.	24-26
**FUNDING**			
Funding	27	Describe sources of funding for the systematic review and other support (e.g., supply of data); role of funders for the systematic review.	27

For peer review only

BMJ Open

Workforce characteristics and interventions associated with high quality care and support to older people with cancer: a systematic review

Journal:	*BMJ Open*
Manuscript ID	bmjopen-2017-016127.R1
Article Type:	Research
Date Submitted by the Author:	28-Mar-2017
Complete List of Authors:	Bridges, Jackie; University of Southampton; NIHR CLAHRC Wessex<br>Lucas, Grace; University of Southampton, Faculty of Health Sciences<br>Wiseman, Theresa; Royal Marsden Hospital; University of Southampton, Faculty of Health Sciences<br>Griffiths, Peter; University of Southampton, Faculty of Health Sciences; NIHR CLAHRC Wessex
**Primary Subject Heading**:	Oncology
Secondary Subject Heading:	Geriatric medicine
Keywords:	Human resource management < HEALTH SERVICES ADMINISTRATION & MANAGEMENT, Adult oncology < ONCOLOGY, GERIATRIC MEDICINE

SCHOLARONE™ Manuscripts

For peer review only

TITLE PAGE

Workforce characteristics and interventions associated with high quality care and support to older people with cancer: a systematic review

Corresponding Author:
Professor Jackie Bridges
Professor of Older People's Care
Faculty of Health Sciences
University of Southampton
NIHR CLAHRC Wessex

Nightingale Building
University Road
Highfield Campus
Southampton SO17 1BJ
Tel.+44(0)23 8059 8282
Email jackie.bridges@soton.ac.uk

Grace Lucas
Faculty of Health Sciences
University of Southampton
Southampton
UK

Professor Theresa Wiseman
Faculty of Health Sciences
University of Southampton
Southampton
UK

Professor Peter Griffiths
Faculty of Health Sciences
University of Southampton
NIHR CLAHRC Wessex
Southampton
UK

Word count (excluding title page, abstract, references, figures and tables): 4286

For peer review only

Workforce characteristics and interventions associated with high quality care and support to older people with cancer: a systematic review

ABSTRACT

Objectives: To provide an overview of the evidence base on the effectiveness of workforce interventions for improving the outcomes for older people with cancer, as well as analysing key features of the workforce associated with those improvements.

Design: Systematic review

Methods: Relevant databases were searched for primary research, published in English, reporting on older people and cancer and the outcomes of interventions to improve workforce knowledge, attitudes or skills; involving a change in workforce composition and/or skill mix; and/or requiring significant workforce reconfiguration or new roles. Studies were also sought on associations between the composition and characteristics of the cancer care workforce and older people's outcomes. A narrative synthesis was conducted and supported by tabulation of key study data.

Results: Studies (n=24) included 4,555 patients aged 60+ from targeted cancer screening to end of life care. Interventions were diverse and two thirds of the studies were assessed as low quality. Only two studies directly targeted workforce knowledge and skills and only two studies addressed the nature of workforce features related to improved outcomes. Interventions focused on discrete groups of older people with specific needs offering guidance or psychological support were more effective than those broadly targeting survival outcomes. Advanced Practice Nursing roles, voluntary support roles and the involvement of geriatric teams provided some evidence of effectiveness.

Conclusions: An array of workforce interventions focus on improving outcomes for older people with cancer but these are diverse and thinly spread across the cancer journey. Higher quality and larger scale research that focuses on workforce features is now needed to guide developments in this field, and review findings indicate that interventions targeted at specific sub-groups of older people with complex needs, and that involve input from advanced practice nurses, geriatric teams and trained volunteers appear most promising.

Keywords: Neoplasms, health manpower, older people, Geriatric medicine, Oncology, Health services for the aged, Nurse's role, Aged, health personnel

For peer review only

STRENGTHS AND LIMITATIONS OF THIS STUDY

- This novel synthesis provides evidence of promising interventions targeted at delivering high quality care to older people with cancer.
- It is an international systematic review articulating the evidence base on workforce interventions that may support high quality cancer care to an expanding ageing population.
- The review is limited to those studies where the role of the workforce was explicitly articulated within an intervention; there may be other studies in which changes or adaptations to the workforce were tested but not reported and are not included here.
- The review only included items published in the English language.

For peer review only

Workforce characteristics and interventions associated with high quality care and support to older people with cancer: a systematic review

BACKGROUND

More than 60% of new cancers and more than 70% of cancer deaths occur in people over the age of 65 years in Europe and the United States.[1] Treatment outcomes for older cancer patients vary internationally[2] and this may be linked to the extent to which services and their associated workforce effectively meet the more complex needs associated with an ageing population.[3 4] Many older people have comorbidities and limitations which affects their cognitive and physical functioning, their risk of complications and their emotional wellbeing[5] all of which may affect cancer treatment tolerance and necessitate a modified treatment plan and relevant supportive care.[6] More comprehensive assessment and management has been recommended to optimise older cancer patients for treatment.[6-8] Furthermore, older people may value a range of outcomes beyond survival at any cost, including maintaining independence and being able to access information, emotional support and practical support both during and after treatment.[9] Health care workers who organize and deliver cancer care thus need knowledge of clinical and other issues which are common in old age, but also need to be adept with the skills and values to enable them to support the patient and family, develop treatment plans, deliver appropriate care, and help older people to achieve the quality of life that reflects what matters most to them as individuals.[10]

While the specific role of the health care workforce in ensuring optimal outcomes and quality of life for older cancer survivors and their families has been recognised, evidence suggests that there are variations internationally in the preparedness of the workforce to meet the needs of an ageing population.[9-16] Issues identified include deficits in the necessary education, knowledge, skills and attitudes; in staffing levels and skillmix; and in the development of roles, teams and services that meet older people's needs.[17] However, little is known about the features and characteristics of the workforce associated with better outcomes for older people with cancer, or about the relative effectiveness of workforce-focused interventions which are aimed at improving cancer care and outcomes for an ageing population. This systematic review therefore aims to inform developments in policy and practice by providing an overview of the evidence base on the effectiveness of workforce interventions for improving the outcomes for older people with cancer, as well as analysing key features of the workforce associated with those improvements.

METHODS

Systematic methods were used to guide searching, selection and analysis.[18] Searches for primary research evaluating workforce interventions for older people with cancer were undertaken in August

For peer review only

2016. Studies were identified by searching electronic databases, scanning reference lists of articles and by contacting study authors. A detailed search strategy was tested in MEDLINE (Table 1). The search was additionally tailored for database-specific subject headings and applied in: PsycINFO, Cumulative Index to Nursing and Allied Health Literature (CINAHL), Allied and Complementary Medicine Database (AMED), Embase, Web of Science, Cochrane Central Register of Controlled Trials (CENTRAL), AgeInfo and Scopus (Supplementary File 1). Searches were limited to the English language. No date limit was applied to ensure a comprehensive overview of developments in the field.

For peer review only

Table 1 Example of Search Strategy MEDLINE (EBSCOHOST)

**Concept 1**	**Concept 2**	**Concept 3**
1. TI Elderly OR AB Elderly	10. TI Cancer OR AB Cancer	14. TI Workforce OR AB Workforce
2. TI Geriatric* OR AB Geriatric*	11. TI Oncolog* OR AB Oncolog*	**15.** TI "Health professionals" OR AB "Health professionals"
3. TI "Older people" OR AB "Older people"	12. MM Neoplasms	**16.** TI "Healthcare professionals" OR AB "Healthcare professionals"
4. TI "Older patient*" OR AB "Older patient*"	13. 10 or 11 or 12	**17.** TI "Health care professionals" OR AB "Health care professionals"
5. TI "Older person" OR AB "Older person"		18. TI "Health personnel" OR AB "Health personnel"
6. TI "Older adult*" OR AB "Older adult*"		**19.** TI "Healthcare personnel" OR AB "Healthcare personnel"
7. MM Aged		**20.** TI "Health care personnel" OR AB "Health care personnel"
8. MM Frail Elderly		21. TI "Medical personnel" OR AB "Medical personnel"
9. 1 or 2 or 3 or 4 or 5 or 6 or 7 or 8		22. TI "Advanced Practice nurse" OR AB "Advanced Practice Nurse"
		23. TI "Clinical nurse specialist" OR AB "Clinical nurse specialist"
		24. TI Geriatrician* OR AB Geriatrician*
		25. TI Gerontologist* OR AB Gerontologist*
		26. TI "Allied health professionals" OR AB "Allied health professionals"
		27. TI Training
		28. TI Educat*
		29. TI "Skill mix" OR AB "Skill mix"
		30. TI "Grade mix" OR AB "Grade mix"
		31. TI "Staff development" OR AB "Staff development"
		32. TI Staff* W1 level* OR AB Staff* W1 level*
		33. TI Teamwork OR AB Teamwork

For peer review only - http://bmjopen.bmj.com/site/about/guidelines.xhtml

		34. MM Health manpower
		35. MM Health personnel
		36. MM Attitude of Health personnel
		37. MM Professional Competence
		38. MM Staff development
		39. MM Education, professional
		40. MM Nurse's role
		41. MM Geriatric assessment
		42. MM Health services for the aged
		43. or/ 14-42
		44. 9 AND 13 AND 43
		45. English language filter

Eligible study types included randomised controlled trials (RCTs), quasi-experimental or observational studies with a clearly defined workforce variable or intervention with a comparison between different exposure levels, and qualitative studies evaluating features of the workforce from the perspective of older people with cancer and where the role of the workforce forms a central part of the research question. We defined workforce based interventions as any intervention where the main mode of action was through a change in the composition, roles, knowledge, skills or attitudes of individuals or groups in a care delivery role, paid or unpaid, not including family or informal caregivers. Papers included reported on studies conducted with participants identified as older people (age 60+) at any stage in the cancer journey (from targeted screening through to end of life). Papers included reported on either:

- Outcomes of interventions to improve the knowledge, attitudes or skills of the workforce delivering cancer care and treatment to older people;
- Outcomes of interventions involving a change in the composition and/or skill mix of the workforce delivering cancer care for older people including (but not limited to) role substitution, new roles or adding specialist practitioners to the team;
- Outcomes of interventions routinely targeted at older people with cancer, which were reported to require significant workforce reconfiguration or the implementation of new roles; and/or
- Associations between the composition and characteristics of the cancer care workforce (including, but not limited to, staffing levels, skill mix, training, knowledge attitudes and skill) and outcomes for older people with cancer.

Studies reporting solely on drug, treatment or other therapeutic interventions (without specific focus on the workforce delivering those interventions) were not included.

For peer review only

Titles and abstracts from the searches were screened against the inclusion criteria by GL to exclude irrelevant papers. Five percent of titles/abstracts were also independently reviewed by another team member (JB, PG or TW) to confirm exclusion decisions. Full-text papers were retrieved for all papers that screened positively against inclusion criteria or about which a clear decision could not be taken (due to lack of information). Each full text paper was reviewed independently by two team members followed by a decision to include or exclude. These reviews were followed by further team discussion to finalise inclusion. The search and selection process is summarised in the PRISMA flow chart (Figure 1).[19]

Data on aim, design, setting, sample, intervention, outcome and results were extracted systematically from eligible papers using data extraction tables developed by the team (supplementary file 2). We adapted the GRADE system as used by Cochrane for rating evidence[18] to guide a broad assessment of individual study quality and thereby the contribution studies made to the review. Initial quality ratings based on study design were upgraded or downgraded depending on presence of factors considered to strengthen or weaken the evidence. Two members of the team independently reviewed all included papers. Discrepancies were discussed and ratings confirmed through discussions involving both raters and a third team member. No studies were excluded based on this assessment but lower quality studies were given less weight in the analysis.

Due to the heterogeneity of interventions and outcomes, a narrative analysis of study findings was merited.[20] Studies were grouped around the patient or service problems the interventions were targeting. Results were tabulated and the findings of effectiveness of individual interventions were plotted within these groups and used as the basis for an analysis of the strength of evidence of effectiveness across these groups and the field as a whole. We recorded and tabulated both the direction of differences between groups (where reported) and statistical significance of differences. Due to the number of different outcomes across the 24 studies, we report, within the results section, for the primary outcomes where there is evidence of significant differences between groups, rather than narrating the full set of results for each individual paper. A review protocol is available from the study team on request.

RESULTS

We identified 24 eligible published journal papers (23 quantitative and 1 qualitative study) covering 22 interventions and reporting on 4,555 patient participants age 60+ from targeted screening, through cancer diagnosis and treatment and beyond. All but one study were conducted in USA or Europe. The studies report on 27 individual primary outcomes and 42 individual secondary outcomes (using a range of measures) across the studies corresponding to 41 different outcomes in total (n=38 of these were patient related outcomes and the other three outcomes were focused

For peer review only - http://bmjopen.bmj.com/site/about/guidelines.xhtml

directly on the workforce). As detailed below and illustrated in Table 2, 17 studies were assessed as low or very low quality, with four studies rated as medium and three as high quality.

The point of the cancer journey each intervention was targeted at varied widely. Interventions ranged from targeted screening stage (n=1) and from diagnosis (n=4); to treatment phase/hospital stay (n=11); to those primarily focused on patients after the completion of their treatment (n=6); hospice care (n=1) or home care for advanced cancer patients (n=1). The majority of the interventions were limited to specific tumour types: 15 involved participants with a range of cancer types, but some involved more homogeneous populations: six were for breast cancer patients, one intervention targeted prostate patients, another involved those with gastrointestinal cancers, and one was aimed at breast and cervical screening.

Only two interventions were directly targeted at improving the knowledge, attitudes or skills of the workforce delivering cancer care and treatment to older people through training[21 22] and only two studies directly addressed the second objective of the review to assess the salient features of the cancer care workforce: one qualitative study considered the features of the nursing workforce which older patients felt were important in their care[23] and one study looked at the impact of healthcare professionals communication on participants' views about their symptom management.[24] The remaining studies reported on improving older people's outcomes via interventions involving a change in the workforce. In five interventions new roles were tested: nurse navigator,[25] depression care manager,[26] nurse case manager,[27] telephone support (trained graduates),[28] and social support volunteers.[29] In other studies, support from additional workforce members was provided to patients. Four studies reported on the increased involvement of a geriatrician or a geriatrics team,[27 30-32] one reported on the input of a clinical pharmacist,[33] and one study reported on the input of an additional dietician.[34] In two studies, a current staff member had a different function; in one study a nurse provided targeted cancer screening [35] and in another study a physiotherapist designed exercise and yoga programmes.[36] Three interventions used Advanced Practice Nurses – one in a homecare capacity[37] and two in counselling roles.[38 39] In three studies the role of multidisciplinary team members was highlighted.[40-42] In some papers, although a named member or members of the workforce were reported to have implemented or carried out the intervention, it was unclear as to the exact nature of their position. This was the case with two studies using exercise physiologists where it could not be determined if they were existing or new staff members.[43],[39] Only seven studies referred to an explicit theoretical framework or model in intervention design.[21 22 24-26 38 39]

Because of the heterogeneity of studies retrieved (and the small number of studies that addressed the review's second objective), we reviewed evidence of the effectiveness of interventions by study

For peer review only - http://bmjopen.bmj.com/site/about/guidelines.xhtml

type established through particular problems (related to older people with cancer) that the respective interventions were addressing and, subsequently, ways in which workforce requirements were being adapted to meet needs and improve outcomes related to these patient problems.. The results in Table 2 and set out below are displayed using these individual types.

Regular and timely access to care and treatment

Four studies focused on interventions targeted at the problem of systemic delays or inequitable access to treatment in the cancer journey for older people. They provide some promising evidence that providing additional support to some groups of older cancer patients can help them navigate the system and access treatment thereby improving the speed and efficacy of care. However, three of these papers provide only low quality evidence.

A high quality RCT reported that older women with breast cancer in the care of a nurse case manager acting as an educator, counsellor and coordinator were significantly more likely to see a radiation oncologist as part of initial evaluation, and to receive breast-conserving surgery and radiation therapy.[27] Further, the difference in receipt of appropriate treatment between women with characteristics associated with lower rates of appropriate treatment (75+, being unmarried, living alone, and being a member of an ethnic minority group) and their respective comparison groups were diminished or eliminated in the intervention group. An observational study reported that a breast cancer nurse navigator providing support and coordination of patient care from diagnosis until entry into survivorship clinic significantly shortened time to consultation for patients aged 61+ years.[25] A nurse practitioner role was used in a quasi-experimental study to improve screening rates for older Black women of low socioeconomic status by offering screening during a routine visit.[35] Nurse practitioner follow-up screening rates were significantly higher than baseline, compared with control group follow-up rates. A further study assessed the impact of a geriatrician consultation and treatment plan through an analysis of registry data of older breast cancer patients.[32] Patients who had a consultation had more co-morbidities and more advanced and aggressive tumours, were more likely to receive mastectomy and adjuvant therapy, and were less likely to be treated by breast-conserving surgery and adjuvant therapy.

Complications and specific problems from cancer treatment

Four studies reported the use of workforce members with specialist skills to address cancer treatment complications and impact on mortality and survival. None of the three low quality studies found any intervention effect on mortality rates, but the one high quality RCT found that specialised home care advanced practice nurses (used to enhance surgical recovery) increased two year survival for late stage cancer patients in the intervention group.[37]

For peer review only - http://bmjopen.bmj.com/site/about/guidelines.xhtml

Other lower quality studies in this group included evaluations of face-to-face counselling to address nutritional intake for patients treated with chemotherapy and at risk of malnutrition,[34] an intervention focused on the prevention of post-operative delirium with input from a geriatric team,[30] and comprehensive geriatric assessment (CGA) targeted at chemotherapy tolerance and toxicity.[44] The observational study evaluating CGA for older chemotherapy patients found that CGA patients were more likely to complete cancer treatment as planned but no significant differences were found in relation to mortality or other outcomes measures in relation to the interventions in any of these three studies.

Co-morbidities and complex health needs

The five studies reported here target the health issues that may accompany a cancer diagnosis, but also broader health problems that may not directly relate to the cancer. They highlight the importance of recognising and addressing these needs, although the range of outcomes and the variable quality of evidence (three studies of medium quality; two were low quality) make it difficult to draw firm conclusions about the best use of workforce support in this sizeable area.

A cluster RCT evaluating a hospice staff training programme on improving pain assessment and management did not find significant practice improvements or decreases in patient pain severity associated with the intervention.[22] In a different study, a secondary analysis of RCT data on the impact of a depression care manager providing education and support for older patients with depression found that intervention patients with a cancer diagnoses were twice as likely to experience a depression treatment response at 12 months compared to usual care.[26] Rao *et al.* also reviewed the outcomes for cancer patients from a wider RCT evaluating the impact of involving a geriatric team in the care of inpatient and outpatients diagnosed with frailty.[31] The inpatient intervention group showed significant improvements in bodily pain and mental health versus the usual inpatient care group but there was no impact on survival rates. There were no intervention effects on outpatients. An uncontrolled before and after study, reported that using a clinical pharmacist to identify patients' potentially inappropriate medications (PIMs) reduced the number of PIMs at discharge versus admission.[33] A low quality RCT reported that intensified primary healthcare support significantly reduced the number of days in hospital for an intervention group of advanced cancer patients compared to patients receiving standard care.[41]

Quality of life, physical and psychological functioning

Eight studies focused on addressing quality of life (QOL) across its physical and psychological aspects. This group of interventions used a range of workforce members (often in therapeutic or supportive roles) from physiotherapists to advanced practice nurses to trained voluntary input, to address a

For peer review only

range of factors underpinning quality of life. They showed mixed evidence of effectiveness. Seven of the studies in this group provided low quality evidence.

Three studies focused on physical functioning in particular. In an RCT with low recruitment rate and possible selection bias, exercise physicians provided Qi exercise training.[39] Both usual care and intervention participants increased their activity levels but the extent of the increase was significantly greater in the intervention group. The intervention also used advanced practice nurses delivering face-to-face counselling and significant improvements in symptom experience, self-efficacy and self-esteem were reported. A controlled before and after study compared the effect of yoga classes (with the input of a physiotherapist/yoga teacher) with a standard exercise programme.[36]

QOL scores after the program were better than before for both groups, but some QOL parameters improved more for those included in the yoga intervention. A pilot RCT with small sample and high dropout compared two exercise forms implemented by a physiologist (compared to usual care) and found significant activity increases for the group using a home-based walking and resistance intervention.[43]

Two similar interventions involved a multidisciplinary team approach for a range of quality of life domains, however both of these secondary analyses reported on very small samples sizes of older adults within wider QOL interventions. Lapid *et al.*[42] found in a secondary analysis of a small sample of patients in a wider RCT, that higher QOL scores were reported for older patients who received multidisciplinary emotional and practical support. However, in the study by Chock et al,[40] the authors did not find any lasting differences on QOL for older intervention participants against their younger counterparts, apart from an improvement in anger-hostility.

Advanced practice nurses were used in a symptom management intervention in the two pilot RCTs and the observational study reported by Heidrich *et al.*[38] Some evidence of effectiveness was reported for improving self-care and reducing symptom distress and duration, but there was no impact on QOL.

Two studies used trained volunteers to bolster psychological support. A secondary analysis of RCT data was used to evaluate the effect of using trained graduate support workers to provide initial distress monitoring to patients over the telephone. [28] Intervention patients had significantly lower anxiety and depression at six months than patients receiving educational materials alone. However, no other differences in psychological wellbeing were detected. Mantovani *et al.*[29] also used trained support volunteers to provide emotional and practical support. An RCT with small sample size was

For peer review only

used to compare this support with pharmacological treatment alone, and further with the addition of psychotherapy. Significant improvements in anxiety and depression were reported for the groups receiving voluntary support and/or additional psychotherapy. However, there were no significant differences on other QOL measures.

Communication between health care professionals and older people with cancer

Three studies focused on addressing the communication needs of older people with cancer. One high quality study offered communication skills training to staff with varied success[21] and the other two low quality studies highlighted the importance of good communication as a prerequisite for cancer nurses related to improving older patients' quality of life.

A cluster RCT found that training nursing staff to improve chemotherapy patient education led to a significant, positive effect for 'discussing realistic expectations'.[21] Significantly less history taking was also observed pre to post in the intervention group, as well as less talking about all the possible side effects; both points of attention during training. No other significant effects were reported. Yeom and Heidrich[24] used a cross-sectional analysis of RCT data to report that communication difficulties with health professionals had significant direct, negative effects on QOL dimensions. Findings from a qualitative interview study highlighted the value to older cancer patients of nurses having a person-centred manner, with the ability to show a genuine and empathic interest in the patients and to make a connection with good listening and communication skills.[23]

DISCUSSION

This systematic review aimed to provide an overview of the evidence base on the effectiveness of workforce interventions for improving the outcomes for older people with cancer, as well as analysing key features of the workforce associated with those improvements. Findings reflect a range of ways in which the workforce has been adapted, expanded or trained to addressing older cancer patients multiple and divergent needs. The findings present a novel synthesis of the type of interventions being developed globally to address the broad question of how the workforce can support the improvement of older people's cancer outcomes. The approaches and the patient problems they are addressing are varied, including integrating the input of geriatric specialists into cancer services, using advanced practice nurse roles to support patients, creating new roles to guide patients through the healthcare system and ensuring effective treatment, through to novel approaches using voluntary support, or trialling yoga or other exercise to improve older patients' quality of life.

For peer review only

While the included studies begin to provide evidence about how the workforce can be used to make a tangible difference to older cancer patients' physical and psychological outcomes, the diversity of interventions in the studies reviewed and the range of outcomes evaluated, limits generalisations on effectiveness. Further, the quality of evidence is generally low. Experimental designs were not consistently used and, when they were, their implementation was often hampered by poorer than expected recruitment, or conclusions drawn about outcomes for older patients were drawn from a secondary analysis of a wider dataset. In addition, as is common in the reporting of complex intervention evaluations, details of the intervention itself were often lacking.[45] There was inadequate reporting of the specific workforce contribution to the interventions and limited evidence to address the second objective of the review around the features of the cancer care workforce associated with better outcomes. In addition, while staff training was involved in half of the interventions reported, the details of how that training worked or could be improved was not detailed. Furthermore, although some innovative roles were set up, the rationale and detail of those roles was often poorly reported.

Despite these shortcomings, these findings do provide some promising insights into how the workforce may address the varied needs of older cancer patients, albeit with a dearth of evidence at the earlier and later stages of the cancer journey. Evidence has suggested that not all older people with cancer need the same input, and indeed age-related changes occur at different rates in different individuals and are not reflected in chronologic age.[7] Therefore, it is more productive to focus attention on those with complex problems.[46] The studies in this review appear to support the notion of targeted assistance to groups at particular risk of under-treatment. Review findings suggest that broader interventions aiming to improve survival outcomes are less successful, but studies did indicate the kind of support that could be put in place after treatment to deal with the specific complications and problems that older people might face. One intervention which did improve survival used advanced practice nurses in home-care support post-surgery.[37] Indeed, the role of advanced practice nurses in the future of older people's cancer care has been acknowledged elsewhere in the literature,[47-50] and this review indicates that this is a candidate role for exploration and further consideration.

The input of geriatric specialists who are able to assess and manage older patients and optimise patients for treatment was a significant feature of several studies reviewed and formal links and services are well established in some countries.[51-53] Findings from this review provide weak evidence of positive benefits from the input of geriatricians but it only included studies where the geriatrician's role was explicit in the intervention and where a comparison or control was featured.

For peer review only

There are a number of other reviews reporting on specialist geriatric assessment and management for older cancer patients, and these have been able to draw firmer conclusions about the benefits of CGA with older cancer patients, although they all acknowledge the need for more definitive research.[54-56] Multidisciplinary approaches also emerged as a feature across the studies reviewed and the need to shape teams around the multiple needs of older people with cancer has been highlighted elsewhere, although evidence from this review is weak, again limited by the scale and quality of the research.[6 57-60]

Of further interest is the use of non-professionals in providing direct care services to older people with cancer, and roles such as these are relevant in the contexts of budgetary pressures and recruitment difficulties of key professional groups such as geriatricians and registered nurses.[17] The two studies reviewed suggested a positive impact on patient outcomes and align with a growing recognition of the non-clinical workforce (including carers and families) playing an essential role in older people's cancer care.[61-63] However, the low quality of the research again reduces confidence in these positive findings. A final point is that the studies identified for this review did not address the impact of staffing levels or skillmix on older cancer patients' outcomes. In addition, few mechanisms to develop the current workforce to prepare for and be supported to deliver high quality care to an ageing population were identified. In addition to the development and more definitive evaluation of new roles and practices, the future research agenda must address these important facets to ensure that, regardless of setting, all health care workers that older people with cancer encounter, are prepared for and adequately supported in their role.[64 65]

This review alone is insufficient to enable conclusions to be drawn about the workforce factors which prove most beneficial to older people's outcomes; further high quality RCTs are needed to assess the potential of possible interventions. Future research should build on the studies reviewed here to establish what workforce developments are needed to support this growing population throughout the cancer journey. The most promising interventions for further study target assistance to individuals with complex needs who are at particular risk of under-treatment, and of problems arising from cancer treatment or its impact. Our review indicates that the impact of multiprofessional teams, including geriatric physicians and advanced practice nurses, on patient outcomes from survival to quality of life, would be worthwhile to evaluate more definitively, as would the contribution of trained volunteers.

For peer review only

Table 2: Summary of studies included in review

Source; Study design; Final quality rating	Setting and sample	Intervention and workforce	Primary outcomes (secondary outcomes)	Results
**Regular and timely access to care and treatment**				
Basu *et al* 2013[25]<br><br>Observational: care control<br>++	Women aged 61+ with breast cancer<br>n=86<br><br>One cancer centre, USA	I: Patient Navigation: Support and coordination of patient care<br>T: From point of diagnosis to survivorship clinic<br>C: No navigation<br>W: Breast cancer nurse	Time from diagnosis to oncology appointment	Time to consultation decreased by 4.9 days (P=.0002)
Goodwin *et al.* 2003[27]<br><br>RCT<br>++++	Women aged 65+ newly diagnosed with breast cancer, n=335<br><br>13 community and two public hospitals, USA	I: Case management: nurse as educator, counsellor, advocate, and care coordinator<br>T: 12 months service<br>C: usual care [unclear]<br>W: Nurse case manager	Treatment received in 6 months after breast cancer diagnosis<br><br>(Patient satisfaction; arm function)	More intervention women saw radiation oncologist (36% vs 19.3%) (P=.006), received more breast-conserving surgery (28.6% vs 18.7%; P=.031) and radiation therapy (36.0% vs 19.0%; P=.003), had more breast reconstruction surgery (9.3% vs 2.6%, P=.054); reported choice in treatment (82.2% vs 69.9%, P=.020)<br>No differences between groups in percentages who saw an oncologist, discussed breast reconstruction, underwent complete surgical staging, or tissue sent for hormone receptor assay.
Mandelblatt *et al.* 1993[35]<br><br>Controlled before and after study<br>++	Women aged 65+ screening for breast or cervical cancer<br>n=673<br><br>Two public hospitals, USA	I: Screening intervention during routine visits<br>T: At scheduled appointments<br>C: Physician reminder system<br>W: Nurse practitioner	Annual screening rates for Pap tests and mammographies	Annual intervention site Pap test rate increased (17.8% to 56.9%), and mammographies (18.3% to 40%) compared to control site increase of Pap test rate from 11.8% to 18.2% and no change (18%) for mammography (P =.01).
Somana-Ehrminger *et al.* 2015[32]<br><br>Observational<br>++	Women aged 75+ with breast cancer<br>n=206<br><br>Breast and gynaecological cancer registry, France	I: Geriatrician referral and treatment plan<br>C: Patients with no geriatric oncology consultation<br>W:Geriatrician, dietician, psychologist, physical therapist or social worker	Independent impact of geriatric oncology consultation (GOC)	GOC patients more likely to receive mastectomy and adjuvant therapy (P <.0001); and less likely to be treated by breast-conserving surgery and adjuvant therapy (P =.003).
**Complications and specific problems of cancer treatment**				
Bourdel-Marchasson *et al.* 2013 [34]<br><br>RCT<br>++	Chemotherapy patients aged 70+ n=336<br><br>12 public and private settings, France	I: Face to face dietary counselling<br>T: 6 visits (3-6 months)<br>C: Usual care<br>W: Dietician	One year mortality<br><br>(Chemotherapy management; unplanned hospitalisation; 2year mortality)	Difference of 178kcal/day dietary intake in intervention group (P<.01). No difference in other outcomes.
Hempenius *et al.* 2013[30]<br><br>RCT<br>++	Frail adults aged 65+, elective surgery for solid tumour<br>n=260<br><br>Teaching hospital and community hospital, Netherlands	I: Delirium prevention: assessment, monitoring and individualised treatment plan<br>T: During hospital stay<br>C: Usual care. Additional geriatric care provided on referral.<br>W: Geriatric team supervised by a geriatrician.	Incidence of post-operative delirium up to 10 days<br><br>(Severity of delirium; length of hospital stay; complications; mortality; care dependency; quality of life)	Significant difference in return to preoperative living situation (67.3% vs.79.1%, Odds ratio (OR): 1.84, 95% confidence interval (CI): 1.01-3.37). No significant difference in other outcomes.
Kalsi *et al.* 2015[44]	Adults aged 70+ with cancer	I: Geriatrician comprehensive geriatric assessment and intervention plan (CGA) for identified need.	CGA impact on chemotherapy tolerance and toxicity; rate of planned completion of	Intervention more likely to complete planned cancer treatment (33.8% vs 11.4% OR 4.14 (95% CI: 1.50–11.42), P = 0.006) and fewer required

For peer review only

Source; Study design; Final quality rating	Setting and sample	Intervention and workforce	Primary outcomes (secondary outcomes)	Results
Observational<br>++	n=135<br><br>One hospital, UK	T: pre chemotherapy and further support as needed<br>C: Standard oncology care<br>W: Geriatrician	cancer<br><br>(Treatment modifications; early treatment discontinuation; death at six months)	treatment modifications 43.1% vs 68.6%, (OR 0.34 (95% CI: 0.16–0.73), P=0.006).<br>Lower toxicity rate in intervention group (43.8% vs 52.9%, P =.292).<br>No differences in death rates.
McCorkle *et al.* 2000[37]<br><br>RCT<br>++++	Adults aged 60+ post-surgical cancer patients<br>n=375<br><br>Comprehensive cancer centre, USA	I: Specialized home care advanced practice nurses (APNs) assess and monitor physical, emotional, and functional status of patients, provide direct care, access services and other resources from the community, and provide teaching, counseling and support during recovery.<br>T: 4 weeks with three home visits and five telephone contacts<br>C: Usual follow-up care in an ambulatory setting and routine outpatient follow-up<br>W: Advanced practice nurses (APNs)	Length of survival<br><br>(Depressive symptoms; symptom distress; functional status)	Late stage patients, improved 2 yr survival in intervention group: 66.7% v 39.6% (P < .05). No difference for early stage patients<br>Risk of death higher for control (adjusted hazard ratio 2.04; CI, 1.33-3.12; P= .001) compared with intervention group.<br>No differences between groups in other outcomes.
**Comorbidities and complex health needs**				
Deliens *et al.* 2016[33]<br><br>Uncontrolled before and after study<br>+	Adults aged 70+ with cancer (non-haematological) hospitalised<br>n=91<br><br>Geriatric oncology unit, tertiary hospital, Belgium	I: Medication review: Identification of PIMS and drug interactions<br>T: From point of admission and during hospitalisation<br>C: Before to after<br>W: Clinical pharmacist	Potentially inappropriate medications (PIMS) using START (screening tool to alert doctors to right treatment) and STOPP (screening tool of older person's potentially inappropriate prescriptions) criteria.<br><br>(Drug to drug interactions)	START criteria: 41 PIMS for 31 patients (34%) on hospital admission compared to 7 PIMS for 6 persons (7%) at discharge.<br>STOPP criteria: 50 PIMs for 29 patients (32%) at admission compared to 16 PIMS for 14 persons (16%) at discharge.
Fann, Fan and Unutzer 2009[26]<br><br>RCT<br>+++	Adults aged 60+ diagnosis of non-skin cancer and major depression or dysthymia<br>n=215<br><br>18 primary care clinics at 8 diverse health care organisations, USA	I: Depression management: education, 'behavioural activation', treatment support.<br>T: Up to 12 months. Followed up usual care 12 months more.<br>C: Usual care: received routinely available depression treatment<br>W: Depression care manager (nurse or clinical psychologist) collaborative with primary care.	Depression treatment response<br><br>(Health related quality of life (QOL); health-related impairments: work, family, social functioning; patient satisfaction**)**	Intervention twice as likely to experience a depression treatment response at 12 months than control (39% vs. 20%; P = .029) and at 18 months (38% vs. 16%; P = .012).<br>Remission rates higher in intervention group v control at 6 months (32% v 16% P = .006) and 12 months (22% v 9%, P = .031).<br>Less functional impairment at 12 months (P=.011) and greater QOL (P=.039).
Herr *et al.* 2012[22]<br><br>RCT (cluster)<br>+++	Adults aged 65+ with cancer receiving hospice care<br>n=738<br><br>Staff: Nurses (n=383 pre, n=415 post) and physicians (n=16)<br><br>16 hospices, USA	I: Workforce: to promote adoption of evidence-based pain practices. Included: training, assessment of data, champion input, senior leadership engagement.<br>T: Engagement phase 5 months, 12 month intervention.<br>C: Hospices received clinical practice guidelines<br>W: 3 days training. Selection of local pain facilitators, nurse and physician champions, Grant Expert Nurse input, Nurse and Physician Champion	Workforce: adoption of evidence-based cancer pain practices<br><br>(Pain severity)	No significant difference in improvement on Cancer Pain Practice Index between intervention and control.<br>Decrease in patient pain severity from pre to post in intervention group greater (p=.1032)
Johansson *et al.* 2001[41]<br><br>RCT	Adults aged 70+ newly diagnosed with prostate, GI or breast cancer<br>n=161	I: Intensified primary healthcare. Individual support: nurse support, nutritional support, and individual psychological support.<br>T: Starting from diagnosis	Utilisation of specialist care	Mean days of hospitalization for older intervention patients than control (3.8 v 8.9, P<.01).<br>4 of 82 older intervention patients admitted compared with 12 of 79 older control patients (P<.05).

For peer review only

Source;<br>Study design;<br>Final quality rating	Setting and sample	Intervention and workforce	Primary outcomes (secondary outcomes)	Results
++	Primary healthcare services, Sweden<br><br>(Other participants reported: n=255 under 70 years)	C: Standard care + group rehabilitation<br>W: Homecare nurse, dietician and psychologist.<br>GPs and nurses trained in pain, nausea and diet, in final stage life.		10 out of 82 made acute visits to outpatient clinics compared to 22 of 79 in control group (P<.05).
Rao *et al.* 2005[31]<br><br>RCT<br>+++	Adults aged 65+ with cancer, frail and hospitalized<br>n=99<br><br>11 medical centres, USA	I: Assessment and monitoring by geriatric team. 1. geriatric inpatient +usual outpatient; 2. Usual inpatient +geriatric outpatient; 3. Geriatric inpatient and outpatient<br>T: 1 year study<br>C: Usual care: all hospital services except from geriatric team<br>W: Core team: geriatric medicine attending physician, fellow or intern, a nurse practitioner, social worker.	Survival ; Health related quality of life<br><br>(Functional status; physical performance)	No difference: in survival for the cancer patients regardless of treatment group<br>Significant effect of geriatric inpatient care v usual inpatient care: mean change in score from randomization to discharge: bodily pain (28.7 v 10.1) P=.09; emotional limitation (29.3 v 2.7) P=.01. Effect on bodily pain sustained at 1 year (37.6 v 9.9).<br>No effect of geriatric outpatient care on any of the QOL parameters.<br>No effect of either inpatient or outpatient geriatric care on the functional status of cancer patients.
**Quality of life, physical and psychological functioning**				
Chock *et al.* 2013[40] (Secondary analysis of Clark et al. 2013)[66]<br><br>RCT<br>++	Adults aged 65+ with advanced cancer treated with radiotherapy<br>n=16<br><br>Cancer centre, USA<br><br>(Other participants reported: n=38 under 65 years)	I: QOL intervention with telephone follow up: Physical therapy, education, cognitive behavioural interventions, discussion and support, spiritual reflection and relaxation training<br>T: 6 sessions 90 mins, 2-4 weeks and 10 brief structured telephone sessions<br>C: Standard care<br>W: Multidisciplinary (including physical therapist, clinical psychologist, APN, Chaplain)	Quality of life; mood	Significant difference at week 4 only in mean overall QOL older v younger adults (74.4 vs 62.9, P=.040).<br>No significant difference in<br>Significantly lower Anger-Hostility dimension of mood measure at all weeks for older v younger patients. Week 4: 95.0 vs 86.4, P=.028; wk 27: 92.2 v 84.2, P=.027) and wk 52 (96.3 v 85.9, P=.005).<br>No other significant differences.
Heidrich *et al.* 2009[38]<br><br>Two pilot RCTs and one observational study<br>++	Women aged 65+, 1 year post diagnosis of non-metastatic breast cancer<br>n=82 (total)<br><br>Oncology clinics, cancer centre, USA	I: Pilot 1 - Symptom management (IRIS): counselling interview and telephone follow up on symptom management at 4 weeks; Pilot 2- Addition of four biweekly telephone reinforcement sessions; Pilot 3 - Intervention by phone only<br>C: 1 -usual care; 2-delayed IRIS (waitlist) control; 3 - no control: (IRIS Group only)<br>T: 4 weeks (pilot 1)<br>W: Advanced practice nurses	Feasibility, acceptability<br><br>(Symptom distress; symptom management; quality of life; mood; barriers to symptom management; communication difficulty)	Feasibility: Across all studies, 76% of eligible women participated, 95% completed the study, 88% reported the study was helpful, and 91% were satisfied with the study.<br>**Pilot 1:** No significant difference in symptom distress. Significant decrease in distress baseline to follow up in intervention group; Significantly more women in intervention reported changing self-care of symptoms (P<.05); No significant QOL differences.<br>**Pilot 2:** Significant less symptom duration compared to control at 8 weeks (P <.01). At 16 weeks, intervention group more likely to have talked to healthcare provider, begun new symptom treatment, and changed self-care symptoms (P<.05). No significant QOL differences. Negative attitudes from healthcare providers reported by 5%–20% of women and communication difficulties by 5%–45% of women.<br>**Pilot 3:** No significant differences (no control) baseline to eight weeks. Symptom interference decreased (and negative mood from symptoms). Symptom duration interference, and negative mood from symptoms decreased. No QOL change.
Kornblith *et al.* 2006[28]	Adults aged 65+ with breast, colon or prostate cancer	I: Telephone monitoring of distress providing support (plus educational materials)<br>T: Over 6 months – monthly monitoring	Psychological distress	Lower anxiety and depression mean HADS total score for intervention 6.01 v 8.20 control (P<.0001); HADS Depression subscale, Intervention 3.20 v 4.08 control (P=.0004); HADS Anxiety subscale Intervention 2.81 v 3.25

For peer review only

Source; Study design; Final quality rating	Setting and sample	Intervention and workforce	Primary outcomes (secondary outcomes)	Results
RCT<br>++	n=131<br><br>Cancer centres/university settings, USA	C: Educational materials alone, referred to oncology nurse upon evaluation if distressed significantly<br>W: Trained graduates monitoring telephone calls. Referral on to an oncology nurse where indicated.		control (P<.0001), at 6 months controlling for study entry levels.<br>No differences on other measures of psychological distress.
Lapid *et al.* 2007[42]<br><br>Secondary age group analysis of Rummans et al. 2006[67]<br><br>RCT<br>++	Adults aged 65+ newly diagnosed with advanced cancer<br>n=33<br><br>Cancer centre, USA	I: Multidisciplinary psychosocial QOL sessions<br>T: Eight 90 min sessions, 4 weeks after enrollment<br>C: Standard care (regular outpatient visits with oncologist and allied health care providers)<br>W: Led by psychiatrist or psychologist and co-facilitated by a nurse, physical therapist, chaplain or social worker. Leaders trained in materials and observed sessions.	Quality of life	Higher overall QOL intervention group scores throughout the study, not significant.<br>Higher QOL scores at week 4 intervention v control (79.3 vs 62.9, P=.0461)<br>Improvement in QOL scores for intervention at week 4 and 8 compared to older control group.
Mantovani *et al.* 1996[29]<br><br>RCT<br>++	Adults aged 65+ with cancer<br>n=72<br><br>Inpatient setting at medical oncology clinic, Italy	I1. Emotional and practical support from volunteers and I2. with structured psychotherapy<br>T: I2: Weekly sessions of 1 hour for 6 months<br>C: Pharmacological only<br>W: Trained volunteers basic = 40 hrs/6 months, another 40 hrs/6 months practical and further personal training.	Quality of life	Non-significant between group differences in functional status/physical symptom improvements over time : Karnofsky's Performance Status Scale (F=9.90, 2 df, P<.001). No differences on Spitzer's QOL Index or Functional Living Index - within/between groups<br>Significant between group differences: State-Trait Anxiety Inventory control significantly worsened and intervention groups significantly improved (I1 improved more than I2) (F=4.50, df 2, P<.05).<br>Beck Depression Inventory: control group unchanged, both intervention groups improved (F=229.66, 2df, P< .01).
Sajid *et al.* 2016[43]<br><br>RCT (pilot)<br>++	Men aged 70+ with prostate cancer and hormone therapy<br>n=19<br><br>Two medical oncology clinics, USA	I1. EXCAP (home based walking and resistance intervention)<br>I2. Technology mediated walking and resistance intervention using Wi-Fit<br>T: One face to face session then 6-12 weeks home based<br>C: Usual care<br>W: Trained exercise physiologist	Functional and aerobic<br><br>(Skeletal muscle and muscular mass measure; handgrip strength; chest repetition test; DEXA scan)	EXCAP intervention arm higher rate of change in steps per day at each follow up (+2720 steps) (P < 0.01) compared to control (+97 steps) and Wii-fit arm (+382 non- significant.)<br>EXCAP arm had a 2.3 point change in physical battery score after 12 weeks, compared to 0.6 points in the Wii-Fit arm and −0.5 points in the usual-care arm.<br>No other significant differences in outcomes.
Suh *et al.* 2013[39]<br><br>RCT<br>+++	Adults aged 65+ completed active treatment for gastro-intestinal cancers<br>n=63<br><br>Cancer centre, South Korea	I: 8 weeks of Qi exercise and 1 hour face-to-face counselling on physical and psychological factors<br>T: 8 weeks<br>C: Usual care<br>W: Two Qi exercise trainers, APNs	Physical activity<br><br>(Body mass index; body weight; nutritional status; symptom experience; self-efficacy; self-esteem)	Physical activity increased in both groups, extent of increase greater in intervention group (P=.005). Difference in amount of exercise over time between groups (P=.002).<br>No between group difference in BMI.<br>Nutritional status in both groups improved over time. The degree of reduction, however, was significantly larger in intervention group (P=.048), and same in interaction between group and time.<br>Both group and interaction factors significant positive difference in symptom experience, health promotion and Self-esteem for intervention.
Yagli and Ulger 2015[36]<br><br>Controlled before and after study<br>++	Women age 65-70 6 months after chemotherapy for breast cancer<br>n=20<br><br>Department of	I: 8 sessions of 1 hr yoga classes<br>T: 8 weeks<br>C: Exercise programme for 8 weeks<br>W: Existing physiotherapist (yoga teacher)	Quality of life; depression levels; levels of pain, fatigue and sleep quality	All patients' QOL scores improved pre to post yoga and exercise interventions.<br>Total scores and some sub categories of the Nottingham Health Profile showed significant difference in favor of the yoga group (P<.05) but not on energy level and pain where no differences.<br>Significant better fatigue and sleep quality in yoga group post intervention (P<.05).

For peer review only

Source; Study design; Final quality rating	Setting and sample	Intervention and workforce	Primary outcomes (secondary outcomes)	Results
	physiotherapy and rehabilitation, Turkey			
**Communication between patients and health care professionals**				
Devik, Hellzen and Enmarker 2015[23]<br><br>Qualitative<br>++	Adults aged 65+ with advanced cancer<br>n=9<br><br>Patients homes in rural Norway	I: Qualitative study of home nursing care to advanced cancer patients in rural locations<br>C: n/a<br>W: District nurses in normal role	Patient experience	Importance of nurses having a person-centred manner.<br>Ability to show a genuine and empathic interest in the patients.<br>Technical skills or special competences less discussed than personal qualities, such as having a sense of humour or generosity.<br>Good listening and communication skills.
van Weert *et al.* 2011[21]<br><br>RCT (cluster)<br>++++	Adults aged 65+ with cancer receiving chemotherapy<br>n=210<br><br>Staff: oncology trained nurses n=77<br><br>12 wards of 10 hospitals, Netherlands	I: Workforce: communication skills training in delivery of chemotherapy education to patients<br>T: 3 month implementation<br>C: Nurses continued to provide patient education as usual<br>W: Nursing and specialised oncology nursing roles.	Effects on quality of staff communication; effects on content of the consultation<br><br>(Patient recall of information)	Significant improvement in discussing realistic expectations C: -0.20; I: 0.45 (total between group difference 0.65) (P<.01).<br>Significant decrease in rehabilitation information pre to post change C:0.08, I: -0.38 (total between group difference -0.45) (P<.01).<br>No significant changes in categories treatment-related information and coping information.<br>Non sig: intervention group showed significant decrease in number of items discussed.<br>Less history taking pre to post (C: 1.83; I: -2.33; between group difference -4.17; p<.001) and less talking about all different side effects pre to post change (C: 1.98; I: -5.71; total 7.68; P<.001).<br>Patients in intervention asked more questions (M= 10.76) than control (M= 6.69; P<.05).<br>Marginal significant for intervention group: Proportion recall of recommendations post v pre. (C: -3.34; I: 6.39; Total: 9.73; P<.10).
Yeom and Heidrich 2013[24]<br><br>Observational<br>++	190 women at least one year post breast cancer diagnosis n=190<br><br>Community, an oncology clinic, and a state tumor registry, USA	I: Symptom management (IRIS): counselling interview and telephone follow up on symptom management<br>T: 8 week intervention with 16 week follow up point in the RCT.<br>C: Wait-list control subjects offered intervention after 16-week follow-up assessment<br>W: Advanced practice nurses	Negative beliefs about symptom management<br><br>(Quality of lIfe; purpose in life; positive relations with others)	Significant direct effects on Symptom Management Beliefs Questionnaire (SMBQ) (P<.00) and Communication Attitudes Questionnaire (P .012) on Communication Difficulties Questionnaire<br>Communication Difficulties significant direct, negative effects on all four dimensions of quality of life<br>Significant total effects of SMBQ on MCS (mental quality of life) (P=.001) and Purpose on Life (PL) and Personal Relations (PR) (P<.001) but not Physical component (PCS) .SMBQ predicted lower levels of quality of life in three of four dimensions.<br>None of the four indirect effects of SMBQ on quality of life through CommD was significant, indicating that CommD does not mediate the effects of SMBQ on quality of life<br>The total effects of CommA on four quality of life measures were not significant. However, the indirect effects for MCS (P=.05), PIL (P<.05) and PR (P<.05) through CommD were significant, indicating that CommD mediates the effects of CommA on MCS, PIL and PR.

Quality ratings: High ++++; Moderate +++; Low ++; Very low + Intervention/workforce description: I= Intervention; T= Timepoint; C= Control group; W: Workforce involved

For peer review only

Funding statement
This work was funded by Macmillan Cancer Support to support the work of the Expert Reference Group for the Older Person with Cancer. The views expressed are those of the authors and do not necessarily reflect those of Macmillan Cancer Support.

Contributors: JB, GL, PG and TW were responsible for the systematic review design. GL was responsible for data collection. JB, GL and PG were responsible for data extraction and appraising studies. JB, GL, PG and TW were responsible for data analysis and interpretation. All authors contributed in drafting the manuscript. JB is responsible for the overall content as the corresponding author.

None of the named authors has a conflict of interest in relation to this work or its publication.

Data sharing statement No additional unpublished data are available.

Reporting Guidelines: Prisma checklist (supplementary file 3)

For peer review only

REFERENCES

1. Yancik R, Ries LAG. Cancer in older persons: an international issue in an aging world. Seminars in Oncology;**31**(2):128-36.

2. Coleman MP, Forman D, Bryant H, et al. Cancer survival in Australia, Canada, Denmark, Norway, Sweden, and the UK, 1995–2007 (the International Cancer Benchmarking Partnership): an analysis of population-based cancer registry data. The Lancet;**377**(9760):127-38.

3. International Society of Geriatric Oncology. SIOG 10 Priorities Initiative. SIOG, 2011. http://www.siog.org/content/siog-10-priorities-initiative. (Accessed 24.03.2017)

4. Lynch MP, DeDonato DM, Kutney-Lee A. Geriatric Oncology Program Development and Gero-Oncology Nursing. Seminars in Oncology Nursing 2016;**32**(1):44-54.

5. Prouse J, Phillips J. Care of older people living with cancer: The role of the specialist nurse and alied health profesionals. Cancer Forum 2013;**37**(3):226-29.

6. Bridges J, Hughes J, Farrington N, et al. Cancer treatment decision-making processes for older patients with complex needs: a qualitative study. BMJ Open 2015;**5**(12):e009674.

7. Balducci L, Extermann M. Management of cancer in the older person: a practical approach. The Oncologist 2000;**5**(3):224-37.

8. Caillet P, Laurent M, Bastuji-Garin S, et al. Optimal management of elderly cancer patients: usefulness of the Comprehensive Geriatric Assessment. Clinical Interventions In Aging 2014;**9**:1645-60.

9. Expert Reference Group for the Older Person with Cancer. Older people living with cancer: designing the future health care workforce. Macmillan Cancer Support, 2016. http://sogweb-prod.neox24.ch/files/public/workforce-report.pdf (Accessed 24.03.2017)

10. Bridges J, Wengström Y, Bailey DE. Educational preparation of nurses caring for older people with cancer: an international perspective. Seminars in Oncology Nursing 2015;**32**(1):16-23.

11. Independent Cancer Taskforce. Achieving world-class cancer outcomes. A strategy for England 2015-2020. https://www.cancerresearchuk.org/sites/default/files/achieving_world-class_cancer_outcomes_-_a_strategy_for_england_2015-2020.pdf (Accessed 24.03.2017), 2015.

12. Lyman GH. Caring for the elderly cancer patient: training the next generation of oncologists. Journal Of Oncology Practice / American Society Of Clinical Oncology 2008;**4**(4):193-94.

13. Rao AV, Hurria A, Kimmick G, et al. Geriatric Oncology: Past, Present, Future. Journal of Oncology Practice 2008;**4**(4):190-92.

14. Maggiore RJ, Gorawara-Bhat R, Levine SK, et al. Perceptions, attitudes, and experiences of hematology/oncology fellows toward incorporating geriatrics in their training. Journal of Geriatric Oncology 2014;**5**(1):106-15.

15. Foubert J, Faithfull S. Education in Europe: Are cancer nurses ready for the future? Journal of BUON 2006;**11**(3):281-84.

16. McMillan S, Grodzicki BK, Shahrokni A. Building a Comprehensive Educational Model for Nurse Practitioner Students in Geriatric Oncology. Journal of the American Geriatrics Society 2016;**64**:S180-S80.

17. Bridges J, Lucas G, Wiseman T, et al. Preparedness of UK workforce to deliver cancer care to older people: summary report from a scoping review. University of Southampton, 2016. http://eprints.soton.ac.uk/401192/ (Accessed 24.03.2017).

18. Guyatt GH, Oxman AD, Vist GE, et al. GRADE: an emerging consensus on rating quality of evidence and strength of recommendations. BMJ: British Medical Journal 2008;**336**(7650):924.

19. Moher D, Liberati A, Tetzlaff J, et al. Preferred Reporting Items for Systematic Reviews and Meta-Analyses: The PRISMA Statement. PLOS Medicine 2009;**6**(7):e1000097.

20. Popay J, Roberts H, Sowden A, et al. Guidance on the conduct of narrative synthesis in systematic reviews. A product from the ESRC methods programme Version 1.: ESRC, 2006.

For peer review only

21. van Weert JC, Jansen J, Spreeuwenberg PM, et al. Effects of communication skills training and a Question Prompt Sheet to improve communication with older cancer patients: a randomized controlled trial. Critical reviews in oncology/hematology 2011; 80(1), 145-59.

22. Herr K, Titler M, Fine PG, et al. The effect of a translating research into practice (TRIP)--cancer intervention on cancer pain management in older adults in hospice. Pain Medicine (Malden, Mass) 2012; 13(8), 1004-17..

23. Devik SA, Hellzen O, Enmarker I. "Picking up the pieces" - Meanings of receiving home nursing care when being old and living with advanced cancer in a rural area. International Journal Of Qualitative Studies On Health And Well-Being 2015;**10**:28382-82.

24. Yeom HE, Heidrich SM. Relationships between three beliefs as barriers to symptom management and quality of life in older breast cancer survivors. Oncology nursing forum 2013; 40(3), E108-18.

25. Basu M, Linebarger J, Gabram SGA, et al. The effect of nurse navigation on timeliness of breast cancer care at an academic comprehensive cancer center. Cancer 2013;**119**(14):2524-31.

26. Fann JR, Fan MY, Unützer J. Improving primary care for older adults with cancer and depression. Journal of General Internal Medicine 2009; 24 Suppl 2.

27. Goodwin JS, Satish S, Anderson ET, et al. Effect of nurse case management on the treatment of older women with breast cancer. Journal of the American Geriatrics Society 2003; 51(9), 1252-9.

28. Kornblith AB, Dowell JM, Herndon JE, et al. Telephone monitoring of distress in patients aged 65 years or older with advanced stage cancer: a cancer and leukemia group B study. Cancer 2006; 107(11), 2706-14.

29. Mantovani G, Astara G, Lampis B, et al. Impact of psychosocial intervention on the quality of life of elderly cancer patients. Psycho-oncology 1996; 5(2), 127-35.

30. Hempenius L, Slaets JPJ, Asselt D, et al. Outcomes of a Geriatric Liaison Intervention to Prevent the Development of Postoperative Delirium in Frail Elderly Cancer Patients: Report on a Multicentre, Randomized, Controlled Trial. PloS One 2013; 8(6), e64834.

31. Rao AV, Hsieh F, Feussner JR. Geriatric evaluation and management units in the care of the frail elderly cancer patient. Journals of Gerontology: Series A, Biological Sciences and Medical Sciences, 2005; 60A(6), 798-803.

32. Somana-Ehrminger S, Dabakuyo TS, Manckoundia P, et al. Influence of geriatric oncology consultation on the management of breast cancer in older women: a French population-based study. Geriatrics & gerontology international 2015;**15**(1):111-9.

33. Deliens C, Deliens G, Filleul O, et al. Drugs prescribed for patients hospitalized in a geriatric oncology unit: Potentially inappropriate medications and impact of a clinical pharmacist. Journal Of Geriatric Oncology 2016; 7(6), 463-470.

34. Bourdel-Marchasson I, Blanc-Bisson C, Doussau A, et al. Nutritional advice in older patients at risk of malnutrition during treatment for chemotherapy: a two-year randomized controlled trial. PLoS One 2014; 9(9), e108687.

35. Mandelblatt J, Traxler M, Lakin P, et al. A nurse practitioner intervention to increase breast and cervical cancer screening for poor, elderly black women. The Harlem Study Team. Journal of General Internal Medicine 1993; 8(4), 173-8.

36. Yagli NV, Ulger O. The effects of yoga on the quality of life and depression in elderly breast cancer patients. Complementary therapies in clinical practice 2015; 21(1), 7-10.

37. McCorkle R, Strumpf NE, Nuamah IF, et al. A specialized home care intervention improves survival among older post-surgical cancer patients. Journal of the American Geriatrics Society 2000;**48**(12):1707-13.

38. Heidrich SM, Brown RL, Egan JJ, et al. An individualized representational intervention to improve symptom management (IRIS) in older breast cancer survivors: three pilot studies. Oncol Nurs Forum 2009;**36**(3):E133-43.

39. Suh EE, Kim H, Kang J, et al. Outcomes of a culturally responsive health promotion program for elderly Korean survivors of gastrointestinal cancers: a randomized controlled trial. Geriatric Nursing 2013; 34(6), 445-52.

For peer review only

40. Chock MM, Lapid MI, Atherton PJ, et al. Impact of a structured multidisciplinary intervention on quality of life of older adults with advanced cancer. International Psychogeriatrics 2013; 25(12), 2077-86.

41. Johansson B, Holmberg L, Berglund G, et al. Reduced utilisation of specialist care among elderly cancer patients: A randomised study of a primary care intervention. European Journal of Cancer 2001; 37(17), 2161-8.

42. Lapid MI, Rummans TA, Brown PD, et al. Improving the quality of life of geriatric cancer patients with a structured multidisciplinary intervention: a randomized controlled trial. Palliative & supportive care 2007; 5(2), 107-14.

43. Sajid S, Dale W, Mustian K, et al. Novel physical activity interventions for older patients with prostate cancer on hormone therapy: A pilot randomized study. Journal of Geriatric Oncology 2016; 7(2), 71-80.

44. Kalsi T, Babic-Illman G, Ross PJ, et al. The impact of comprehensive geriatric assessment interventions on tolerance to chemotherapy in older people. Br J Cancer 2015;**112**(9):1435-44.

45. Craig P, Dieppe P, Macintyre S, et al. Developing and evaluating complex interventions: the new Medical Research Council guidance. BMJ 2008;**337**:979-83.

46. Bridges J, Simcock R. Meeting the workforce challenges for older people living with cancer. International journal of nursing studies;**65**:A1-A2.

47. Kagan SH. The Future of Gero-Oncology Nursing. Semin Oncol Nurs 2016;**32**(1):65-76.

48. Levenson D. Advanced practice nurses lengthen survival of elderly cancer patients. Rep Med Guidel Outcomes Res 2001;**12**(5):5-7.

49. Morgan B, Tarbi E. The Role of the Advanced Practice Nurse in Geriatric Oncology Care. Semin Oncol Nurs 2016;**32**(1):33-43.

50. Prouse J. Geriatric assessment. The role of the specialist nurse practitioner. Asia-Pacific Journal of Clinical Oncology 2009;**5**:A155.

51. Extermann M, Aapro M, Audisio R, et al. Main priorities for the development of geriatric oncology: a worldwide expert perspective. Journal of Geriatric Oncology 2011;**2**(4):270-73.

52. Gosney M. Contribution of the geriatrician to the management of cancer in older patients. European Journal of Cancer 2007;**43**(15):2153-60.

53. Magnuson A, Dale W, Mohile S. Models of Care in Geriatric Oncology. Current Geriatrics Reports 2014;**3**(3):182-89.

54. Puts M, Santos B, Hardt J, et al. An update on a systematic review of the use of geriatric assessment for older adults in oncology. Annals of Oncology 2013; 25(2), 307-15.

55. Hamaker ME, Schiphorst AH, ten Bokkel Huinink D, et al. The effect of a geriatric evaluation on treatment decisions for older cancer patients–a systematic review. Acta Oncologica 2014;**53**(3):289-96.

56. Extermann M, Hurria A. Comprehensive geriatric assessment for older patients with cancer. Journal of Clinical Oncology 2007;**25**(14):1824-31.

57. Lycke M, Pottel L, Boterberg T, et al. Integration of geriatric oncology in daily multidisciplinary cancer care: The time is now. European Journal of Cancer Care 2015;**24**(2):143-46.

58. Chapman AE, Swartz K, Schoppe J, et al. Development of a comprehensive multidisciplinary geriatric oncology center, the Thomas Jefferson University Experience. Journal Of Geriatric Oncology 2014;**5**(2):164-70.

59. Bouzereau V, Le Caer F, Guardiola E, et al. Experience of multidisciplinary assessment of elderly patients with cancer in a French general hospital during 1 year: a new model care study. Journal Of Geriatric Oncology 2013;**4**(4):394-401.

60. Terret C, Zulian GB, Naiem A, et al. Multidisciplinary approach to the geriatric oncology patient. Journal of Clinical Oncology 2007;**25**(14):1876-81.

61. Mbah O, Ford JG, Qiu M, et al. Mobilizing social support networks to improve cancer screening: The COACH randomized controlled trial study design. BMC Cancer 2015; 15(1), 907.

For peer review only

62. Fraser M. Thinking differently about the cancer workforce. 2016. http://www.nuffieldtrust.org.uk/blog/thinking-differently-about-cancer-workforce. (Accessed 24.03.2017)

63. Glajchen M. Education, Training, and Support Programs for Caregivers of Individuals with Cancer. In: Talley RC, McCorkle R, Baile WF, eds. Cancer Caregiving in the United States: Research, Practice, Policy: Rosalynn Carter Institute for Caregiving Caregiving: Research-Practice-Policy series. New York and Heidelberg: Springer, 2012:79-102.

64. Muss HB. Cancer in the Elderly: a Societal Perspective from the United States. Clin Oncol 2009;**21**(2):92-98.

65. Brodie H. Getting the skill mix right. Nursing Older People 2013;**25**(6):10-10.

66. Clark MM, Rummans TA, Atherton PJ, et al. Randomized Controlled Trial of Maintaining Quality of Life During Radiation Therapy for Advanced Cancer. Cancer 2013;**119**(4):880-87.

67. Rummans TA, Clark MM, Sloan JA, et al. Impacting quality of life for patients with advanced cancer with a structured multidisciplinary intervention: a randomized controlled trial. Journal of clinical oncology : official journal of the American Society of Clinical Oncology 2006;**24**(4):635-42.

For peer review only

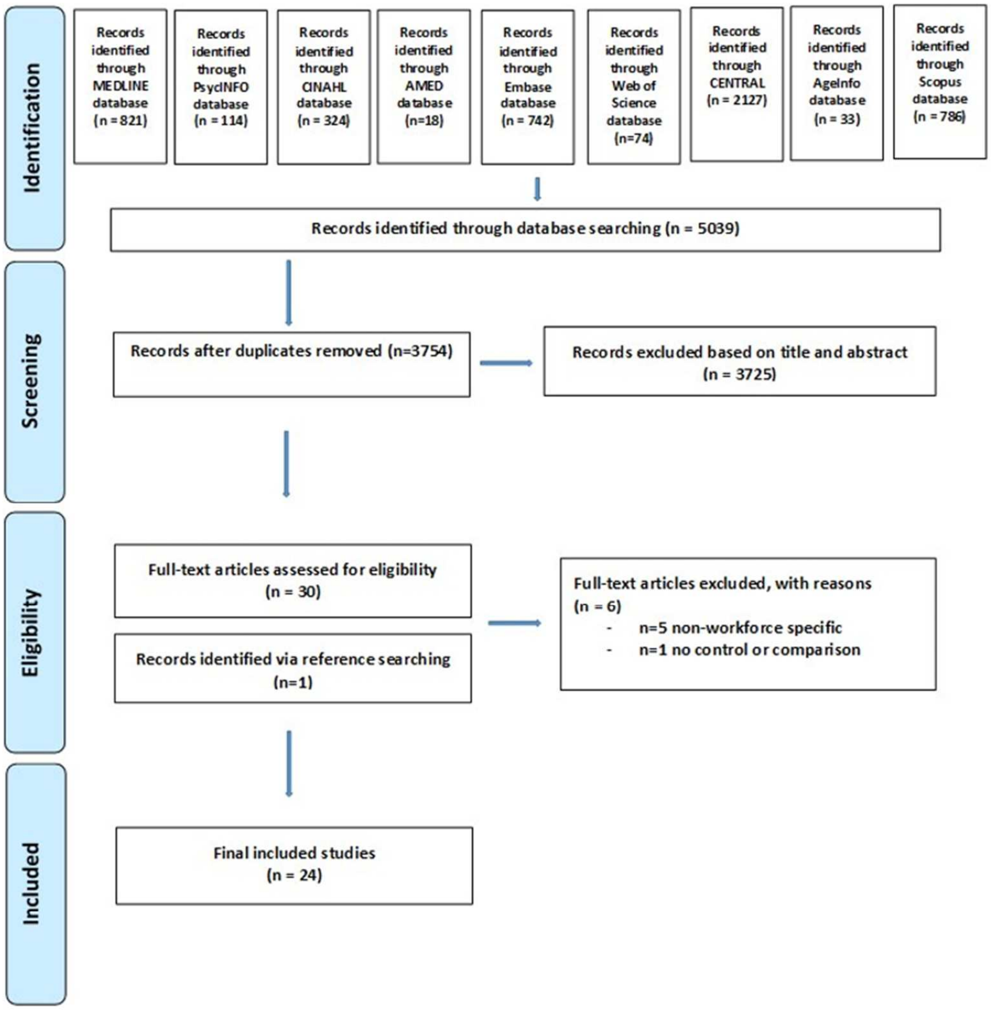
Identification
Screening
Eligibility
Included
Records identified through MEDLINE database (n = 821)
Records identified through PsycINFO database (n = 114)
Records identified through CINAHL database (n = 324)
Records identified through AMED database (n=18)
Records identified through Embase database (n = 742)
Records identified through Web of Science database (n=74)
Records identified through CENTRAL (n = 2127)
Records identified through AgeInfo database (n = 33)
Records identified through Scopus database (n = 786)
Records identified through database searching (n = 5039)
Records after duplicates removed (n=3754)
Records excluded based on title and abstract (n = 3725)
Full-text articles assessed for eligibility (n = 30)
Records identified via reference searching (n=1)
Full-text articles excluded, with reasons (n = 6)
- n=5 non-workforce specific
- n=1 no control or comparison
Final included studies (n = 24)

SUPPLEMENTARY FILE 1

Full search strategy

EBSCO HOST
Medline, CINHAL, PsycINFO, AMED: text terms and MeSH headings

1. TI Elderly OR AB Elderly
2. TI Geriatric* OR AB Geriatric*
3. TI “Older people” OR AB “Older people”
4. TI “Older patient*” OR AB “Older patient*”
5. TI “Older person” OR AB “Older person”
6. TI “Older adult*” OR AB “Older adult*”
7. MM Aged
8. MM Frail Elderly
9. **1 or 2 or 3 or 4 or 5 or 6 or 7 or 8**

10. TI Cancer OR AB Cancer
11. TI Oncolog* OR AB Oncolog*
12. MM Neoplasms
13. **10 or 11 or 12**

14. TI Workforce OR AB Workforce
15. TI “Health professionals” OR AB “Health professionals”
16. TI “Healthcare professionals” OR AB “Healthcare professionals”
17. TI “Health care professionals” OR AB “Health care professionals”
18. TI “Health personnel” OR AB “Health personnel”
19. TI “Healthcare personnel” OR AB “Healthcare personnel”
20. TI “Health care personnel” OR AB “Health care personnel”
21. TI “Medical personnel” OR AB “Medical personnel”
22. TI “Advanced Practice nurse” OR AB “Advanced Practice Nurse”
23. TI “Clinical nurse specialist” OR AB “clinical nurse specialist”
24. TI Geriatrician* OR AB Geriatrician*
25. TI Gerontologist* OR AB Gerontologist*
26. TI “Allied health professionals” OR AB “Allied health professionals”
27. TI Training
28. TI Educat*
29. TI “Skill mix” OR AB “Skill mix”
30. TI “Grade mix” OR AB “Grade mix”
31. TI “Staff development” OR AB “Staff development”
32. TI Staff* W1 level* OR AB Staff* W1 level*
33. TI Teamwork OR AB Teamwork
34. MM Health manpower
35. MM Health personnel
36. MM Attitude of Health personnel
37. MM Professional Competence
38. MM Staff development
39. MM Education, professional
40. MM Nurse’s role
41. MM Geriatric assessment

For peer review only

42. MM Health services for the aged
43. or/ 14-42
44. 9 AND 13 AND 43
45. English language filter

Embase (OVID)

1. elderly.ti,ab

2. geriatric.ti,ab

3. “older people”.ti,ab.

4. “older patient*”.ti,ab.

5. “older adult*”.ti,ab.

6. “older person”.ti,ab.

7. 1 or 2 or 3 or 4 or 5 or 6

8. cancer.ti,ab.

9. oncolog*.ti,ab.

1o. Neoplasm/

11. 8 or 9 or 10

12. workforce.ti,ab.

13. “health professionals”.ti,ab.

14. “healthcare professional*”.ti,ab.

15. “health care professional*”.ti,ab.

16. “health personnel”.ti,ab.

17. “healthcare personnel”.ti,ab.

18. “health care personnel”.ti,ab.

19. “medical personnel”.ti,ab.

20. “Advanced Practice nurse”.ti,ab.

21. “Clinical nurse specialist”.ti,ab.

22. Geriatrician*.ti,ab.

23. Gerontologist*.ti,ab.

24. “Allied health professionals”.ti,ab.

25. training.ti

For peer review only

26. educat*.ti

27. Skill Adj1 mix.ti,ab.

28. Grade Adj1 mix.ti,ab.

29. “staff development”.ti,ab.

30. “Workforce development”.ti,ab.

31. Staff adj1 level.ti,ab.

32. health care manpower/

33 professional competence/

34 vocational education/

35. health personnel attitude/

36. or/12-35

37. English.lg.

38. 7 AND 11 AND 36 AND 37

Scopus (Elsevier)

TITLE-ABS-KEY (cancer OR oncology OR neoplasm*) AND TITLE-ABS-KEY (elderly OR geriatric OR "older people" OR "older adult*" OR "older patient*" OR "older person") AND TITLE-ABS-KEY (workforce OR "health professional*" OR "health care professional*" OR “healthcare professional” OR "health personnel" OR "medical personnel" OR "advanced practice nurse" OR "clinical nurse specialist" OR geriatrician OR gerontologist OR "skill mix" OR "staff development" OR manpower)) AND (LIMIT-TO (LANGUAGE , "English"))

Web of Science (Thompson Reuters)

TITLE: (cancer OR oncolog* OR neoplasm*) AND TITLE: (elderly OR geriatric* OR "older people" OR "older person" OR "older adult*" OR "older patient*") AND TITLE: ("workforce" OR "health professional*" OR "healthcare professional*" OR “health care professional” OR "health personnel" OR "medical personnel" OR "advanced nurse practitioner" OR "clinical nurse specialist" OR geriatrician OR gerontologist OR "allied health professional*" OR training OR "educat*" OR "skill mix" OR "grade mix" OR "staff development")

Refined by: LANGUAGES: (ENGLISH)

Timespan=All years

CENTRAL

Elderly OR geriatric OR “older people” OR “Older adult*” OR “older patient*” OR “older person”

AND cancer OR oncolog* OR neoplasm*

In Title OR Abstract OR Key Words

Search for Trials

AgeInfo Cancer or Oncology in Title

For peer review only

SUPPLEMENTARY FILE 2 DATA EXTRACTION TABLE WITH EXAMPLE

STUDY AND AIM	STUDY DESIGN DATA COLLECTION MEASURES ANALYSIS	SETTING AND SAMPLE<br><br>COUNTRY CLINICAL SETTING PATIENT GROUPS STAFF GROUP	INTERVENTION	OUTCOMES	STAFF ROLE OR WORKFORCE DEVELOPMENT	RESULTS
Basu *et al.* (2013)<br><br>To evaluate timeliness in the period from breast cancer diagnosis to initial oncology consultation for a 9-month period prior to and following implementation of the nurse navigator.	Before and after study<br><br>Retrospective chart review of demographic data, tumour grade and stage, cancer treatment, appointment information, navigation status, time interval from diagnosis date to initial oncology appointment date.<br><br>Chi-square tests of independence used to compare by navigation group, and t tests used to compare continuous variables. Multiple linear	USA<br><br>One cancer centre<br><br>All women diagnosed within the institution with stage 0 to III breast cancer at initial presentation<br><br>176 F: 100 (57%) in prenavigation group) and 76 (43%) in post navigation group<br><br>Stratified by age <61 and 61+<br><br>In the 61+ age group there were 86 patients 52 in the pre navigation group 34 in the post	A dedicated breast cancer nurse navigator to assist patients with their care. The navigator's involvement begins when the patient is diagnosed and ends when the patient is appointed into the survivorship clinic.	The time interval from date of diagnosis to date of initial oncology appointment was measured in business days. Date of diagnosis was defined as the patient's first positive biopsy date. The date of initial oncology appointment was defined as the first date the patient had a consultation with a surgical, medical, or radiation oncologist. In a few cases a phone call	Collaborating with physicians and interdisciplinary team members to triage patients, coordinating patient care, educating patients and providing links to research and other relevant resources, and serving as the primary point of contact for patients and their families. The nurse navigator calls recently diagnosed patients within 24 hours of biopsy, meets patients at the initial visit, and assists patients in scheduling oncology appointments or any necessary	Overall, the median wait time was 10 days for all patients in the study and ranged from 1 to 32 days. The median wait time for patients in the prenavigation group was 11 days compared with 9 days for patients in the postnavigation group.<br><br>Because of interaction between navigation and age identified during the modeling process, stratified models presented for each of the 2 categories of age<br><br>For patients aged 61 years

SUPPLEMENTARY FILE 2 DATA EXTRACTION TABLE WITH EXAMPLE

STUDY AND AIM	STUDY DESIGN DATA COLLECTION MEASURES ANALYSIS	SETTING AND SAMPLE<br><br>COUNTRY CLINICAL SETTING PATIENT GROUPS STAFF GROUP	INTERVENTION	OUTCOMES	STAFF ROLE OR WORKFORCE DEVELOPMENT	RESULTS
	regression used to determine influence on time to consultation.	navigation group<br><br>Breast cancer nurse navigator		from the oncologist to discuss pathology results and treatment options was recorded as the consultation date if there was no other consultation before treatment.	diagnostic tests. Patients are then tracked during the course of care to ensure timeliness of appointments and therapies and to provide referrals to treatment and supportive services as needed.	and older, the median wait time was 4 days shorter for the navigation group (8 days versus 12 days).<br><br>In the 61 or older age group, navigation was a significant predictor of time to consultation, adjusting for demographic and clinical factors (P<.0002). In this adjusted model, the unstandardized beta coefficient for navigation was 24.9, indicating that with navigation, time to consultation decreased by an average of almost 5 days

For peer review only

Section/topic	#	Checklist item	Reported on page #
**TITLE**			
Title	1	Identify the report as a systematic review, meta-analysis, or both.	1
**ABSTRACT**			
Structured summary	2	Provide a structured summary including, as applicable: background; objectives; data sources; study eligibility criteria, participants, and interventions; study appraisal and synthesis methods; results; limitations; conclusions and implications of key findings; systematic review registration number.	2
**INTRODUCTION**			
Rationale	3	Describe the rationale for the review in the context of what is already known.	4
Objectives	4	Provide an explicit statement of questions being addressed with reference to participants, interventions, comparisons, outcomes, and study design (PICOS).	4
**METHODS**			
Protocol and registration	5	Indicate if a review protocol exists, if and where it can be accessed (e.g., Web address), and, if available, provide registration information including registration number.	7
Eligibility criteria	6	Specify study characteristics (e.g., PICOS, length of follow-up) and report characteristics (e.g., years considered, language, publication status) used as criteria for eligibility, giving rationale.	5-6
Information sources	7	Describe all information sources (e.g., databases with dates of coverage, contact with study authors to identify additional studies) in the search and date last searched.	4
Search	8	Present full electronic search strategy for at least one database, including any limits used, such that it could be repeated.	5
Study selection	9	State the process for selecting studies (i.e., screening, eligibility, included in systematic review, and, if applicable, included in the meta-analysis).	6
Data collection process	10	Describe method of data extraction from reports (e.g., piloted forms, independently, in duplicate) and any processes for obtaining and confirming data from investigators.	6
Data items	11	List and define all variables for which data were sought (e.g., PICOS, funding sources) and any assumptions and simplifications made.	4-6

For peer review only

Risk of bias in individual studies	12	Describe methods used for assessing risk of bias of individual studies (including specification of whether this was done at the study or outcome level), and how this information is to be used in any data synthesis.	6
Summary measures	13	State the principal summary measures (e.g., risk ratio, difference in means).	6
Synthesis of results	14	Describe the methods of handling data and combining results of studies, if done, including measures of consistency (e.g., I^2) for each meta-analysis.	6-7

Section/topic	#	Checklist item	Reported on page #
Risk of bias across studies	15	Specify any assessment of risk of bias that may affect the cumulative evidence (e.g., publication bias, selective reporting within studies).	7
Additional analyses	16	Describe methods of additional analyses (e.g., sensitivity or subgroup analyses, meta-regression), if done, indicating which were pre-specified.	N/A
**RESULTS**			
Study selection	17	Give numbers of studies screened, assessed for eligibility, and included in the review, with reasons for exclusions at each stage, ideally with a flow diagram.	Figure 1
Study characteristics	18	For each study, present characteristics for which data were extracted (e.g., study size, PICOS, follow-up period) and provide the citations.	Table 2
Risk of bias within studies	19	Present data on risk of bias of each study and, if available, any outcome level assessment (see item 12).	Table 2
Results of individual studies	20	For all outcomes considered (benefits or harms), present, for each study: (a) simple summary data for each intervention group (b) effect estimates and confidence intervals, ideally with a forest plot.	Table 2
Synthesis of results	21	Present results of each meta-analysis done, including confidence intervals and measures of consistency.	N/A
Risk of bias across studies	22	Present results of any assessment of risk of bias across studies (see Item 15).	7-11, Table 2
Additional analysis	23	Give results of additional analyses, if done (e.g., sensitivity or subgroup analyses, meta-regression [see Item 16]).	N/A
**DISCUSSION**			
Summary of evidence	24	Summarize the main findings including the strength of evidence for each main outcome; consider their relevance to key groups (e.g., healthcare providers, users, and policy makers).	11-12

For peer review only

Limitations	25	Discuss limitations at study and outcome level (e.g., risk of bias), and at review-level (e.g., incomplete retrieval of identified research, reporting bias).	3, 11-12
Conclusions	26	Provide a general interpretation of the results in the context of other evidence, and implications for future research.	11-12
**FUNDING**			
Funding	27	Describe sources of funding for the systematic review and other support (e.g., supply of data); role of funders for the systematic review.	19

For peer review only

BMJ Open

Workforce characteristics and interventions associated with high quality care and support to older people with cancer: a systematic review

Journal:	*BMJ Open*
Manuscript ID	bmjopen-2017-016127.R2
Article Type:	Research
Date Submitted by the Author:	07-Apr-2017
Complete List of Authors:	Bridges, Jackie; University of Southampton; NIHR CLAHRC Wessex<br>Lucas, Grace; University of Southampton, Faculty of Health Sciences<br>Wiseman, Theresa; Royal Marsden Hospital; University of Southampton, Faculty of Health Sciences<br>Griffiths, Peter; University of Southampton, Faculty of Health Sciences; NIHR CLAHRC Wessex
**Primary Subject Heading**:	Oncology
Secondary Subject Heading:	Geriatric medicine
Keywords:	Human resource management < HEALTH SERVICES ADMINISTRATION & MANAGEMENT, Adult oncology < ONCOLOGY, GERIATRIC MEDICINE

SCHOLARONE™
Manuscripts

For peer review only

TITLE PAGE

Workforce characteristics and interventions associated with high quality care and support to older people with cancer: a systematic review

Corresponding Author:
Professor Jackie Bridges
Professor of Older People's Care
Faculty of Health Sciences
University of Southampton
NIHR CLAHRC Wessex

Nightingale Building
University Road
Highfield Campus
Southampton SO17 1BJ
Tel.+44(0)23 8059 8282
Email jackie.bridges@soton.ac.uk

Grace Lucas
Faculty of Health Sciences
University of Southampton
Southampton
UK

Professor Theresa Wiseman
Faculty of Health Sciences
University of Southampton
Southampton
UK

Professor Peter Griffiths
Faculty of Health Sciences
University of Southampton
NIHR CLAHRC Wessex
Southampton
UK

Word count (excluding title page, abstract, references, figures and tables): 4286

For peer review only

Workforce characteristics and interventions associated with high quality care and support to older people with cancer: a systematic review

ABSTRACT

Objectives: To provide an overview of the evidence base on the effectiveness of workforce interventions for improving the outcomes for older people with cancer, as well as analysing key features of the workforce associated with those improvements.

Design: Systematic review

Methods: Relevant databases were searched for primary research, published in English, reporting on older people and cancer and the outcomes of interventions to improve workforce knowledge, attitudes or skills; involving a change in workforce composition and/or skill mix; and/or requiring significant workforce reconfiguration or new roles. Studies were also sought on associations between the composition and characteristics of the cancer care workforce and older people's outcomes. A narrative synthesis was conducted and supported by tabulation of key study data.

Results: Studies (n=24) included 4,555 patients aged 60+ from targeted cancer screening to end of life care. Interventions were diverse and two thirds of the studies were assessed as low quality. Only two studies directly targeted workforce knowledge and skills and only two studies addressed the nature of workforce features related to improved outcomes. Interventions focused on discrete groups of older people with specific needs offering guidance or psychological support were more effective than those broadly targeting survival outcomes. Advanced Practice Nursing roles, voluntary support roles and the involvement of geriatric teams provided some evidence of effectiveness.

Conclusions: An array of workforce interventions focus on improving outcomes for older people with cancer but these are diverse and thinly spread across the cancer journey. Higher quality and larger scale research that focuses on workforce features is now needed to guide developments in this field, and review findings indicate that interventions targeted at specific sub-groups of older people with complex needs, and that involve input from advanced practice nurses, geriatric teams and trained volunteers appear most promising.

Keywords: Neoplasms, health manpower, older people, Geriatric medicine, Oncology, Health services for the aged, Nurse's role, Aged, health personnel

STRENGTHS AND LIMITATIONS OF THIS STUDY

- This novel synthesis provides evidence of promising interventions targeted at delivering high quality care to older people with cancer.
- It is an international systematic review articulating the evidence base on workforce interventions that may support high quality cancer care to an expanding ageing population.
- The review is limited to those studies where the role of the workforce was explicitly articulated within an intervention; there may be other studies in which changes or adaptations to the workforce were tested but not reported and are not included here.
- The review only included items published in the English language.

For peer review only

Workforce characteristics and interventions associated with high quality care and support to older people with cancer: a systematic review

BACKGROUND

More than 60% of new cancers and more than 70% of cancer deaths occur in people over the age of 65 years in Europe and the United States.[1] Treatment outcomes for older cancer patients vary internationally[2] and this may be linked to the extent to which services and their associated workforce effectively meet the more complex needs associated with an ageing population.[3 4] Many older people have comorbidities and limitations which affects their cognitive and physical functioning, their risk of complications and their emotional wellbeing[5] all of which may affect cancer treatment tolerance and necessitate a modified treatment plan and relevant supportive care.[6] More comprehensive assessment and management has been recommended to optimise older cancer patients for treatment.[6-8] Furthermore, older people may value a range of outcomes beyond survival at any cost, including maintaining independence and being able to access information, emotional support and practical support both during and after treatment.[9] Health care workers who organize and deliver cancer care thus need knowledge of clinical and other issues which are common in old age, but also need to be adept with the skills and values to enable them to support the patient and family, develop treatment plans, deliver appropriate care, and help older people to achieve the quality of life that reflects what matters most to them as individuals.[10]

While the specific role of the health care workforce in ensuring optimal outcomes and quality of life for older cancer survivors and their families has been recognised, evidence suggests that there are variations internationally in the preparedness of the workforce to meet the needs of an ageing population.[9-16] Issues identified include deficits in the necessary education, knowledge, skills and attitudes; in staffing levels and skillmix; and in the development of roles, teams and services that meet older people's needs.[17] However, little is known about the features and characteristics of the workforce associated with better outcomes for older people with cancer, or about the relative effectiveness of workforce-focused interventions which are aimed at improving cancer care and outcomes for an ageing population. This systematic review therefore aims to inform developments in policy and practice by providing an overview of the evidence base on the effectiveness of workforce interventions for improving the outcomes for older people with cancer, as well as analysing key features of the workforce associated with those improvements.

METHODS

Systematic methods were used to guide searching, selection and analysis.[18] Searches for primary research evaluating workforce interventions for older people with cancer were undertaken in August

For peer review only

2016. Studies were identified by searching electronic databases, scanning reference lists of articles and by contacting study authors. A detailed search strategy was tested in MEDLINE (Table 1). The search was additionally tailored for database-specific subject headings and applied in: PsycINFO, Cumulative Index to Nursing and Allied Health Literature (CINAHL), Allied and Complementary Medicine Database (AMED), Embase, Web of Science, Cochrane Central Register of Controlled Trials (CENTRAL), AgeInfo and Scopus (Supplementary File 1). Searches were limited to the English language. No date limit was applied to ensure a comprehensive overview of developments in the field.

Table 1 Example of Search Strategy MEDLINE (EBSCOHOST)

**Concept 1**	**Concept 2**	**Concept 3**
1. TI Elderly OR AB Elderly	10. TI Cancer OR AB Cancer	14. TI Workforce OR AB Workforce
2. TI Geriatric* OR AB Geriatric*	11. TI Oncolog* OR AB Oncolog*	**15.** TI "Health professionals" OR AB "Health professionals"
3. TI "Older people" OR AB "Older people"	12. MM Neoplasms	**16.** TI "Healthcare professionals" OR AB "Healthcare professionals"
4. TI "Older patient*" OR AB "Older patient*"	13. 10 or 11 or 12	**17.** TI "Health care professionals" OR AB "Health care professionals"
5. TI "Older person" OR AB "Older person"		18. TI "Health personnel" OR AB "Health personnel"
6. TI "Older adult*" OR AB "Older adult*"		**19.** TI "Healthcare personnel" OR AB "Healthcare personnel"
7. MM Aged		**20.** TI "Health care personnel" OR AB "Health care personnel"
8. MM Frail Elderly		21. TI "Medical personnel" OR AB "Medical personnel"
9. 1 or 2 or 3 or 4 or 5 or 6 or 7 or 8		22. TI "Advanced Practice nurse" OR AB "Advanced Practice Nurse"
		23. TI "Clinical nurse specialist" OR AB "Clinical nurse specialist"
		24. TI Geriatrician* OR AB Geriatrician*
		25. TI Gerontologist* OR AB Gerontologist*
		26. TI "Allied health professionals" OR AB "Allied health professionals"
		27. TI Training
		28. TI Educat*
		29. TI "Skill mix" OR AB "Skill mix"
		30. TI "Grade mix" OR AB "Grade mix"
		31. TI "Staff development" OR AB "Staff development"
		32. TI Staff* W1 level* OR AB Staff* W1 level*
		33. TI Teamwork OR AB Teamwork

For peer review only

		34. MM Health manpower
		35. MM Health personnel
		36. MM Attitude of Health personnel
		37. MM Professional Competence
		38. MM Staff development
		39. MM Education, professional
		40. MM Nurse's role
		41. MM Geriatric assessment
		42. MM Health services for the aged
		43. or/ 14-42
		44. 9 AND 13 AND 43
		45. English language filter

Eligible study types included randomised controlled trials (RCTs), quasi-experimental or observational studies with a clearly defined workforce variable or intervention with a comparison between different exposure levels, and qualitative studies evaluating features of the workforce from the perspective of older people with cancer and where the role of the workforce forms a central part of the research question. We defined workforce based interventions as any intervention where the main mode of action was through a change in the composition, roles, knowledge, skills or attitudes of individuals or groups in a care delivery role, paid or unpaid, not including family or informal caregivers. Papers included reported on studies conducted with participants identified as older people (age 60+) at any stage in the cancer journey (from targeted screening through to end of life). Papers included reported on either:

- Outcomes of interventions to improve the knowledge, attitudes or skills of the workforce delivering cancer care and treatment to older people;
- Outcomes of interventions involving a change in the composition and/or skill mix of the workforce delivering cancer care for older people including (but not limited to) role substitution, new roles or adding specialist practitioners to the team;
- Outcomes of interventions routinely targeted at older people with cancer, which were reported to require significant workforce reconfiguration or the implementation of new roles; and/or
- Associations between the composition and characteristics of the cancer care workforce (including, but not limited to, staffing levels, skill mix, training, knowledge attitudes and skill) and outcomes for older people with cancer.

Studies reporting solely on drug, treatment or other therapeutic interventions (without specific focus on the workforce delivering those interventions) were not included.

For peer review only

Titles and abstracts from the searches were screened against the inclusion criteria by GL to exclude irrelevant papers. Five percent of titles/abstracts were also independently reviewed by another team member (JB, PG or TW) to confirm exclusion decisions. Full-text papers were retrieved for all papers that screened positively against inclusion criteria or about which a clear decision could not be taken (due to lack of information). Each full text paper was reviewed independently by two team members followed by a decision to include or exclude. These reviews were followed by further team discussion to finalise inclusion. The search and selection process is summarised in the PRISMA flow chart (Figure 1).[19]

Insert Figure 1 about here

Data on aim, design, setting, sample, intervention, outcome and results were extracted systematically from eligible papers using data extraction tables developed by the team (supplementary file 2). We adapted the GRADE system as used by Cochrane for rating evidence[18] to guide a broad assessment of individual study quality and thereby the contribution studies made to the review. Initial quality ratings based on study design were upgraded or downgraded depending on presence of factors considered to strengthen or weaken the evidence. Two members of the team independently reviewed all included papers. Discrepancies were discussed and ratings confirmed through discussions involving both raters and a third team member. No studies were excluded based on this assessment but lower quality studies were given less weight in the analysis.

Due to the heterogeneity of interventions and outcomes, a narrative analysis of study findings was merited.[20] Studies were grouped around the patient or service problems the interventions were targeting. Results were tabulated and the findings of effectiveness of individual interventions were plotted within these groups and used as the basis for an analysis of the strength of evidence of effectiveness across these groups and the field as a whole. We recorded and tabulated both the direction of differences between groups (where reported) and statistical significance of differences. Due to the number of different outcomes across the 24 studies, we report, within the results section, for the primary outcomes where there is evidence of significant differences between groups, rather than narrating the full set of results for each individual paper. A review protocol is available from the study team on request.

RESULTS

We identified 24 eligible published journal papers (23 quantitative and 1 qualitative study) covering 22 interventions and reporting on 4,555 patient participants age 60+ from targeted screening, through cancer diagnosis and treatment and beyond. All but one study were conducted in USA or

For peer review only

Europe. The studies report on 27 individual primary outcomes and 42 individual secondary outcomes (using a range of measures) across the studies corresponding to 41 different outcomes in total (n=38 of these were patient related outcomes and the other three outcomes were focused directly on the workforce). As detailed below and illustrated in Table 2, 17 studies were assessed as low or very low quality, with four studies rated as medium and three as high quality.

The point of the cancer journey each intervention was targeted at varied widely. Interventions ranged from targeted screening stage (n=1) and from diagnosis (n=4); to treatment phase/hospital stay (n=11); to those primarily focused on patients after the completion of their treatment (n=6); hospice care (n=1) or home care for advanced cancer patients (n=1). The majority of the interventions were limited to specific tumour types: 15 involved participants with a range of cancer types, but some involved more homogeneous populations: six were for breast cancer patients, one intervention targeted prostate patients, another involved those with gastrointestinal cancers, and one was aimed at breast and cervical screening.

Only two interventions were directly targeted at improving the knowledge, attitudes or skills of the workforce delivering cancer care and treatment to older people through training[21 22] and only two studies directly addressed the second objective of the review to assess the salient features of the cancer care workforce: one qualitative study considered the features of the nursing workforce which older patients felt were important in their care[23] and one study looked at the impact of healthcare professionals communication on participants' views about their symptom management.[24] The remaining studies reported on improving older people's outcomes via interventions involving a change in the workforce. In five interventions new roles were tested: nurse navigator,[25] depression care manager,[26] nurse case manager,[27] telephone support (trained graduates),[28] and social support volunteers.[29] In other studies, support from additional workforce members was provided to patients. Four studies reported on the increased involvement of a geriatrician or a geriatrics team,[27 30-32] one reported on the input of a clinical pharmacist,[33] and one study reported on the input of an additional dietician.[34] In two studies, a current staff member had a different function; in one study a nurse provided targeted cancer screening [35] and in another study a physiotherapist designed exercise and yoga programmes.[36] Three interventions used Advanced Practice Nurses – one in a homecare capacity[37] and two in counselling roles.[38 39] In three studies the role of multidisciplinary team members was highlighted.[40-42] In some papers, although a named member or members of the workforce were reported to have implemented or carried out the intervention, it was unclear as to the exact nature of their position. This was the case with two studies using exercise physiologists

For peer review only

where it could not be determined if they were existing or new staff members.[43],[39] Only seven studies referred to an explicit theoretical framework or model in intervention design.[21 22 24-26 38 39]

Because of the heterogeneity of studies retrieved (and the small number of studies that addressed the review's second objective), we reviewed evidence of the effectiveness of interventions by study type established through particular problems (related to older people with cancer) that the respective interventions were addressing and, subsequently, ways in which workforce requirements were being adapted to meet needs and improve outcomes related to these patient problems.. The results in Table 2 and set out below are displayed using these individual types.

Regular and timely access to care and treatment

Four studies focused on interventions targeted at the problem of systemic delays or inequitable access to treatment in the cancer journey for older people. They provide some promising evidence that providing additional support to some groups of older cancer patients can help them navigate the system and access treatment thereby improving the speed and efficacy of care. However, three of these papers provide only low quality evidence.

A high quality RCT reported that older women with breast cancer in the care of a nurse case manager acting as an educator, counsellor and coordinator were significantly more likely to see a radiation oncologist as part of initial evaluation, and to receive breast-conserving surgery and radiation therapy.[27] Further, the difference in receipt of appropriate treatment between women with characteristics associated with lower rates of appropriate treatment (75+, being unmarried, living alone, and being a member of an ethnic minority group) and their respective comparison groups were diminished or eliminated in the intervention group. An observational study reported that a breast cancer nurse navigator providing support and coordination of patient care from diagnosis until entry into survivorship clinic significantly shortened time to consultation for patients aged 61+ years.[25] A nurse practitioner role was used in a quasi-experimental study to improve screening rates for older Black women of low socioeconomic status by offering screening during a routine visit.[35] Nurse practitioner follow-up screening rates were significantly higher than baseline, compared with control group follow-up rates. A further study assessed the impact of a geriatrician consultation and treatment plan through an analysis of registry data of older breast cancer patients.[32] Patients who had a consultation had more co-morbidities and more advanced and aggressive tumours, were more likely to receive mastectomy and adjuvant therapy, and were less likely to be treated by breast-conserving surgery and adjuvant therapy.

For peer review only

Complications and specific problems from cancer treatment

Four studies reported the use of workforce members with specialist skills to address cancer treatment complications and impact on mortality and survival. None of the three low quality studies found any intervention effect on mortality rates, but the one high quality RCT found that specialised home care advanced practice nurses (used to enhance surgical recovery) increased two year survival for late stage cancer patients in the intervention group.[37]

Other lower quality studies in this group included evaluations of face-to-face counselling to address nutritional intake for patients treated with chemotherapy and at risk of malnutrition,[34] an intervention focused on the prevention of post-operative delirium with input from a geriatric team,[30] and comprehensive geriatric assessment (CGA) targeted at chemotherapy tolerance and toxicity.[44] The observational study evaluating CGA for older chemotherapy patients found that CGA patients were more likely to complete cancer treatment as planned but no significant differences were found in relation to mortality or other outcomes measures in relation to the interventions in any of these three studies.

Co-morbidities and complex health needs

The five studies reported here target the health issues that may accompany a cancer diagnosis, but also broader health problems that may not directly relate to the cancer. They highlight the importance of recognising and addressing these needs, although the range of outcomes and the variable quality of evidence (three studies of medium quality; two were low quality) make it difficult to draw firm conclusions about the best use of workforce support in this sizeable area.

A cluster RCT evaluating a hospice staff training programme on improving pain assessment and management did not find significant practice improvements or decreases in patient pain severity associated with the intervention.[22] In a different study, a secondary analysis of RCT data on the impact of a depression care manager providing education and support for older patients with depression found that intervention patients with a cancer diagnoses were twice as likely to experience a depression treatment response at 12 months compared to usual care.[26] Rao *et al.* also reviewed the outcomes for cancer patients from a wider RCT evaluating the impact of involving a geriatric team in the care of inpatient and outpatients diagnosed with frailty.[31] The inpatient intervention group showed significant improvements in bodily pain and mental health versus the usual inpatient care group but there was no impact on survival rates. There were no intervention effects on outpatients. An uncontrolled before and after study, reported that using a clinical pharmacist to identify patients' potentially inappropriate medications (PIMs) reduced the number of PIMs at discharge versus admission.[33] A low quality RCT reported that intensified primary healthcare

For peer review only - http://bmjopen.bmj.com/site/about/guidelines.xhtml

support significantly reduced the number of days in hospital for an intervention group of advanced cancer patients compared to patients receiving standard care.[41]

Quality of life, physical and psychological functioning

Eight studies focused on addressing quality of life (QOL) across its physical and psychological aspects. This group of interventions used a range of workforce members (often in therapeutic or supportive roles) from physiotherapists to advanced practice nurses to trained voluntary input, to address a range of factors underpinning quality of life. They showed mixed evidence of effectiveness. Seven of the studies in this group provided low quality evidence.

Three studies focused on physical functioning in particular. In an RCT with low recruitment rate and possible selection bias, exercise physicians provided Qi exercise training.[39] Both usual care and intervention participants increased their activity levels but the extent of the increase was significantly greater in the intervention group. The intervention also used advanced practice nurses delivering face-to-face counselling and significant improvements in symptom experience, self-efficacy and self-esteem were reported. A controlled before and after study compared the effect of yoga classes (with the input of a physiotherapist/yoga teacher) with a standard exercise programme.[36]

QOL scores after the program were better than before for both groups, but some QOL parameters improved more for those included in the yoga intervention. A pilot RCT with small sample and high dropout compared two exercise forms implemented by a physiologist (compared to usual care) and found significant activity increases for the group using a home-based walking and resistance intervention.[43]

Two similar interventions involved a multidisciplinary team approach for a range of quality of life domains, however both of these secondary analyses reported on very small samples sizes of older adults within wider QOL interventions. Lapid *et al.*[42] found in a secondary analysis of a small sample of patients in a wider RCT, that higher QOL scores were reported for older patients who received multidisciplinary emotional and practical support. However, in the study by Chock et al,[40] the authors did not find any lasting differences on QOL for older intervention participants against their younger counterparts, apart from an improvement in anger-hostility.

Advanced practice nurses were used in a symptom management intervention in the two pilot RCTs and the observational study reported by Heidrich *et al.*[38] Some evidence of effectiveness was reported for improving self-care and reducing symptom distress and duration, but there was no impact on QOL.

For peer review only - http://bmjopen.bmj.com/site/about/guidelines.xhtml

Two studies used trained volunteers to bolster psychological support. A secondary analysis of RCT data was used to evaluate the effect of using trained graduate support workers to provide initial distress monitoring to patients over the telephone. [28] Intervention patients had significantly lower anxiety and depression at six months than patients receiving educational materials alone. However, no other differences in psychological wellbeing were detected. Mantovani *et al.*[29] also used trained support volunteers to provide emotional and practical support. An RCT with small sample size was used to compare this support with pharmacological treatment alone, and further with the addition of psychotherapy. Significant improvements in anxiety and depression were reported for the groups receiving voluntary support and/or additional psychotherapy. However, there were no significant differences on other QOL measures.

Communication between health care professionals and older people with cancer

Three studies focused on addressing the communication needs of older people with cancer. One high quality study offered communication skills training to staff with varied success[21] and the other two low quality studies highlighted the importance of good communication as a prerequisite for cancer nurses related to improving older patients' quality of life.

A cluster RCT found that training nursing staff to improve chemotherapy patient education led to a significant, positive effect for 'discussing realistic expectations'.[21] Significantly less history taking was also observed pre to post in the intervention group, as well as less talking about all the possible side effects; both points of attention during training. No other significant effects were reported. Yeom and Heidrich[24] used a cross-sectional analysis of RCT data to report that communication difficulties with health professionals had significant direct, negative effects on QOL dimensions. Findings from a qualitative interview study highlighted the value to older cancer patients of nurses having a person-centred manner, with the ability to show a genuine and empathic interest in the patients and to make a connection with good listening and communication skills.[23]

DISCUSSION

This systematic review aimed to provide an overview of the evidence base on the effectiveness of workforce interventions for improving the outcomes for older people with cancer, as well as analysing key features of the workforce associated with those improvements. Findings reflect a range of ways in which the workforce has been adapted, expanded or trained to addressing older cancer patients multiple and divergent needs. The findings present a novel synthesis of the type of interventions being developed globally to address the broad question of how the workforce can support the improvement of older people's cancer outcomes. The approaches and the patient problems they are addressing are varied, including integrating the input of geriatric specialists into

For peer review only

cancer services, using advanced practice nurse roles to support patients, creating new roles to guide patients through the healthcare system and ensuring effective treatment, through to novel approaches using voluntary support, or trialling yoga or other exercise to improve older patients' quality of life.

While the included studies begin to provide evidence about how the workforce can be used to make a tangible difference to older cancer patients' physical and psychological outcomes, the diversity of interventions in the studies reviewed and the range of outcomes evaluated, limits generalisations on effectiveness. Further, the quality of evidence is generally low. Experimental designs were not consistently used and, when they were, their implementation was often hampered by poorer than expected recruitment, or conclusions drawn about outcomes for older patients were drawn from a secondary analysis of a wider dataset. In addition, as is common in the reporting of complex intervention evaluations, details of the intervention itself were often lacking.[45] There was inadequate reporting of the specific workforce contribution to the interventions and limited evidence to address the second objective of the review around the features of the cancer care workforce associated with better outcomes. In addition, while staff training was involved in half of the interventions reported, the details of how that training worked or could be improved was not detailed. Furthermore, although some innovative roles were set up, the rationale and detail of those roles was often poorly reported.

Despite these shortcomings, these findings do provide some promising insights into how the workforce may address the varied needs of older cancer patients, albeit with a dearth of evidence at the earlier and later stages of the cancer journey. Evidence has suggested that not all older people with cancer need the same input, and indeed age-related changes occur at different rates in different individuals and are not reflected in chronologic age.[7] Therefore, it is more productive to focus attention on those with complex problems.[46] The studies in this review appear to support the notion of targeted assistance to groups at particular risk of under-treatment. Review findings suggest that broader interventions aiming to improve survival outcomes are less successful, but studies did indicate the kind of support that could be put in place after treatment to deal with the specific complications and problems that older people might face. One intervention which did improve survival used advanced practice nurses in home-care support post-surgery.[37] Indeed, the role of advanced practice nurses in the future of older people's cancer care has been acknowledged elsewhere in the literature,[47-50] and this review indicates that this is a candidate role for exploration and further consideration.

For peer review only

The input of geriatric specialists who are able to assess and manage older patients and optimise patients for treatment was a significant feature of several studies reviewed and formal links and services are well established in some countries.[51-53] Findings from this review provide weak evidence of positive benefits from the input of geriatricians but it only included studies where the geriatrician's role was explicit in the intervention and where a comparison or control was featured. There are a number of other reviews reporting on specialist geriatric assessment and management for older cancer patients, and these have been able to draw firmer conclusions about the benefits of CGA with older cancer patients, although they all acknowledge the need for more definitive research.[54-56] Multidisciplinary approaches also emerged as a feature across the studies reviewed and the need to shape teams around the multiple needs of older people with cancer has been highlighted elsewhere, although evidence from this review is weak, again limited by the scale and quality of the research.[6 57-60]

Of further interest is the use of non-professionals in providing direct care services to older people with cancer, and roles such as these are relevant in the contexts of budgetary pressures and recruitment difficulties of key professional groups such as geriatricians and registered nurses.[17] The two studies reviewed suggested a positive impact on patient outcomes and align with a growing recognition of the non-clinical workforce (including carers and families) playing an essential role in older people's cancer care.[61-63] However, the low quality of the research again reduces confidence in these positive findings. A final point is that the studies identified for this review did not address the impact of staffing levels or skillmix on older cancer patients' outcomes. In addition, few mechanisms to develop the current workforce to prepare for and be supported to deliver high quality care to an ageing population were identified. In addition to the development and more definitive evaluation of new roles and practices, the future research agenda must address these important facets to ensure that, regardless of setting, all health care workers that older people with cancer encounter, are prepared for and adequately supported in their role.[64 65]

This review alone is insufficient to enable conclusions to be drawn about the workforce factors which prove most beneficial to older people's outcomes; further high quality RCTs are needed to assess the potential of possible interventions. Future research should build on the studies reviewed here to establish what workforce developments are needed to support this growing population throughout the cancer journey. The most promising interventions for further study target assistance to individuals with complex needs who are at particular risk of under-treatment, and of problems arising from cancer treatment or its impact. Our review indicates that the impact of multiprofessional teams, including geriatric physicians and advanced practice nurses, on patient

For peer review only

outcomes from survival to quality of life, would be worthwhile to evaluate more definitively, as would the contribution of trained volunteers.

Figure 1 Legend: PRISMA study selection flowchart

For peer review only

Table 2: Summary of studies included in review

Source; Study design; Final quality rating	Setting and sample	Intervention and workforce	Primary outcomes (secondary outcomes)	Results
**Regular and timely access to care and treatment**				
Basu *et al* 2013[25]<br><br>Observational: care control<br>++	Women aged 61+ with breast cancer<br>n=86<br><br>One cancer centre, USA	I: Patient Navigation: Support and coordination of patient care<br>T: From point of diagnosis to survivorship clinic<br>C: No navigation<br>W: Breast cancer nurse	Time from diagnosis to oncology appointment	Time to consultation decreased by 4.9 days (P=.0002)
Goodwin *et al.* 2003[27]<br><br>RCT<br>++++	Women aged 65+ newly diagnosed with breast cancer, n=335<br><br>13 community and two public hospitals, USA	I: Case management: nurse as educator, counsellor, advocate, and care coordinator<br>T: 12 months service<br>C: usual care [unclear]<br>W: Nurse case manager	Treatment received in 6 months after breast cancer diagnosis<br><br>(Patient satisfaction; arm function)	More intervention women saw radiation oncologist (36% vs 19.3%) (P=.006), received more breast-conserving surgery (28.6% vs 18.7%; P=.031) and radiation therapy (36.0% vs 19.0%; P=.003), had more breast reconstruction surgery (9.3% vs 2.6%, P=.054); reported choice in treatment (82.2% vs 69.9%, P=.020)<br>No differences between groups in percentages who saw an oncologist, discussed breast reconstruction, underwent complete surgical staging, or tissue sent for hormone receptor assay.
Mandelblatt *et al.* 1993[35]<br><br>Controlled before and after study<br>++	Women aged 65+ screening for breast or cervical cancer<br>n=673<br><br>Two public hospitals, USA	I: Screening intervention during routine visits<br>T: At scheduled appointments<br>C: Physician reminder system<br>W: Nurse practitioner	Annual screening rates for Pap tests and mammographies	Annual intervention site Pap test rate increased (17.8% to 56.9%), and mammographies (18.3% to 40%) compared to control site increase of Pap test rate from 11.8% to 18.2% and no change (18%) for mammography (P =.01).
Somana-Ehrminger *et al.* 2015[32]<br><br>Observational<br>++	Women aged 75+ with breast cancer<br>n=206<br><br>Breast and gynaecological cancer registry, France	I: Geriatrician referral and treatment plan<br>C: Patients with no geriatric oncology consultation<br>W:Geriatrician, dietician, psychologist, physical therapist or social worker	Independent impact of geriatric oncology consultation (GOC)	GOC patients more likely to receive mastectomy and adjuvant therapy (P <.0001); and less likely to be treated by breast-conserving surgery and adjuvant therapy (P =.003).
**Complications and specific problems of cancer treatment**				
Bourdel-Marchasson *et al.* 2013 [34]<br><br>RCT<br>++	Chemotherapy patients aged 70+ n=336<br><br>12 public and private settings, France	I: Face to face dietary counselling<br>T: 6 visits (3-6 months)<br>C: Usual care<br>W: Dietician	One year mortality<br><br>(Chemotherapy management; unplanned hospitalisation; 2year mortality)	Difference of 178kcal/day dietary intake in intervention group (P<.01).<br>No difference in other outcomes.
Hempenius *et al.* 2013[30]<br><br>RCT<br>++	Frail adults aged 65+, elective surgery for solid tumour<br>n=260<br><br>Teaching hospital and community hospital, Netherlands	I: Delirium prevention: assessment, monitoring and individualised treatment plan<br>T: During hospital stay<br>C: Usual care. Additional geriatric care provided on referral.<br>W: Geriatric team supervised by a geriatrician.	Incidence of post-operative delirium up to 10 days<br><br>(Severity of delirium; length of hospital stay; complications; mortality; care dependency; quality of life)	Significant difference in return to preoperative living situation (67.3% vs.79.1%, Odds ratio (OR): 1.84, 95% confidence interval (CI): 1.01-3.37).<br>No significant difference in other outcomes.
Kalsi *et al.* 2015[44]	Adults aged 70+ with cancer	I: Geriatrician comprehensive geriatric assessment and intervention plan (CGA) for identified need.	CGA impact on chemotherapy tolerance and toxicity; rate of planned completion of	Intervention more likely to complete planned cancer treatment (33.8% vs 11.4% OR 4.14 (95% CI: 1.50–11.42), P = 0.006) and fewer required

For peer review only

Source; Study design; Final quality rating	Setting and sample	Intervention and workforce	Primary outcomes (secondary outcomes)	Results
Observational<br>++	n=135<br><br>One hospital, UK	T: pre chemotherapy and further support as needed<br>C: Standard oncology care<br>W: Geriatrician	cancer<br><br>(Treatment modifications; early treatment discontinuation; death at six months)	treatment modifications 43.1% vs 68.6%, (OR 0.34 (95% CI: 0.16–0.73), P=0.006).<br>Lower toxicity rate in intervention group (43.8% vs 52.9%, P =.292).<br>No differences in death rates.
McCorkle *et al.* 2000[37]<br><br>RCT<br>++++	Adults aged 60+ post-surgical cancer patients<br>n=375<br><br>Comprehensive cancer centre, USA	I: Specialized home care advanced practice nurses (APNs) assess and monitor physical, emotional, and functional status of patients, provide direct care, access services and other resources from the community, and provide teaching, counseling and support during recovery.<br>T: 4 weeks with three home visits and five telephone contacts<br>C: Usual follow-up care in an ambulatory setting and routine outpatient follow-up<br>W: Advanced practice nurses (APNs)	Length of survival<br><br>(Depressive symptoms; symptom distress; functional status)	Late stage patients, improved 2 yr survival in intervention group: 66.7% v 39.6% (P < .05). No difference for early stage patients<br>Risk of death higher for control (adjusted hazard ratio 2.04; CI, 1.33-3.12; P= .001) compared with intervention group.<br>No differences between groups in other outcomes.
**Comorbidities and complex health needs**				
Deliens *et al.* 2016[33]<br><br>Uncontrolled before and after study<br>+	Adults aged 70+ with cancer (non-haematological) hospitalised<br>n=91<br><br>Geriatric oncology unit, tertiary hospital, Belgium	I: Medication review: Identification of PIMS and drug interactions<br>T: From point of admission and during hospitalisation<br>C: Before to after<br>W: Clinical pharmacist	Potentially inappropriate medications (PIMS) using START (screening tool to alert doctors to right treatment) and STOPP (screening tool of older person's potentially inappropriate prescriptions) criteria.<br><br>(Drug to drug interactions)	START criteria: 41 PIMS for 31 patients (34%) on hospital admission compared to 7 PIMS for 6 persons (7%) at discharge.<br>STOPP criteria: 50 PIMs for 29 patients (32%) at admission compared to 16 PIMS for 14 persons (16%) at discharge.
Fann, Fan and Unutzer 2009[26]<br><br>RCT<br>+++	Adults aged 60+ diagnosis of non-skin cancer and major depression or dysthymia<br>n=215<br><br>18 primary care clinics at 8 diverse health care organisations, USA	I: Depression management: education, 'behavioural activation', treatment support.<br>T: Up to 12 months. Followed up usual care 12 months more.<br>C: Usual care: received routinely available depression treatment<br>W: Depression care manager (nurse or clinical psychologist) collaborative with primary care.	Depression treatment response<br><br>(Health related quality of life (QOL); health-related impairments: work, family, social functioning; patient satisfaction**)**	Intervention twice as likely to experience a depression treatment response at 12 months than control (39% vs. 20%; P = .029) and at 18 months (38% vs. 16%; P = .012).<br>Remission rates higher in intervention group v control at 6 months (32% v 16% P = .006) and 12 months (22% v 9%, P = .031).<br>Less functional impairment at 12 months (P=.011) and greater QOL (P=.039).
Herr *et al.* 2012[22]<br><br>RCT (cluster)<br>+++	Adults aged 65+ with cancer receiving hospice care<br>n=738<br><br>Staff: Nurses (n=383 pre, n=415 post) and physicians (n=16)<br><br>16 hospices, USA	I: Workforce: to promote adoption of evidence-based pain practices. Included: training, assessment of data, champion input, senior leadership engagement.<br>T: Engagement phase 5 months, 12 month intervention.<br>C: Hospices received clinical practice guidelines<br>W: 3 days training. Selection of local pain facilitators, nurse and physician champions, Grant Expert Nurse input, Nurse and Physician Champion	Workforce: adoption of evidence-based cancer pain practices<br><br>(Pain severity)	No significant difference in improvement on Cancer Pain Practice Index between intervention and control.<br>Decrease in patient pain severity from pre to post in intervention group greater (p=.1032)
Johansson *et al.* 2001[41]<br><br>RCT	Adults aged 70+ newly diagnosed with prostate, GI or breast cancer<br>n=161	I: Intensified primary healthcare. Individual support: nurse support, nutritional support, and individual psychological support.<br>T: Starting from diagnosis	Utilisation of specialist care	Mean days of hospitalization for older intervention patients than control (3.8 v 8.9, P<.01).<br>4 of 82 older intervention patients admitted compared with 12 of 79 older control patients (P<.05).

For peer review only

Source; Study design; Final quality rating	Setting and sample	Intervention and workforce	Primary outcomes (secondary outcomes)	Results
++	Primary healthcare services, Sweden<br><br>(Other participants reported: n=255 under 70 years)	C: Standard care + group rehabilitation<br>W: Homecare nurse, dietician and psychologist.<br>GPs and nurses trained in pain, nausea and diet, in final stage life.		10 out of 82 made acute visits to outpatient clinics compared to 22 of 79 in control group (P<.05).
Rao *et al.* 2005[31]<br><br>RCT<br>+++	Adults aged 65+ with cancer, frail and hospitalized<br>n=99<br><br>11 medical centres, USA	I: Assessment and monitoring by geriatric team. 1. geriatric inpatient +usual outpatient; 2. Usual inpatient +geriatric outpatient; 3. Geriatric inpatient and outpatient<br>T: 1 year study<br>C: Usual care: all hospital services except from geriatric team<br>W: Core team: geriatric medicine attending physician, fellow or intern, a nurse practitioner, social worker.	Survival ; Health related quality of life<br><br>(Functional status; physical performance)	No difference: in survival for the cancer patients regardless of treatment group<br>Significant effect of geriatric inpatient care v usual inpatient care: mean change in score from randomization to discharge: bodily pain (28.7 v 10.1) P=.09; emotional limitation (29.3 v 2.7) P=.01. Effect on bodily pain sustained at 1 year (37.6 v 9.9).<br>No effect of geriatric outpatient care on any of the QOL parameters.<br>No effect of either inpatient or outpatient geriatric care on the functional status of cancer patients.
**Quality of life, physical and psychological functioning**				
Chock *et al.* 2013[40] (Secondary analysis of Clark et al. 2013)[66]<br><br>RCT<br>++	Adults aged 65+ with advanced cancer treated with radiotherapy<br>n=16<br><br>Cancer centre, USA<br><br>(Other participants reported: n=38 under 65 years)	I: QOL intervention with telephone follow up: Physical therapy, education, cognitive behavioural interventions, discussion and support, spiritual reflection and relaxation training<br>T: 6 sessions 90 mins, 2-4 weeks and 10 brief structured telephone sessions<br>C: Standard care<br>W: Multidisciplinary (including physical therapist, clinical psychologist, APN, Chaplain)	Quality of life; mood	Significant difference at week 4 only in mean overall QOL older v younger adults (74.4 vs 62.9, P=.040).<br>No significant difference in<br>Significantly lower Anger-Hostility dimension of mood measure at all weeks for older v younger patients. Week 4: 95.0 vs 86.4, P=.028; wk 27: 92.2 v 84.2, P=.027) and wk 52 (96.3 v 85.9, P=.005).<br>No other significant differences.
Heidrich *et al.* 2009[38]<br><br>Two pilot RCTs and one observational study<br>++	Women aged 65+, 1 year post diagnosis of non-metastatic breast cancer<br>n=82 (total)<br><br>Oncology clinics, cancer centre, USA	I: Pilot 1 - Symptom management (IRIS): counselling interview and telephone follow up on symptom management at 4 weeks; Pilot 2- Addition of four biweekly telephone reinforcement sessions; Pilot 3 - Intervention by phone only<br>C: 1 -usual care; 2-delayed IRIS (waitlist) control; 3 - no control: (IRIS Group only)<br>T: 4 weeks (pilot 1)<br>W: Advanced practice nurses	Feasibility, acceptability<br><br>(Symptom distress; symptom management; quality of life; mood; barriers to symptom management; communication difficulty)	Feasibility: Across all studies, 76% of eligible women participated, 95% completed the study, 88% reported the study was helpful, and 91% were satisfied with the study.<br>**Pilot 1:** No significant difference in symptom distress. Significant decrease in distress baseline to follow up in intervention group; Significantly more women in intervention reported changing self-care of symptoms (P<.05); No significant QOL differences.<br>**Pilot 2:** Significant less symptom duration compared to control at 8 weeks (P <.01). At 16 weeks, intervention group more likely to have talked to healthcare provider, begun new symptom treatment, and changed self-care symptoms (P<.05). No significant QOL differences. Negative attitudes from healthcare providers reported by 5%–20% of women and communication difficulties by 5%–45% of women.<br>**Pilot 3:** No significant differences (no control) baseline to eight weeks. Symptom interference decreased (and negative mood from symptoms). Symptom duration interference, and negative mood from symptoms decreased. No QOL change.
Kornblith *et al.* 2006[28]	Adults aged 65+ with breast, colon or prostate cancer	I: Telephone monitoring of distress providing support (plus educational materials)<br>T: Over 6 months – monthly monitoring	Psychological distress	Lower anxiety and depression mean HADS total score for intervention 6.01 v 8.20 control (P<.0001); HADS Depression subscale, Intervention 3.20 v 4.08 control (P=.0004); HADS Anxiety subscale Intervention 2.81 v 3.25

For peer review only

Source; Study design; Final quality rating	Setting and sample	Intervention and workforce	Primary outcomes (secondary outcomes)	Results
RCT<br>++	n=131<br><br>Cancer centres/university settings, USA	C: Educational materials alone, referred to oncology nurse upon evaluation if distressed significantly<br>W: Trained graduates monitoring telephone calls. Referral on to an oncology nurse where indicated.		control (P<.0001), at 6 months controlling for study entry levels.<br>No differences on other measures of psychological distress.
Lapid *et al.* 2007[42]<br><br>Secondary age group analysis of Rummans et al. 2006[67]<br><br>RCT<br>++	Adults aged 65+ newly diagnosed with advanced cancer<br>n=33<br><br>Cancer centre, USA	I: Multidisciplinary psychosocial QOL sessions<br>T: Eight 90 min sessions, 4 weeks after enrollment<br>C: Standard care (regular outpatient visits with oncologist and allied health care providers)<br>W: Led by psychiatrist or psychologist and co-facilitated by a nurse, physical therapist, chaplain or social worker. Leaders trained in materials and observed sessions.	Quality of life	Higher overall QOL intervention group scores throughout the study, not significant.<br>Higher QOL scores at week 4 intervention v control (79.3 vs 62.9, P=.0461)<br>Improvement in QOL scores for intervention at week 4 and 8 compared to older control group.
Mantovani *et al.* 1996[29]<br><br>RCT<br>++	Adults aged 65+ with cancer<br>n=72<br><br>Inpatient setting at medical oncology clinic, Italy	I1. Emotional and practical support from volunteers and I2. with structured psychotherapy<br>T: I2: Weekly sessions of 1 hour for 6 months<br>C: Pharmacological only<br>W: Trained volunteers basic = 40 hrs/6 months, another 40 hrs/6 months practical and further personal training.	Quality of life	Non-significant between group differences in functional status/physical symptom improvements over time : Karnofsky's Performance Status Scale (F=9.90, 2 df, P<.001). No differences on Spitzer's QOL Index or Functional Living Index - within/between groups<br>Significant between group differences: State-Trait Anxiety Inventory control significantly worsened and intervention groups significantly improved (I1 improved more than I2) (F=4.50, df 2, P<.05).<br>Beck Depression Inventory: control group unchanged, both intervention groups improved (F=229.66, 2df, P< .01).
Sajid *et al.* 2016[43]<br><br>RCT (pilot)<br>++	Men aged 70+ with prostate cancer and hormone therapy<br>n=19<br><br>Two medical oncology clinics, USA	I1. EXCAP (home based walking and resistance intervention)<br>I2. Technology mediated walking and resistance intervention using Wi-Fit<br>T: One face to face session then 6-12 weeks home based<br>C: Usual care<br>W: Trained exercise physiologist	Functional and aerobic<br><br>(Skeletal muscle and muscular mass measure; handgrip strength; chest repetition test; DEXA scan)	EXCAP intervention arm higher rate of change in steps per day at each follow up (+2720 steps) (P < 0.01) compared to control (+97 steps) and Wii-fit arm (+382 non- significant.)<br>EXCAP arm had a 2.3 point change in physical battery score after 12 weeks, compared to 0.6 points in the Wii-Fit arm and −0.5 points in the usual-care arm.<br>No other significant differences in outcomes.
Suh *et al.* 2013[39]<br><br>RCT<br>+++	Adults aged 65+ completed active treatment for gastro-intestinal cancers<br>n=63<br><br>Cancer centre, South Korea	I: 8 weeks of Qi exercise and 1 hour face-to-face counselling on physical and psychological factors<br>T: 8 weeks<br>C: Usual care<br>W: Two Qi exercise trainers, APNs	Physical activity<br><br>(Body mass index; body weight; nutritional status; symptom experience; self-efficacy; self-esteem)	Physical activity increased in both groups, extent of increase greater in intervention group (P=.005). Difference in amount of exercise over time between groups (P=.002).<br>No between group difference in BMI.<br>Nutritional status in both groups improved over time. The degree of reduction, however, was significantly larger in intervention group (P=.048), and same in interaction between group and time.<br>Both group and interaction factors significant positive difference in symptom experience, health promotion and Self-esteem for intervention.
Yagli and Ulger 2015[36]<br><br>Controlled before and after study<br>++	Women age 65-70 6 months after chemotherapy for breast cancer<br>n=20<br><br>Department of	I: 8 sessions of 1 hr yoga classes<br>T: 8 weeks<br>C: Exercise programme for 8 weeks<br>W: Existing physiotherapist (yoga teacher)	Quality of life; depression levels; levels of pain, fatigue and sleep quality	All patients' QOL scores improved pre to post yoga and exercise interventions.<br>Total scores and some sub categories of the Nottingham Health Profile showed significant difference in favor of the yoga group (P<.05) but not on energy level and pain where no differences.<br>Significant better fatigue and sleep quality in yoga group post intervention (P<.05).

For peer review only

Source; Study design; Final quality rating	Setting and sample	Intervention and workforce	Primary outcomes (secondary outcomes)	Results
	physiotherapy and rehabilitation, Turkey			
**Communication between patients and health care professionals**				
Devik, Hellzen and Enmarker 2015[23]<br><br>Qualitative<br>++	Adults aged 65+ with advanced cancer<br>n=9<br><br>Patients homes in rural Norway	I: Qualitative study of home nursing care to advanced cancer patients in rural locations<br>C: n/a<br>W: District nurses in normal role	Patient experience	Importance of nurses having a person-centred manner.<br>Ability to show a genuine and empathic interest in the patients.<br>Technical skills or special competences less discussed than personal qualities, such as having a sense of humour or generosity.<br>Good listening and communication skills.
van Weert *et al.* 2011[21]<br><br>RCT (cluster)<br>++++	Adults aged 65+ with cancer receiving chemotherapy<br>n=210<br><br>Staff: oncology trained nurses n=77<br><br>12 wards of 10 hospitals, Netherlands	I: Workforce: communication skills training in delivery of chemotherapy education to patients<br>T: 3 month implementation<br>C: Nurses continued to provide patient education as usual<br>W: Nursing and specialised oncology nursing roles.	Effects on quality of staff communication; effects on content of the consultation<br><br>(Patient recall of information)	Significant improvement in discussing realistic expectations C: -0.20; I: 0.45 (total between group difference 0.65) (P<.01).<br>Significant decrease in rehabilitation information pre to post change C:0.08, I: -0.38 (total between group difference -0.45) (P<.01).<br>No significant changes in categories treatment-related information and coping information.<br>Non sig: intervention group showed significant decrease in number of items discussed.<br>Less history taking pre to post (C: 1.83; I: -2.33; between group difference - 4.17; p<.001) and less talking about all different side effects pre to post change (C: 1.98; I: -5.71; total 7.68; P<.001).<br>Patients in intervention asked more questions (M= 10.76) than control (M= 6.69; P<.05).<br>Marginal significant for intervention group: Proportion recall of recommendations post v pre. (C: -3.34; I: 6.39; Total: 9.73; P<.10).
Yeom and Heidrich 2013[24]<br><br>Observational<br>++	190 women at least one year post breast cancer diagnosis n=190<br><br>Community, an oncology clinic, and a state tumor registry, USA	I: Symptom management (IRIS): counselling interview and telephone follow up on symptom management<br>T: 8 week intervention with 16 week follow up point in the RCT.<br>C: Wait-list control subjects offered intervention after 16-week follow-up assessment<br>W: Advanced practice nurses	Negative beliefs about symptom management<br><br>(Quality of lIfe; purpose in life; positive relations with others)	Significant direct effects on Symptom Management Beliefs Questionnaire (SMBQ) (P<.00) and Communication Attitudes Questionnaire (P .012) on Communication Difficulties Questionnaire<br>Communication Difficulties significant direct, negative effects on all four dimensions of quality of life<br>Significant total effects of SMBQ on MCS (mental quality of life) (P=.001) and Purpose on Life (PL) and Personal Relations (PR) (P<.001) but not Physical component (PCS) .SMBQ predicted lower levels of quality of life in three of four dimensions.<br>None of the four indirect effects of SMBQ on quality of life through CommD was significant, indicating that CommD does not mediate the effects of SMBQ on quality of life<br>The total effects of CommA on four quality of life measures were not significant. However, the indirect effects for MCS (P=.05), PIL (P<.05) and PR (P<.05) through CommD were significant, indicating that CommD mediates the effects of CommA on MCS, PIL and PR.

Quality ratings: High ++++; Moderate +++; Low ++; Very low + Intervention/workforce description: I= Intervention; T= Timepoint; C= Control group; W: Workforce involved

For peer review only - http://bmjopen.bmj.com/site/about/guidelines.xhtml

Funding statement

This work was funded by Macmillan Cancer Support to support the work of the Expert Reference Group for the Older Person with Cancer. The views expressed are those of the authors and do not necessarily reflect those of Macmillan Cancer Support.

Contributors: JB, GL, PG and TW were responsible for the systematic review design. GL was responsible for data collection. JB, GL and PG were responsible for data extraction and appraising studies. JB, GL, PG and TW were responsible for data analysis and interpretation. All authors contributed in drafting the manuscript. JB is responsible for the overall content as the corresponding author.

None of the named authors has a conflict of interest in relation to this work or its publication.

Data sharing statement No additional unpublished data are available.

Reporting Guidelines: Prisma checklist (supplementary file 3)

For peer review only

REFERENCES

1. Yancik R, Ries LAG. Cancer in older persons: an international issue in an aging world. Seminars in Oncology;**31**(2):128-36.

2. Coleman MP, Forman D, Bryant H, et al. Cancer survival in Australia, Canada, Denmark, Norway, Sweden, and the UK, 1995–2007 (the International Cancer Benchmarking Partnership): an analysis of population-based cancer registry data. The Lancet;**377**(9760):127-38.

3. International Society of Geriatric Oncology. SIOG 10 Priorities Initiative. SIOG, 2011. http://www.siog.org/content/siog-10-priorities-initiative. (Accessed 24.03.2017)

4. Lynch MP, DeDonato DM, Kutney-Lee A. Geriatric Oncology Program Development and Gero-Oncology Nursing. Seminars in Oncology Nursing 2016;**32**(1):44-54.

5. Prouse J, Phillips J. Care of older people living with cancer: The role of the specialist nurse and alied health profesionals. Cancer Forum 2013;**37**(3):226-29.

6. Bridges J, Hughes J, Farrington N, et al. Cancer treatment decision-making processes for older patients with complex needs: a qualitative study. BMJ Open 2015;**5**(12):e009674.

7. Balducci L, Extermann M. Management of cancer in the older person: a practical approach. The Oncologist 2000;**5**(3):224-37.

8. Caillet P, Laurent M, Bastuji-Garin S, et al. Optimal management of elderly cancer patients: usefulness of the Comprehensive Geriatric Assessment. Clinical Interventions In Aging 2014;**9**:1645-60.

9. Expert Reference Group for the Older Person with Cancer. Older people living with cancer: designing the future health care workforce. Macmillan Cancer Support, 2016. http://sogweb-prod.neox24.ch/files/public/workforce-report.pdf (Accessed 24.03.2017)

10. Bridges J, Wengström Y, Bailey DE. Educational preparation of nurses caring for older people with cancer: an international perspective. Seminars in Oncology Nursing 2015;**32**(1):16-23.

11. Independent Cancer Taskforce. Achieving world-class cancer outcomes. A strategy for England 2015-2020. https://www.cancerresearchuk.org/sites/default/files/achieving_world-class_cancer_outcomes_-_a_strategy_for_england_2015-2020.pdf (Accessed 24.03.2017), 2015.

12. Lyman GH. Caring for the elderly cancer patient: training the next generation of oncologists. Journal Of Oncology Practice / American Society Of Clinical Oncology 2008;**4**(4):193-94.

13. Rao AV, Hurria A, Kimmick G, et al. Geriatric Oncology: Past, Present, Future. Journal of Oncology Practice 2008;**4**(4):190-92.

14. Maggiore RJ, Gorawara-Bhat R, Levine SK, et al. Perceptions, attitudes, and experiences of hematology/oncology fellows toward incorporating geriatrics in their training. Journal of Geriatric Oncology 2014;**5**(1):106-15.

15. Foubert J, Faithfull S. Education in Europe: Are cancer nurses ready for the future? Journal of BUON 2006;**11**(3):281-84.

16. McMillan S, Grodzicki BK, Shahrokni A. Building a Comprehensive Educational Model for Nurse Practitioner Students in Geriatric Oncology. Journal of the American Geriatrics Society 2016;**64**:S180-S80.

17. Bridges J, Lucas G, Wiseman T, et al. Preparedness of UK workforce to deliver cancer care to older people: summary report from a scoping review. University of Southampton, 2016. http://eprints.soton.ac.uk/401192/ (Accessed 24.03.2017).

18. Guyatt GH, Oxman AD, Vist GE, et al. GRADE: an emerging consensus on rating quality of evidence and strength of recommendations. BMJ: British Medical Journal 2008;**336**(7650):924.

19. Moher D, Liberati A, Tetzlaff J, et al. Preferred Reporting Items for Systematic Reviews and Meta-Analyses: The PRISMA Statement. PLOS Medicine 2009;**6**(7):e1000097.

20. Popay J, Roberts H, Sowden A, et al. Guidance on the conduct of narrative synthesis in systematic reviews. A product from the ESRC methods programme Version 1.: ESRC, 2006.

For peer review only

21. van Weert JC, Jansen J, Spreeuwenberg PM, et al. Effects of communication skills training and a Question Prompt Sheet to improve communication with older cancer patients: a randomized controlled trial. Critical reviews in oncology/hematology 2011; 80(1), 145-59.

22. Herr K, Titler M, Fine PG, et al. The effect of a translating research into practice (TRIP)--cancer intervention on cancer pain management in older adults in hospice. Pain Medicine (Malden, Mass) 2012; 13(8), 1004-17..

23. Devik SA, Hellzen O, Enmarker I. "Picking up the pieces" - Meanings of receiving home nursing care when being old and living with advanced cancer in a rural area. International Journal Of Qualitative Studies On Health And Well-Being 2015;**10**:28382-82.

24. Yeom HE, Heidrich SM. Relationships between three beliefs as barriers to symptom management and quality of life in older breast cancer survivors. Oncology nursing forum 2013; 40(3), E108-18.

25. Basu M, Linebarger J, Gabram SGA, et al. The effect of nurse navigation on timeliness of breast cancer care at an academic comprehensive cancer center. Cancer 2013;**119**(14):2524-31.

26. Fann JR, Fan MY, Unützer J. Improving primary care for older adults with cancer and depression. Journal of General Internal Medicine 2009; 24 Suppl 2.

27. Goodwin JS, Satish S, Anderson ET, et al. Effect of nurse case management on the treatment of older women with breast cancer. Journal of the American Geriatrics Society 2003; 51(9), 1252-9.

28. Kornblith AB, Dowell JM, Herndon JE, et al. Telephone monitoring of distress in patients aged 65 years or older with advanced stage cancer: a cancer and leukemia group B study. Cancer 2006; 107(11), 2706-14.

29. Mantovani G, Astara G, Lampis B, et al. Impact of psychosocial intervention on the quality of life of elderly cancer patients. Psycho-oncology 1996; 5(2), 127-35.

30. Hempenius L, Slaets JPJ, Asselt D, et al. Outcomes of a Geriatric Liaison Intervention to Prevent the Development of Postoperative Delirium in Frail Elderly Cancer Patients: Report on a Multicentre, Randomized, Controlled Trial. PloS One 2013; 8(6), e64834.

31. Rao AV, Hsieh F, Feussner JR. Geriatric evaluation and management units in the care of the frail elderly cancer patient. Journals of Gerontology: Series A, Biological Sciences and Medical Sciences, 2005; 60A(6), 798-803.

32. Somana-Ehrminger S, Dabakuyo TS, Manckoundia P, et al. Influence of geriatric oncology consultation on the management of breast cancer in older women: a French population-based study. Geriatrics & gerontology international 2015;**15**(1):111-9.

33. Deliens C, Deliens G, Filleul O, et al. Drugs prescribed for patients hospitalized in a geriatric oncology unit: Potentially inappropriate medications and impact of a clinical pharmacist. Journal Of Geriatric Oncology 2016; 7(6), 463-470.

34. Bourdel-Marchasson I, Blanc-Bisson C, Doussau A, et al. Nutritional advice in older patients at risk of malnutrition during treatment for chemotherapy: a two-year randomized controlled trial. PLoS One 2014; 9(9), e108687.

35. Mandelblatt J, Traxler M, Lakin P, et al. A nurse practitioner intervention to increase breast and cervical cancer screening for poor, elderly black women. The Harlem Study Team. Journal of General Internal Medicine 1993; 8(4), 173-8.

36. Yagli NV, Ulger O. The effects of yoga on the quality of life and depression in elderly breast cancer patients. Complementary therapies in clinical practice 2015; 21(1), 7-10.

37. McCorkle R, Strumpf NE, Nuamah IF, et al. A specialized home care intervention improves survival among older post-surgical cancer patients. Journal of the American Geriatrics Society 2000;**48**(12):1707-13.

38. Heidrich SM, Brown RL, Egan JJ, et al. An individualized representational intervention to improve symptom management (IRIS) in older breast cancer survivors: three pilot studies. Oncol Nurs Forum 2009;**36**(3):E133-43.

39. Suh EE, Kim H, Kang J, et al. Outcomes of a culturally responsive health promotion program for elderly Korean survivors of gastrointestinal cancers: a randomized controlled trial. Geriatric Nursing 2013; 34(6), 445-52.

For peer review only

40. Chock MM, Lapid MI, Atherton PJ, et al. Impact of a structured multidisciplinary intervention on quality of life of older adults with advanced cancer. International Psychogeriatrics 2013; 25(12), 2077-86.

41. Johansson B, Holmberg L, Berglund G, et al. Reduced utilisation of specialist care among elderly cancer patients: A randomised study of a primary care intervention. European Journal of Cancer 2001; 37(17), 2161-8.

42. Lapid MI, Rummans TA, Brown PD, et al. Improving the quality of life of geriatric cancer patients with a structured multidisciplinary intervention: a randomized controlled trial. Palliative & supportive care 2007; 5(2), 107-14.

43. Sajid S, Dale W, Mustian K, et al. Novel physical activity interventions for older patients with prostate cancer on hormone therapy: A pilot randomized study. Journal of Geriatric Oncology 2016; 7(2), 71-80.

44. Kalsi T, Babic-Illman G, Ross PJ, et al. The impact of comprehensive geriatric assessment interventions on tolerance to chemotherapy in older people. Br J Cancer 2015;**112**(9):1435-44.

45. Craig P, Dieppe P, Macintyre S, et al. Developing and evaluating complex interventions: the new Medical Research Council guidance. BMJ 2008;**337**:979-83.

46. Bridges J, Simcock R. Meeting the workforce challenges for older people living with cancer. International journal of nursing studies;**65**:A1-A2.

47. Kagan SH. The Future of Gero-Oncology Nursing. Semin Oncol Nurs 2016;**32**(1):65-76.

48. Levenson D. Advanced practice nurses lengthen survival of elderly cancer patients. Rep Med Guidel Outcomes Res 2001;**12**(5):5-7.

49. Morgan B, Tarbi E. The Role of the Advanced Practice Nurse in Geriatric Oncology Care. Semin Oncol Nurs 2016;**32**(1):33-43.

50. Prouse J. Geriatric assessment. The role of the specialist nurse practitioner. Asia-Pacific Journal of Clinical Oncology 2009;**5**:A155.

51. Extermann M, Aapro M, Audisio R, et al. Main priorities for the development of geriatric oncology: a worldwide expert perspective. Journal of Geriatric Oncology 2011;**2**(4):270-73.

52. Gosney M. Contribution of the geriatrician to the management of cancer in older patients. European Journal of Cancer 2007;**43**(15):2153-60.

53. Magnuson A, Dale W, Mohile S. Models of Care in Geriatric Oncology. Current Geriatrics Reports 2014;**3**(3):182-89.

54. Puts M, Santos B, Hardt J, et al. An update on a systematic review of the use of geriatric assessment for older adults in oncology. Annals of Oncology 2013; 25(2), 307-15.

55. Hamaker ME, Schiphorst AH, ten Bokkel Huinink D, et al. The effect of a geriatric evaluation on treatment decisions for older cancer patients–a systematic review. Acta Oncologica 2014;**53**(3):289-96.

56. Extermann M, Hurria A. Comprehensive geriatric assessment for older patients with cancer. Journal of Clinical Oncology 2007;**25**(14):1824-31.

57. Lycke M, Pottel L, Boterberg T, et al. Integration of geriatric oncology in daily multidisciplinary cancer care: The time is now. European Journal of Cancer Care 2015;**24**(2):143-46.

58. Chapman AE, Swartz K, Schoppe J, et al. Development of a comprehensive multidisciplinary geriatric oncology center, the Thomas Jefferson University Experience. Journal Of Geriatric Oncology 2014;**5**(2):164-70.

59. Bouzereau V, Le Caer F, Guardiola E, et al. Experience of multidisciplinary assessment of elderly patients with cancer in a French general hospital during 1 year: a new model care study. Journal Of Geriatric Oncology 2013;**4**(4):394-401.

60. Terret C, Zulian GB, Naiem A, et al. Multidisciplinary approach to the geriatric oncology patient. Journal of Clinical Oncology 2007;**25**(14):1876-81.

61. Mbah O, Ford JG, Qiu M, et al. Mobilizing social support networks to improve cancer screening: The COACH randomized controlled trial study design. BMC Cancer 2015; 15(1), 907.

For peer review only

62. Fraser M. Thinking differently about the cancer workforce. 2016. http://www.nuffieldtrust.org.uk/blog/thinking-differently-about-cancer-workforce. (Accessed 24.03.2017)

63. Glajchen M. Education, Training, and Support Programs for Caregivers of Individuals with Cancer. In: Talley RC, McCorkle R, Baile WF, eds. Cancer Caregiving in the United States: Research, Practice, Policy: Rosalynn Carter Institute for Caregiving Caregiving: Research-Practice-Policy series. New York and Heidelberg: Springer, 2012:79-102.

64. Muss HB. Cancer in the Elderly: a Societal Perspective from the United States. Clin Oncol 2009;**21**(2):92-98.

65. Brodie H. Getting the skill mix right. Nursing Older People 2013;**25**(6):10-10.

66. Clark MM, Rummans TA, Atherton PJ, et al. Randomized Controlled Trial of Maintaining Quality of Life During Radiation Therapy for Advanced Cancer. Cancer 2013;**119**(4):880-87.

67. Rummans TA, Clark MM, Sloan JA, et al. Impacting quality of life for patients with advanced cancer with a structured multidisciplinary intervention: a randomized controlled trial. Journal of clinical oncology : official journal of the American Society of Clinical Oncology 2006;**24**(4):635-42.

For peer review only

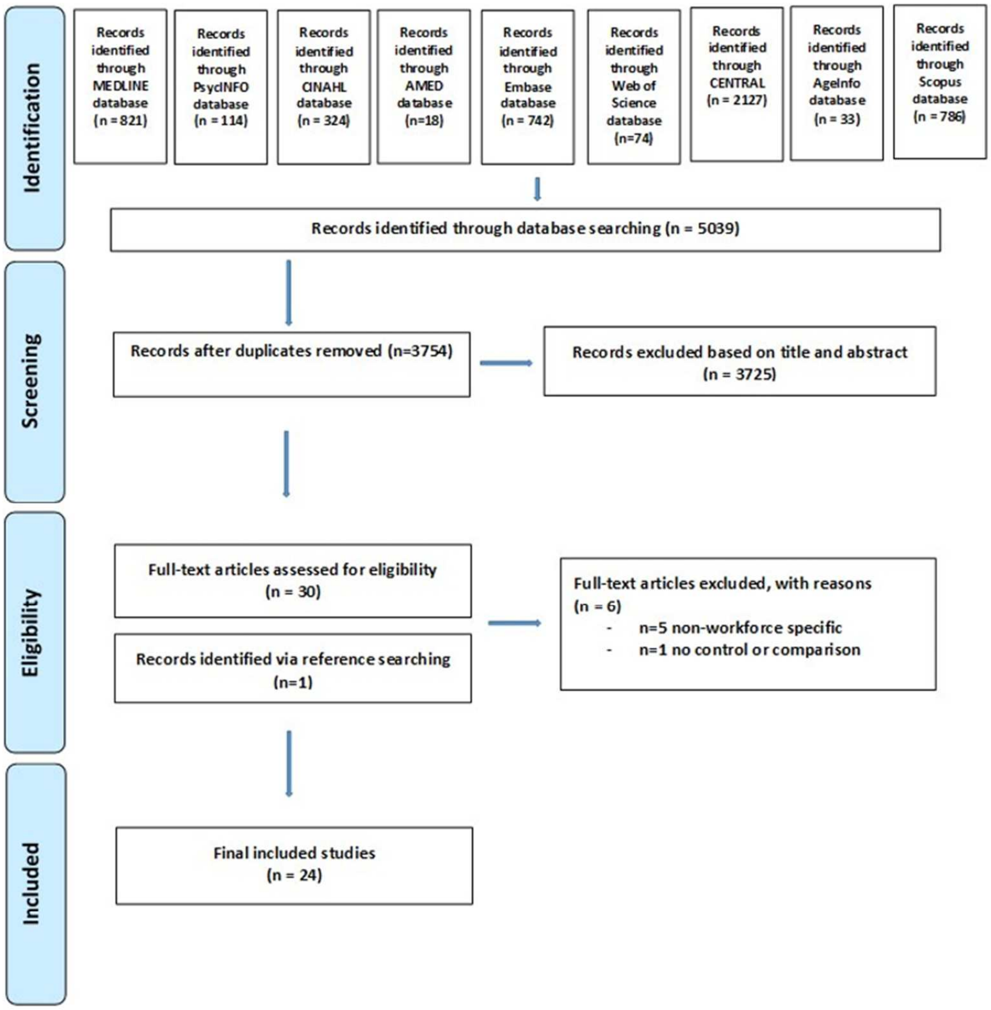
Identification
Records identified through MEDLINE database (n = 821)
Records identified through PsycINFO database (n = 114)
Records identified through CINAHL database (n = 324)
Records identified through AMED database (n=18)
Records identified through Embase database (n = 742)
Records identified through Web of Science database (n=74)
Records identified through CENTRAL (n = 2127)
Records identified through AgeInfo database (n = 33)
Records identified through Scopus database (n = 786)
Records identified through database searching (n = 5039)
Screening
Records after duplicates removed (n=3754)
Records excluded based on title and abstract (n = 3725)
Eligibility
Full-text articles assessed for eligibility (n = 30)
Records identified via reference searching (n=1)
Full-text articles excluded, with reasons (n = 6)
- n=5 non-workforce specific
- n=1 no control or comparison
Included
Final included studies (n = 24)

SUPPLEMENTARY FILE 1

Full search strategy

EBSCO HOST
Medline, CINHAL, PsycINFO, AMED: text terms and MeSH headings

1. TI Elderly OR AB Elderly
2. TI Geriatric* OR AB Geriatric*
3. TI "Older people" OR AB "Older people"
4. TI "Older patient*" OR AB "Older patient*"
5. TI "Older person" OR AB "Older person"
6. TI "Older adult*" OR AB "Older adult*"
7. MM Aged
8. MM Frail Elderly
9. **1 or 2 or 3 or 4 or 5 or 6 or 7 or 8**

10. TI Cancer OR AB Cancer
11. TI Oncolog* OR AB Oncolog*
12. MM Neoplasms
13. **10 or 11 or 12**

14. TI Workforce OR AB Workforce
15. TI "Health professionals" OR AB "Health professionals"
16. TI "Healthcare professionals" OR AB "Healthcare professionals"
17. TI "Health care professionals" OR AB "Health care professionals"
18. TI "Health personnel" OR AB "Health personnel"
19. TI "Healthcare personnel" OR AB "Healthcare personnel"
20. TI "Health care personnel" OR AB "Health care personnel"
21. TI "Medical personnel" OR AB "Medical personnel"
22. TI "Advanced Practice nurse" OR AB "Advanced Practice Nurse"
23. TI "Clinical nurse specialist" OR AB "clinical nurse specialist"
24. TI Geriatrician* OR AB Geriatrician*
25. TI Gerontologist* OR AB Gerontologist*
26. TI "Allied health professionals" OR AB "Allied health professionals"
27. TI Training
28. TI Educat*
29. TI "Skill mix" OR AB "Skill mix"
30. TI "Grade mix" OR AB "Grade mix"
31. TI "Staff development" OR AB "Staff development"
32. TI Staff* W1 level* OR AB Staff* W1 level*
33. TI Teamwork OR AB Teamwork
34. MM Health manpower
35. MM Health personnel
36. MM Attitude of Health personnel
37. MM Professional Competence
38. MM Staff development
39. MM Education, professional
40. MM Nurse's role
41. MM Geriatric assessment

For peer review only

42. MM Health services for the aged
43. or/ 14-42
44. 9 AND 13 AND 43
45. English language filter

Embase (OVID)

1. elderly.ti,ab

2. geriatric.ti,ab

3. "older people".ti,ab.

4. "older patient*".ti,ab.

5. "older adult*".ti,ab.

6. "older person".ti,ab.

7. 1 or 2 or 3 or 4 or 5 or 6

8. cancer.ti,ab.

9. oncolog*.ti,ab.

1o. Neoplasm/

11. 8 or 9 or 10

12. workforce.ti,ab.

13. "health professionals".ti,ab.

14. "healthcare professional*".ti,ab.

15. "health care professional*".ti,ab.

16. "health personnel".ti,ab.

17. "healthcare personnel".ti,ab.

18. "health care personnel".ti,ab.

19. "medical personnel".ti,ab.

20. "Advanced Practice nurse".ti,ab.

21. "Clinical nurse specialist".ti,ab.

22. Geriatrician*.ti,ab.

23. Gerontologist*.ti,ab.

24. "Allied health professionals".ti,ab.

25. training.ti

For peer review only

26. educat*.ti

27. Skill Adj1 mix.ti,ab.

28. Grade Adj1 mix.ti,ab.

29. “staff development”.ti,ab.

30. “Workforce development”.ti,ab.

31. Staff adj1 level.ti,ab.

32. health care manpower/

33 professional competence/

34 vocational education/

35. health personnel attitude/

36. or/12-35

37. English.lg.

38. 7 AND 11 AND 36 AND 37

Scopus (Elsevier)

TITLE-ABS-KEY (cancer OR oncology OR neoplasm*) AND TITLE-ABS-KEY (elderly OR geriatric OR "older people" OR "older adult*" OR "older patient*" OR "older person") AND TITLE-ABS-KEY (workforce OR "health professional*" OR "health care professional*" OR “healthcare professional” OR "health personnel" OR "medical personnel" OR "advanced practice nurse" OR "clinical nurse specialist" OR geriatrician OR gerontologist OR "skill mix" OR "staff development" OR manpower)) AND (LIMIT-TO (LANGUAGE , "English"))

Web of Science (Thompson Reuters)

TITLE: (cancer OR oncolog* OR neoplasm*) AND TITLE: (elderly OR geriatric* OR "older people" OR "older person" OR "older adult*" OR "older patient*") AND TITLE: ("workforce" OR "health professional*" OR "healthcare professional*" OR “health care professional” OR "health personnel" OR "medical personnel" OR "advanced nurse practitioner" OR "clinical nurse specialist" OR geriatrician OR gerontologist OR "allied health professional*" OR training OR "educat*" OR "skill mix" OR "grade mix" OR "staff development")

Refined by: LANGUAGES: (ENGLISH)

Timespan=All years

CENTRAL

Elderly OR geriatric OR “older people” OR “Older adult*” OR “older patient*” OR “older person”

AND cancer OR oncolog* OR neoplasm*

In Title OR Abstract OR Key Words

Search for Trials

AgeInfo Cancer or Oncology in Title

For peer review only

SUPPLEMENTARY FILE 2 DATA EXTRACTION TABLE WITH EXAMPLE

STUDY AND AIM	STUDY DESIGN DATA COLLECTION MEASURES ANALYSIS	SETTING AND SAMPLE<br><br>COUNTRY CLINICAL SETTING PATIENT GROUPS STAFF GROUP	INTERVENTION	OUTCOMES	STAFF ROLE OR WORKFORCE DEVELOPMENT	RESULTS
Basu *et al.* (2013)<br><br>To evaluate timeliness in the period from breast cancer diagnosis to initial oncology consultation for a 9-month period prior to and following implementation of the nurse navigator.	Before and after study<br><br>Retrospective chart review of demographic data, tumour grade and stage, cancer treatment, appointment information, navigation status, time interval from diagnosis date to initial oncology appointment date.<br><br>Chi-square tests of independence used to compare by navigation group, and t tests used to compare continuous variables. Multiple linear	USA<br><br>One cancer centre<br><br>All women diagnosed within the institution with stage 0 to III breast cancer at initial presentation<br><br>176 F: 100 (57%) in prenavigation group) and 76 (43%) in post navigation group<br><br>Stratified by age <61 and 61+<br><br>In the 61+ age group there were 86 patients 52 in the pre navigation group 34 in the post	A dedicated breast cancer nurse navigator to assist patients with their care. The navigator's involvement begins when the patient is diagnosed and ends when the patient is appointed into the survivorship clinic.	The time interval from date of diagnosis to date of initial oncology appointment was measured in business days. Date of diagnosis was defined as the patient's first positive biopsy date. The date of initial oncology appointment was defined as the first date the patient had a consultation with a surgical, medical, or radiation oncologist. In a few	Collaborating with physicians and interdisciplinary team members to triage patients, coordinating patient care, educating patients and providing links to research and other relevant resources, and serving as the primary point of contact for patients and their families. The nurse navigator calls recently diagnosed patients within 24 hours of biopsy, meets patients at the initial visit, and assists patients in scheduling oncology	Overall, the median wait time was 10 days for all patients in the study and ranged from 1 to 32 days. The median wait time for patients in the prenavigation group was 11 days compared with 9 days for patients in the postnavigation group.<br><br>Because of interaction between navigation and age identified during the modeling process, stratified models presented for each of the 2 categories of age<br><br>For patients aged 61

SUPPLEMENTARY FILE 2 DATA EXTRACTION TABLE WITH EXAMPLE

STUDY AND AIM	STUDY DESIGN DATA COLLECTION MEASURES ANALYSIS	SETTING AND SAMPLE<br><br>COUNTRY CLINICAL SETTING PATIENT GROUPS STAFF GROUP	INTERVENTION	OUTCOMES	STAFF ROLE OR WORKFORCE DEVELOPMENT	RESULTS
	regression used to determine influence on time to consultation.	navigation group<br><br>Breast cancer nurse navigator		cases a phone call from the oncologist to discuss pathology results and treatment options was recorded as the consultation date if there was no other consultation before treatment.	appointments or any necessary diagnostic tests. Patients are then tracked during the course of care to ensure timeliness of appointments and therapies and to provide referrals to treatment and supportive services as needed.	years and older, the median wait time was 4 days shorter for the navigation group (8 days versus 12 days).<br><br>In the 61 or older age group, navigation was a significant predictor of time to consultation, adjusting for demographic and clinical factors (P<.0002). In this adjusted model, the unstandardized beta coefficient for navigation was 24.9, indicating that with navigation, time to consultation decreased by an average of almost 5 days

For peer review only

For peer review only

Section/topic	#	Checklist item	Reported on page #
**TITLE**			
Title	1	Identify the report as a systematic review, meta-analysis, or both.	1
**ABSTRACT**			
Structured summary	2	Provide a structured summary including, as applicable: background; objectives; data sources; study eligibility criteria, participants, and interventions; study appraisal and synthesis methods; results; limitations; conclusions and implications of key findings; systematic review registration number.	2
**INTRODUCTION**			
Rationale	3	Describe the rationale for the review in the context of what is already known.	4
Objectives	4	Provide an explicit statement of questions being addressed with reference to participants, interventions, comparisons, outcomes, and study design (PICOS).	4
**METHODS**			
Protocol and registration	5	Indicate if a review protocol exists, if and where it can be accessed (e.g., Web address), and, if available, provide registration information including registration number.	7
Eligibility criteria	6	Specify study characteristics (e.g., PICOS, length of follow-up) and report characteristics (e.g., years considered, language, publication status) used as criteria for eligibility, giving rationale.	5-6
Information sources	7	Describe all information sources (e.g., databases with dates of coverage, contact with study authors to identify additional studies) in the search and date last searched.	4
Search	8	Present full electronic search strategy for at least one database, including any limits used, such that it could be repeated.	5
Study selection	9	State the process for selecting studies (i.e., screening, eligibility, included in systematic review, and, if applicable, included in the meta-analysis).	6
Data collection process	10	Describe method of data extraction from reports (e.g., piloted forms, independently, in duplicate) and any processes for obtaining and confirming data from investigators.	6
Data items	11	List and define all variables for which data were sought (e.g., PICOS, funding sources) and any assumptions and simplifications made.	4-6

For peer review only

Risk of bias in individual studies	12	Describe methods used for assessing risk of bias of individual studies (including specification of whether this was done at the study or outcome level), and how this information is to be used in any data synthesis.	6
Summary measures	13	State the principal summary measures (e.g., risk ratio, difference in means).	6
Synthesis of results	14	Describe the methods of handling data and combining results of studies, if done, including measures of consistency (e.g., I^2) for each meta-analysis.	6-7

Section/topic	#	Checklist item	Reported on page #
Risk of bias across studies	15	Specify any assessment of risk of bias that may affect the cumulative evidence (e.g., publication bias, selective reporting within studies).	7
Additional analyses	16	Describe methods of additional analyses (e.g., sensitivity or subgroup analyses, meta-regression), if done, indicating which were pre-specified.	N/A
**RESULTS**			
Study selection	17	Give numbers of studies screened, assessed for eligibility, and included in the review, with reasons for exclusions at each stage, ideally with a flow diagram.	Figure 1
Study characteristics	18	For each study, present characteristics for which data were extracted (e.g., study size, PICOS, follow-up period) and provide the citations.	Table 2
Risk of bias within studies	19	Present data on risk of bias of each study and, if available, any outcome level assessment (see item 12).	Table 2
Results of individual studies	20	For all outcomes considered (benefits or harms), present, for each study: (a) simple summary data for each intervention group (b) effect estimates and confidence intervals, ideally with a forest plot.	Table 2
Synthesis of results	21	Present results of each meta-analysis done, including confidence intervals and measures of consistency.	N/A
Risk of bias across studies	22	Present results of any assessment of risk of bias across studies (see Item 15).	7-11, Table 2
Additional analysis	23	Give results of additional analyses, if done (e.g., sensitivity or subgroup analyses, meta-regression [see Item 16]).	N/A
**DISCUSSION**			
Summary of evidence	24	Summarize the main findings including the strength of evidence for each main outcome; consider their relevance to key groups (e.g., healthcare providers, users, and policy makers).	11-12

For peer review only

Limitations	25	Discuss limitations at study and outcome level (e.g., risk of bias), and at review-level (e.g., incomplete retrieval of identified research, reporting bias).	3, 11-12
Conclusions	26	Provide a general interpretation of the results in the context of other evidence, and implications for future research.	11-12
**FUNDING**			
Funding	27	Describe sources of funding for the systematic review and other support (e.g., supply of data); role of funders for the systematic review.	19

For peer review only
